# Acridine‐Functionalized Covalent Organic Frameworks (COFs) as Photocatalysts for Metallaphotocatalytic C−N Cross‐Coupling

**DOI:** 10.1002/anie.202117738

**Published:** 2022-03-23

**Authors:** Michael Traxler, Sebastian Gisbertz, Pradip Pachfule, Johannes Schmidt, Jérôme Roeser, Susanne Reischauer, Jabor Rabeah, Bartholomäus Pieber, Arne Thomas

**Affiliations:** ^1^ Department of Chemistry/Functional Materials Technische Universität Berlin Hardenbergstraße 40 10623 Berlin Germany; ^2^ Department of Biomolecular Systems Max Planck Institute of Colloids and Interfaces Am Mühlenberg 1 14476 Potsdam Germany; ^3^ Department of Chemistry and Biochemistry Freie Universität Berlin Takustraße 3 14195 Berlin Germany; ^4^ Department of Chemical, Biological & Macro-Molecular Sciences S. N. Bose National Centre for Basic Sciences Kolkata 700106 India; ^5^ Leibniz Institute for Catalysis (LIKAT Rostock) Universität Rostock 18059 Rostock Germany

**Keywords:** Acridine, C−N Cross-Coupling, Catalysis, Covalent Organic Frameworks, Photoredox

## Abstract

Covalent organic frameworks (COFs) are structurally tuneable, porous and crystalline polymers constructed through the covalent attachment of small organic building blocks as elementary units. Using the myriad of such building blocks, a broad spectrum of functionalities has been applied for COF syntheses for broad applications, including heterogeneous catalysis. Herein, we report the synthesis of a new family of porous and crystalline COFs using a novel acridine linker and benzene‐1,3,5‐tricarbaldehyde derivatives bearing a variable number of hydroxy groups. With the broad absorption in the visible light region, the COFs were applied as photocatalysts in metallaphotocatalytic C−N cross‐coupling. The fully β‐ketoenamine linked COF showed the highest activity, due to the increased charge separation upon irradiation. The COF showed good to excellent yields for several aryl bromides, good recyclability and even catalyzed the organic transformation in presence of green light as energy source.

## Graphical abstract

A new family of porous crystalline COFs bearing acridine moieties was synthesized and applied as photocatalysts in metallaphotocatalytic C−N cross‐coupling. Among these materials the fully *β*‐ketoenamine‐linked COF showed the highest catalytic activity and was shown to be recyclable and even catalyzed the cross‐coupling efficiently under green light irradiation.

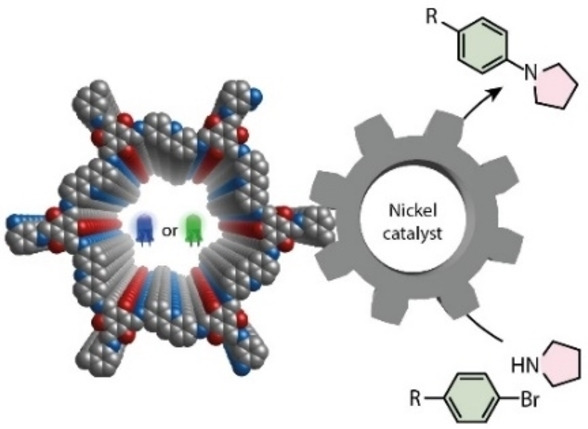

## Introduction

The field of covalent organic frameworks (COFs)—crystalline and porous polymers that are solely consisting of organic building blocks reticulated via covalent bonds—has gained significant attention in the last decade.[^1^, ^2^, ^3^, ^4^, ^5^, ^6^] A variety of building units (linkers) and organic reactions have been applied for the synthesis of COFs with a broad range of functionalities, linkages and variable pore structures.[^7^, ^8^, ^9^, ^10^, ^11^, ^12^, ^13^] Because these ordered structures have a permanent porosity, long‐range π‐conjugation, and the possibility to tune the structure of the backbone and integrate functional linkers, COFs have emerged as powerful materials for a plethora of different applications including gas storage and separation, energy storage, optoelectronics and catalysis.[^13^, ^14^, ^15^, ^16^, ^17^, ^18^, ^19^, ^20^, ^21^] The formation of strong covalent bonds between the organic building blocks results in high chemical stability for the framework materials that can be further enhanced by introducing linkers that allow for e.g. tautomerization or hydrogen bond formation.[^22^, ^23^]

As an impact of these properties, COFs are promising candidates for heterogeneous photocatalysis using visible‐light due to the long‐range π‐conjugation. Water splitting and CO_2_ reduction dominate this application branch, and only few examples using COF photocatalysts in organic synthesis were reported.^[24]^ These include oxidative hydroxylation,[^25^, ^26^] C−H functionalization, cross‐coupling reactions,[^27^, ^28^] oxidative N−S cyclization^[29]^ or tandem addition‐cyclization reaction.^[30]^ Recently, the scope of COFs in catalyzing organic transformation has been further expanded to C−S and C−O carbon heteroatom cross‐couplings through metallaphotocatalysis.[^31^, ^32^] However, COFs have not been applied in carbon‐nitrogen (C−N) cross‐coupling reactions, which are among the most important reactions in synthetic organic chemistry.^[33]^ The majority of COFs applied in photocatalysis are limited to short wavelengths (blue light radiation), which can result in deactivation of the nickel co‐catalyst,^[34]^ and other side‐reactions^[35]^ due to the high photon energy. These problems can be overcome using less energetic irradiation sources. Expanding the absorption of COFs in order to harvest long wavelengths requires, for example, increasing π‐conjugation by extending the length of the linkers with phenyl or acetylene groups, introduction of donor–acceptor structures, post‐synthetic introduction of a chromophore, or the use of organic dyes that absorb visible light as linkers.[^36^, ^37^, ^38^]

The acridine motif is commonly found in organic dyes and enables efficient intersystem crossing upon excitation. This results in long‐lived excited states that are crucial for efficient photocatalysis using low catalyst loadings.[^39^, ^40^] Recently, Stolarczyk and co‐workers showed that an acridine carbon dot heterostructure can be used for photocatalytic water splitting.^[41]^ Homogeneous acridines have been explored in dual photocatalysis for decarboxylative *N*‐alkylation, decarboxylative conjugate addition or dehydrocarboxylation of carboxylic acids.[^42^, ^43^, ^44^] Further, it has been observed that acridine based small molecules and materials can harvest lower energetic light radiation compared to anthracene compounds, especially in its protonated form.[^40^, ^41^, ^45^] However, utilization of acridine‐based linkers for the synthesis of crystalline and porous materials such as COFs and MOFs have not been attempted.

Herein, we describe the synthesis of novel COFs bearing acridine moieties in reticulation with three different benzene‐1,3,5‐tricarbaldehyde derivatives with a variable number of hydroxy groups. The resulting materials were evaluated for their application as photocatalysts in metallaphotocatalytic C−N cross‐couplings. Our results indicate that not only a high surface area and crystallinity, but also a *β*‐ketoenamine structure and high charge separation under light irradiation are the key factors for high catalytic activity.

## Results and Discussion

**Figure 1:**
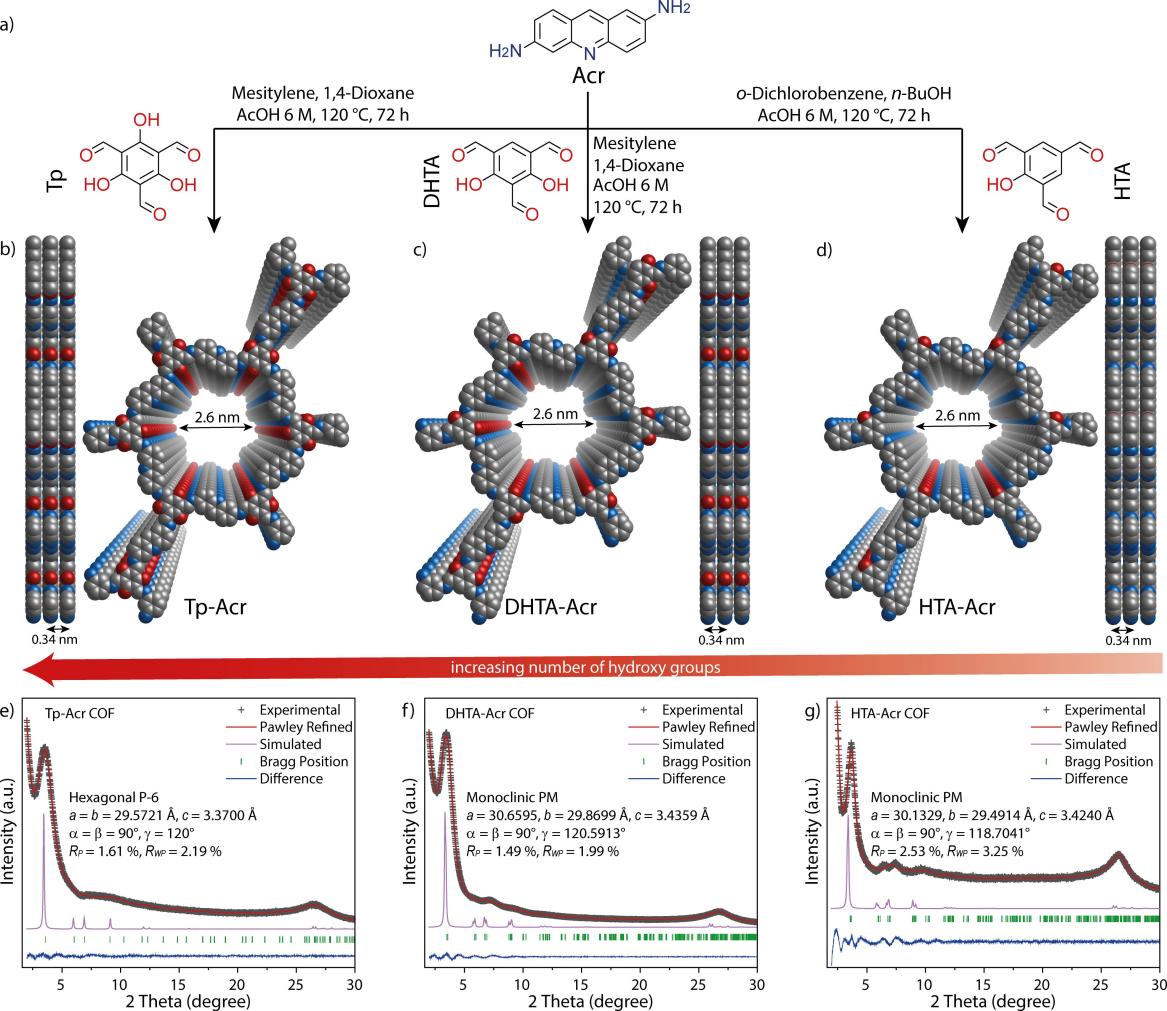
Synthesis and characterization of Tp‐Acr, DHTA‐Acr and HTA‐Acr. a) Scheme of the synthesis of the COFs. b–d) Top and side views of Tp‐Acr, DHTA‐Acr and HTA‐Acr showing the ideal eclipsed (AA) structures. e–g) Experimental, Pawley‐refined and simulated powder X‐ray diffraction patterns (AA stacking) and difference plot for Tp‐Acr, DHTA‐Acr and HTA‐Acr.

We began our investigations by synthesizing a C2‐linker bearing the acridine moiety (2,6‐diaminoacridine, Acr) by a three step reaction, where 3‐nitro‐*N*‐(4‐nitrophenyl)aniline was obtained by a Buchwald–Hartwig amination.^[46]^ After a palladium catalyzed reduction, the linker was prepared by a Bernthsen‐type acridine synthesis using formic acid [Section S3.1, in the Supporting Information].^[47]^ By changing the amounts of hydroxy groups, we have enabled a different *β*‐ketoenamine to imine ratio in Tp‐Acr, DHTA‐Acr and HTA‐Acr COFs (Figure 1a). In the special case of using the phloroglucinol based linker, the keto‐enol tautomerization is irreversible towards the keto form, whereas for DHTA and HTA based COFs the tautomerization shows reversibility.^[48]^ Tp‐Acr and DHTA‐Acr COFs were prepared via an acid catalyzed Schiff base reaction, where 2,6‐diaminoacridine (Acr, 31.5 mg, 0.15 mmol) was reacted with 1,3,5‐triformylphloroglucinol (Tp, 21 mg, 0.1 mmol) or 2,4‐dihydroxybenzene‐1,3,5‐tricarbaldehyde (DHTA, 19.4 mg, 0.1 mmol), respectively, using 6 M acetic acid (0.5 mL) as catalyst and a mixture of 3 mL mesitylene/dioxane (1 : 1) as a solvent. For the HTA‐Acr COF, the solvent mixture was changed to 1 : 1 *n*‐butanol/*o*‐dichlorobenzene (3 mL) in the reaction with 2‐hydroxybenzene‐1,3,5‐tricarbaldehyde (HTA, 17.8 mg, 0.1 mmol), while the amount of 2,6‐diaminoacridine and acetic acid was kept constant (Section S3.2). All the precursor mixtures were heated for 72 h at 120 °C and the solid product washed with acetone, methanol (MeOH) and cyclohexane prior to Soxhlet extraction using MeOH to obtain the COFs as dark red solids.

Structural features and crystallinity of the synthesized COFs were determined using powder X‐ray diffraction (PXRD) analyses with a Cu K_α_ radiation. All materials show the most intense reflections in the low angle region at 3.5 2*θ* degrees for Tp‐Acr as well as DHTA‐Acr, and 3.6 2*θ* degrees for HTA‐Acr (Figure 1e–g). These can be assigned to the (100) facet of a primitive hexagonal lattice. Additional weak reflections and a broad reflection at around 26.5 2*θ* degrees can be assigned to the (001) facet and confirm the crystalline π‐π stacked 2D structure for all three COFs. According to the geometry of the linkers, structural models with **hcb** topology were constructed for eclipsed (AA) and staggered (AB) stacking sequences (Figure S4–S6). After geometrical optimization, the theoretical PXRD patterns of the structures were calculated and compared to the experimental diffraction pattern. All three COFs showed good agreement for the eclipsed stacking pattern, whereas the simulated staggered pattern did not fit with the measured diffractogram (Figure S7). Additionally, a full Pawley refinement was carried out to fit the final unit cell parameters, which led to acceptably low residual values and profile differences (Figure 1e–g).

**Figure 2:**
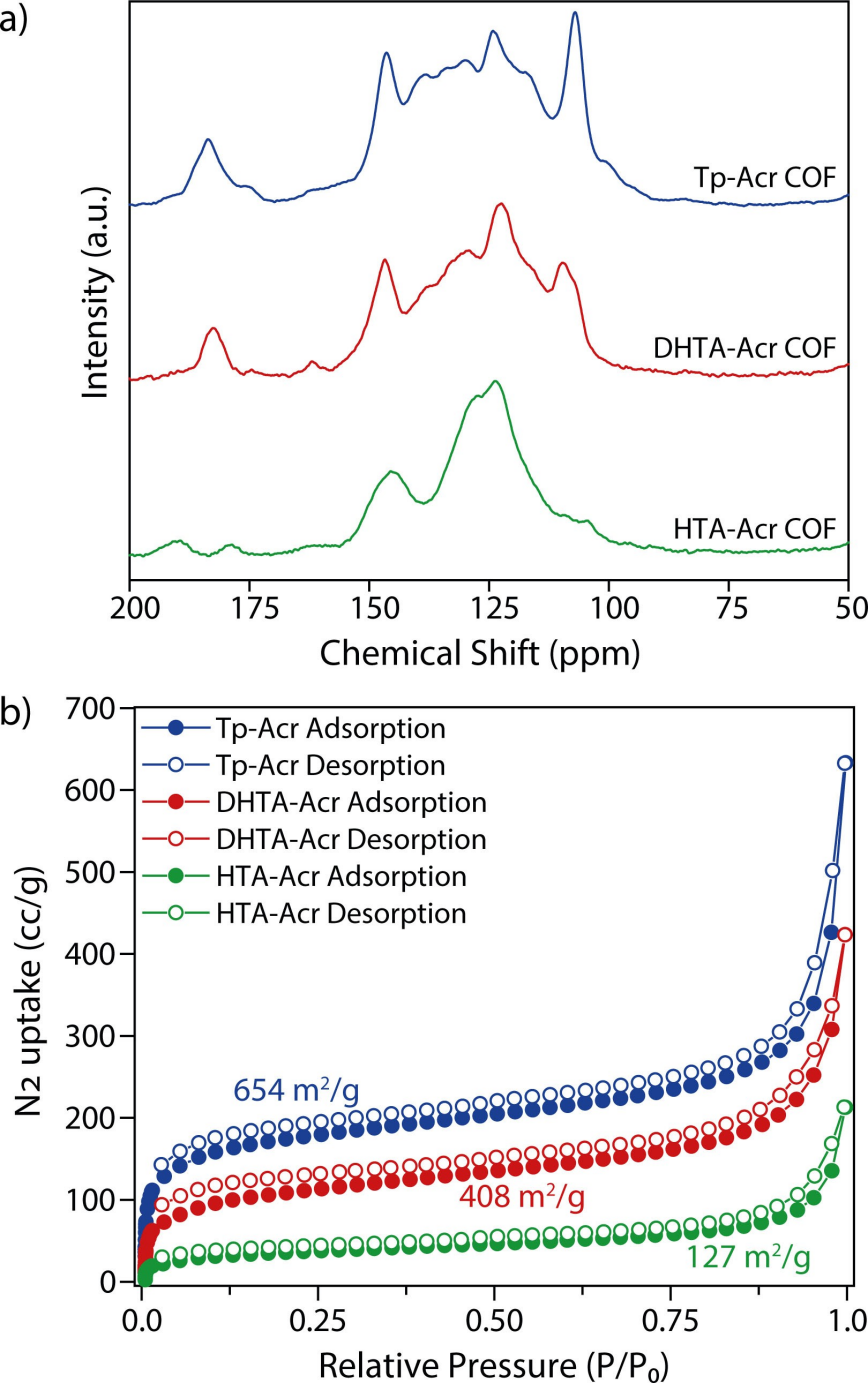
a) ^13^C CP‐MAS NMR spectra of Tp‐Acr, DHTA‐Acr and HTA‐Acr. b) N_2_ sorption isotherms for Tp‐Acr, DHTA‐Acr and HTA‐Acr.

All COFs were studied using ^13^C cross‐polarization magic angle spinning nuclear magnetic resonance (CP‐MAS NMR) analyses (Figure 2a). The pronounced carbonyl carbon (C=O) peak in the area of 180–185 ppm for Tp‐Acr and DHTA‐Acr indicates that these COFs exist dominantly in their keto form. In case of HTA‐Acr COF, an equilibrium between the keto‐ and enol‐form was identified via the signals at 179 and 189 ppm, respectively. Further distinctive peaks are overlapping with the broad multiple signals between 100–150 ppm due to the missing symmetry in the acridine linker. Fourier transform infrared (FT‐IR) spectra of the frameworks confirmed the disappearance of characteristic signals of the precursors, while diagnostic bands of the COFs (C=O and C=C bonds at 1550–1580 cm^−1^ and for C−N bonds at around 1270 cm^−1^) are present (Section S5.1). From the CP‐MAS NMR and FTIR data, the structural integrity and formation of acridine COFs has been validated.

The permanent porosity of the acridine based COFs was confirmed using nitrogen sorption measurements at 77 K (Figure 2b). The surface areas were calculated using the Brunauer–Emmett–Teller (BET) method. Among the newly synthesized acridine COFs, Tp‐Acr showed the highest BET surface area of 654 m^2^ g^−1^, compared to 408 m^2^ g^−1^ for DHTA‐Acr COF and 127 m^2^ g^−1^ for the HTA‐Acr COF. This shows that the number of hydroxy groups in the aldehyde linker influences the final accessible surface area of the COFs. The additional keto‐/enol groups within the COF structure work as pore‐directing “anchors”, improving the stacking of the layers, which results in the increase of surface area.^[49]^ Additional pore size evaluation revealed for all COFs a distribution close to the simulated one (Figure S11). Scanning electron microscopy (SEM) analyses revealed a rather undefined morphology from aggregated particles of Tp‐Acr, DHTA‐Acr and HTA‐Acr, while transmission electron microscopy (TEM) confirms the presence of a sheet like structure (Figure S12). In order to investigate the chemical stability of Tp‐Acr, the COF was immersed in various solvents. PXRD analyses of recovered samples confirmed, that the Tp‐Acr COF retained its crystalline structure after 3 days of treatment with acetone, methanol, cyclohexane, dimethylacetamide (DMAc) or water. Moreover, the COF was stable in a basic aqueous environment (1 M NaOH) for 1 day with only a slight loss in crystallinity. No sign of decomposition or dissolution was observed, and the COF could be recovered quantitatively after the treatment (Figure S13 and S14). Additionally, the thermal stability of the COFs was tested using thermogravimetric analysis (TGA), which revealed that the frameworks are—after initial weight loss due to adsorbed solvent molecules—thermally stable up to 300 °C (Figure S15). The chemical stability of the COF in DMAc and basic medium is important as the architectural stability in polar solvents and under basic conditions renders a prerequisite for many photocatalytic reactions, particularly metallaphotocatalytic cross‐couplings.^[50]^

**Figure 3:**
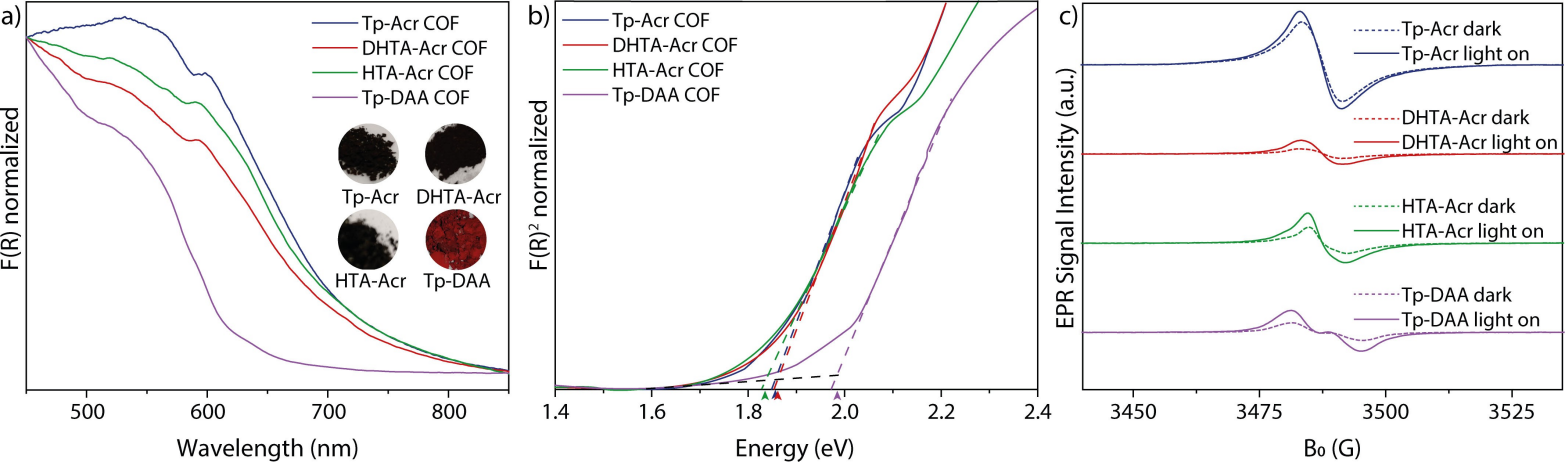
a) UV/Vis diffuse reflectance spectra for Tp‐Acr, DHTA‐Acr, HTA‐Acr and Tp‐DAA. The inset shows optical images of the COF powders. b) Tauc plots for Tp‐Acr, DHTA‐Acr, HTA‐Acr and Tp‐DAA. c) EPR conduction band e‐signals of Tp‐Acr, DHTA‐Acr, HTA‐Acr and Tp‐DAA under dark condition (dotted lines) and during visible light irradiation (>420 nm).

From the diffuse reflectance ultraviolet‐visible (UV/Vis) spectroscopy, it was confirmed that all acridine containing COFs show a very similar absorption behavior in the visible light region. In order to distinctly investigate the effect of the acridine moiety on the optical properties, an isoreticular COF with anthracene edges (Tp‐DAA, DAA: 2,6‐diaminoanthracene) was synthesized for comparison (Section S6).^[51]^ It can be clearly seen that the absorption of Tp‐DAA COF is blue shifted compared to the acridine COFs. While Tp‐DAA exhibits an absorption edge at 620 nm, all acridine COFs show an absorbance over a broad range of the visible light region. The absorption edge is in all cases around 680 nm tailing up to more than 800 nm (Figure 3a). Optical band gaps calculated from Tauc plots are 1.82–1.83 eV for the acridine COFs and 1.98 eV for the anthracene analogue, confirming the impact of acridine moieties for light harvesting in the visible region (Figure 3b). The absorption of the acridine moiety itself determines the photophysical properties with a peak shoulder at 610 nm that can be attributed to aggregation of the acridine units, which is red shifted to the absorption of the acridine linker itself (Figure S16). Such a phenomenon, the formation of so‐called “*J*‐aggregates”, was previously reported for porphyrin COFs.^[52]^ Moreover photoluminescence measurements and steady‐state time‐resolved fluorescence life‐time measurements were performed (Section S5.6). The measurements reveal for individual decay components, that Tp‐Acr shows the highest life‐time compared to other acridine based COFs.

The conduction band electrons of the acridine COF species were monitored by electron paramagnetic resonance (EPR) spectroscopy. To visualize the charge separation and transfer properties, EPR spectra of the COFs have been recorded in the dark and under photocatalytic reaction conditions. All three acridine based COFs show a singlet signal with Lorentzian line shape at *g*=2.007, which can be attributed to unpaired electrons in the conduction band.^[53]^ The signal intensities increased upon irradiation with light, since more electrons are excited from the valence band to the conduction band, indicating the formation of electron hole pairs in the COF semiconductors (Figure 3c).[^36^, ^54^] A clear trend in signal intensity can be found for the COF materials. The Tp‐Acr COF shows by far the highest signal intensity, which suggests that the charge separation efficiency is largely improved in the fully β‐keto tautomerized COF material. For DHTA‐Acr and HTA‐Acr the tautomerization between keto and enol form is likely to result in a decreased stability of the conduction band electrons. Compared to the structurally identical Tp‐Acr, the anthracene containing Tp‐DAA (*g*=2.007) COF shows a decreased efficiency in charge separation (Figure S18), highlighting the benefit of introducing the acridine moiety into the framework structure.

**Figure 4:**
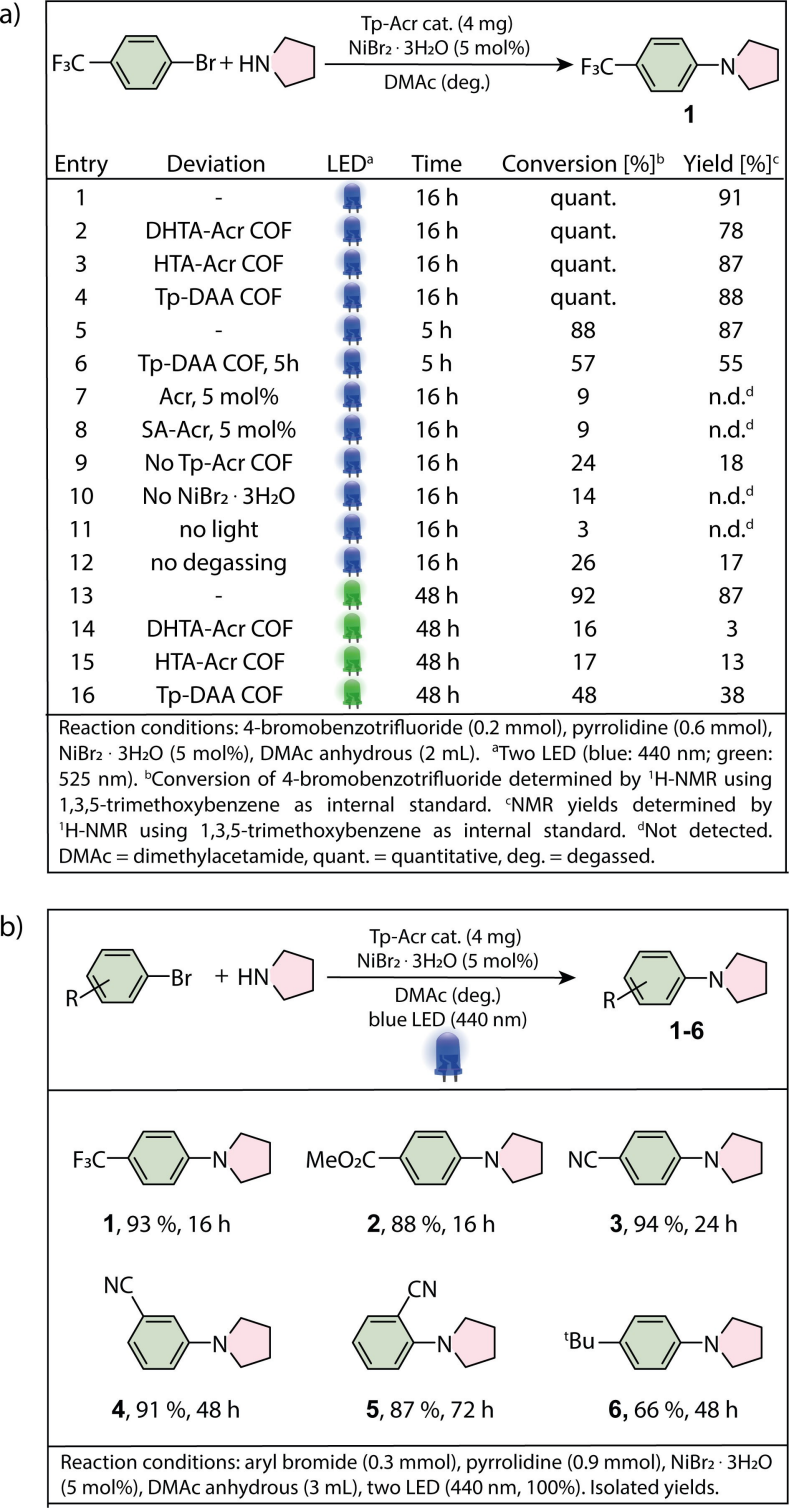
a) Optimized conditions and control experiments using blue and green light. b) Scope of the semi‐heterogeneous amination of pyrrolidine and aryl bromides.

After confirming the porosity as well as the presence of acridine functionalities in the COF backbones and determining the enhanced light absorption in visible light region as well as the charge separation properties, we sought to study if acridine COFs are suitable photocatalysts for semi‐heterogeneous dual nickel/photocatalytic C−N cross‐coupling.^[50]^ Our investigations started by optimizing the amination of 4‐bromobenzotrifluoride with pyrrolidine using the Tp‐Acr COF as photocatalyst and 440 nm LEDs as light source. Nearly quantitative formation (91 %) of the desired alkyl aryl amine (**1**) was obtained within 16 h when Tp‐Acr COF (2 mg mL^−1^), NiBr_2_ ⋅ 3H_2_O (5 mol%) and three equivalents of the amine coupling partner were used in DMAc (Figure 4, Entry 1). Similarly, DHTA‐Acr and HTA‐Acr showed full conversion of the substrate with slightly lower selectivity towards the coupling product (Entries 2 and 3). Interestingly, the isoreticular Tp‐DAA COF also gave almost quantitative formation of the desired product under these conditions (Entry 4). Shorter reaction times were investigated to better compare the activity of Tp‐Acr and Tp‐DAA. Within five hours, the acridine COF resulted in 87 % of the desired product, while Tp‐DAA gave only 55 % (Entries 5 and 6). These results highlight the advantage of using the acridine based COFs in metallaphotocatalytic C−N cross‐coupling and are in full agreement with the charge separation properties found in EPR. When comparing the three acridine COFs, also the higher surface area of Tp‐Acr compared to the others might accelerate the photocatalytic performance by increasing the number of accessible active sites and improving mass transfer within the material. However, Tp‐DAA shows an even higher surface area than TP‐Acr from BET measurements (Figure S20), but a lower activity, thus charge carrier generation and separation seem to be the most decisive parameter for their photocatalytic performance.

Control studies using the linker 2,6‐diaminoacridine (Acr) or a model compound (low molecular repeating unit of the COF structure; SA‐Acr; Section S7) as photocatalyst showed no formation of the product (Entries 7 and 8), emphasizing that only after incorporation into the COF backbone, the repeating units build up their photocatalytic properties through conjugation. In case of omitting a photocatalyst, still minor product formation (18 %) was detected (Entry 7). This can be explained by photoexcitation and productive catalysis of nickel‐amine complexes by the small portion of UV‐light in the emission of the used light source (Figure S1).^[55]^ Further control studies showed that a nickel source, light and an oxygen free‐environment are crucial for the desired coupling (Entry 10–12). With their extended absorption, the acridine COFs were additionally tested as photocatalyst under green light radiation (525 nm LEDs). This is important, because lower excitation energy prevents catalyst deactivation^[34]^ and the formation of undesired side products in case of certain substrates.^[35]^ Indeed, the reaction using Tp‐Acr as a photocatalyst resulted in 87 % yield of **1** after 48 h (Entry 13). On the contrary, DHTA‐Acr and HTA‐Acr showed only minor product formation, while using Tp‐DAA 38 % of the coupling product was obtained (Entries 14 and 16). These results emphasize that embedding the acridine unit into a fully keto tautomerized COF resulted in a highly efficient photocatalyst both under blue and green light irradiation. With the optimized conditions using the most efficient COF (Tp‐Acr) as photocatalyst, the versatility of the semi‐heterogeneous catalytic system was evaluated (Figure 4b). The reaction of pyrrolidine with electron deficient aryl bromides generally gave high yields for the corresponding aryl amines (**1**–**5**). An aryl bromide with electron‐donating group (**6**) reacted with slightly less selectivity, which is in agreement with most dual nickel/photocatalytic C−N coupling protocols.[^34^, ^55^, ^56^, ^57^, ^58^] Nitriles (**3**–**5**), ester (**2**), trifluoromethyl‐ (**1**) as well as a bulky aliphatic residue (**6**) were tolerated in the dual catalytic amination. Substrates with an electron withdrawing meta‐substituent (**4**) or ortho‐substituent (**5**) did also yield the desired products in similar selectivity, although with lower efficiency than the para‐substituted analogue (**3**).

**Figure 5:**
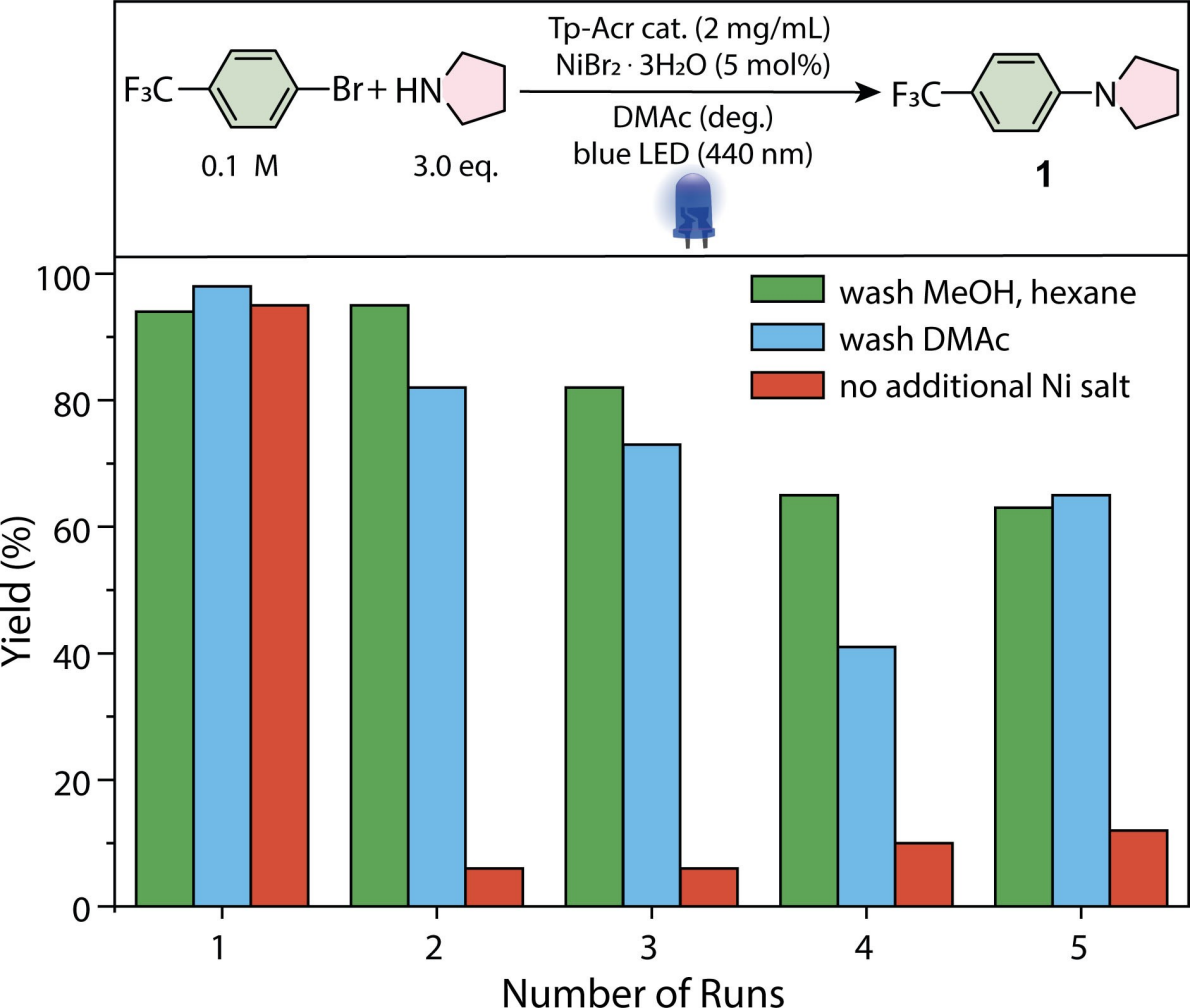
Reusability of Tp‐Acr COF in the dual nickel/photocatalytic amination of 4‐bromobenzotrifluoride and pyrrolidine (NMR yields determined by ^1^H‐NMR using 1,3,5‐trimethoxybenzene as internal standard, DMAC anhydrous (3 mL), two blue LED [440 nm, 100 %]).

A major advantage of COFs is the potential reusability of the solid photocatalyst due to easy separation from the reaction mixture. Therefore, we studied whether the Tp‐Acr framework can be recycled (Figure 5). After successful C−N coupling reaction, Tp‐Acr COF was recovered by centrifugation and reused for the same reaction. A first set of experiments in which the recovered material was washed with DMAc and lyophilized (Figure 5, blue bars) showed that Tp‐Acr can be recycled, but a significant drop in yield of **1** was observed. We hypothesized that the loss in catalytic activity can be attributed to a collapse of the pores of the COF due to removal of DMAc. Performing a solvent exchange with MeOH and hexane prior to drying improved the recyclability significantly (Figure 5, green bars). A catalytic activity of 60 % is retained after 5 cycles of catalysis. To investigate, if the nickel salt can be reused, the recycling studies were also conducted without adding NiBr_2_⋅3 H_2_O after the first run (Figure 4, red bars). A significant drop of efficiency after the first cycle was detected, meaning that addition of the nickel catalyst is needed in each cycle. Nevertheless, inductively coupled plasma optical emission spectrometry (ICP‐OES) analysis revealed that 25 % of the added nickel is deposited after the first reaction on the COF material that can drive the reaction in subsequent cycles with a yield of up to 10 %.

In order to get an understanding of the nickel species adsorbed on the material, the Tp‐Acr COF was analyzed by X‐ray photoelectron spectroscopy (XPS) after the first and fifth cycle. Ni 2p core‐level spectra (Figure S25d‐e) of the recycled material confirmed the presence of nickel(II) species with a doublet at 856 eV and 874 eV after the catalysis. Although the crystallinity of the COF is lost due to the intense light irradiation, N 1s XPS core‐level spectra show that both the aminic and pyridinic nitrogen species from the COF backbone remain unchanged during the catalysis (Figure S25a–c; S26). The structural integrity of the COF after the cross‐coupling reaction was further confirmed by IR spectroscopy (Figure S27). From additional TEM analyses it is clear that the morphology of the materials stays intact throughout the catalysis (Figure S28).

## Conclusion

In summary, we report the first successful preparation of crystalline and porous acridine‐based COFs by following an acid‐catalyzed Schiff base synthesis route using three different 1,3,5‐triformylbenzene based linkers. The acridine‐based conjugated COFs show light absorption over a broad range of the visible light spectrum. Owing to these properties, the acridine‐based COFs were applied for the first time in semi‐heterogeneous metallaphotocatalytic C−N cross‐couplings. Among the novel COFs, the fully *β*‐ketoenamine tautomerized COF, Tp‐Acr, showed a high catalytic activity both under blue and green light radiation for several aryl bromides, which is attributed to higher charge carrier separation efficiency as well as surface area. In addition, it was demonstrated that the COF could be recycled with a small drop in catalytic activity. To the best of our knowledge, this is the first example of a heterogenization of acridine photocatalysts via formation of crystalline organic frameworks.

## Conflict of interest

The authors declare no conflicts.

1

## Supporting information

As a service to our authors and readers, this journal provides supporting information supplied by the authors. Such materials are peer reviewed and may be re‐organized for online delivery, but are not copy‐edited or typeset. Technical support issues arising from supporting information (other than missing files) should be addressed to the authors.

Supporting Information

## Data Availability

The data that support the findings of this study are available from the corresponding author upon reasonable request.
