# Supplementary material for: Acridine‐Functionalized Covalent Organic Frameworks (COFs) as Photocatalysts for Metallaphotocatalytic C−N Cross‐Coupling
Source: Angew Chem Int Ed Engl. 2022 Mar 23;61(21):e202117738. doi: 10.1002/anie.202117738 (PMC9400916; doi:10.1002/anie.202117738)
Supplement: Supplementary file 1 — Supporting Information [file ANIE-61-0-s001.pdf]

Angewandte Chemie
Eine Zeitschrift der Gesellschaft Deutscher Chemiker

Supporting Information

Acridine-Functionalized Covalent Organic Frameworks (COFs) as Photocatalysts for Metallaphotocatalytic C–N Cross-Coupling

M. Traxler, S. Gisbertz, P. Pachfule, J. Schmidt, J. Roeser, S. Reischauer, J. Rabeah, B. Pieber, A. Thomas**

Table of Contents

Table of Contents 2
S1. General Remarks 3
S2. Setup for photochemical reactions 6
S3. Synthesis of organic linkers and COFs 8
S3.1 Synthesis of the organic linkers 8
S3.2 Synthesis of covalent organic frameworks (COFs) 12
S4. Structure modeling and atomic coordinates of COFs 13
S5. Characterization of COFs 20
S5.1 FT-IR results of Tp-Acr, DHTA-Acr and HTA-Acr COFs 20
S5.2 Pore size distribution for COFs 22
S5.3 SEM & TEM analysis of Tp-Acr, DHTA-Acr and HTA-Acr COFs 23
S5.4 Chemical and thermal stability of Tp-Acr, DHTA-Acr and HTA-Acr COFs 24
S5.5 UV-vis of Tp-Acr, DHTA-Acr and HTA-Acr COFs 25
S5.6 Photoluminescence measurements and fluorescence life-time of Tp-Acr, DHTA-Acr and HTA-Acr COFs 26
S5.7 EPR-results of Tp-Acr, DHTA-Acr and HTA-Acr COFs 27
S6. Synthesis and Characterization of Tp-DAA COF[6] 28
S7. Synthesis of model compound (SA-Acr) 32
S8. Photocatalytic reaction optimization 33
S8.1 General experimental procedure for screening experiments 33
S8.2 Screening of Acridine-COFs 34
S8.3 Control studies 34
S9. Photocatalysis - recycling studies 35
S9.1 Analysis of recycled Tp-Acr COF 38
S10. Scope and limitations 40
S11. References 45
S12. Author Contributions 46
S13. Copies of NMR spectra 47

S1. General Remarks

All air and moisture sensitive reactions were performed using standard Schlenk-line techniques under an atmosphere of argon. Substrates, reagents, and solvents were purchased from commercial suppliers and used without further purification. The precursors such as phloroglucinol (TCI, > 99 %), resorcinol (> 98.5 %), phenol (TCI, > 99 %), 1-chloro-3-nitrobenzene (TCI, > 99 %), 4-nitroaniline (Carl Roth, > 98.5 %), 2,6-diaminoanthraquinone (TCI, > 97 %) and salicylic aldehyde (Sigma Aldrich, 98 %) were purchased and used as received. Reactants and solvents were obtained from Sigma Aldrich ((2-biphenyl)dicyclohexylphosphine (CyJohnPhos, 97 %), 1,2-dimethoxyethane (DME, 99.5 %, anhydrous), tin (powder <150 μm, 99.5 %), mesitylene (1,3,5-trimethylbenzene, 98 %), 1,2-dichlorobenzene (*o*-DCB, 99 %, anhydrous), glycerol (99.5 %)), TCI (tris(dibenzylideneacetone)dipalladium(0) ($Pd_2(dba)_3$, > 75 %), hexamethylenetetramine (HMTA, > 99 %)), ABCR (tripotassium phosphate (K_3PO_4, 97 %), palladium (Pd/C, 10 % on activated charcoal), trifluoroacetic acid (TFA, 99.9 %), 1,4-dioxane (99.5 %)), Carl Roth (formic acid (> 98 %), hydrochloric acid (37 %), sulfuric acid (96 %)), Alphagaz (hydrogen gas (99.999 %)), Eurisotop ($CDCl_3$ (99.8 $\%_d$), DMSO-d_6 (99.8 $\%_d$)), Chemsolute (sodium hydroxide (NaOH, 99.5 %)), Fluka Analytical (sodium borohydride (> 99 %)) or Grüssing (1-butanol (*n*-BuOH, 99.5 %)) . Powder X-ray diffraction data were collected on a Bruker D8 Advance diffractometer in reflection geometry operating with a Cu K_α anode (λ = 1.54178 Å) with a working voltage at 40 kV and a current of 40 mA. Samples were ground and mounted as loose powders onto a Si sample holder. PXRD patterns were collected from 2 to 60 2θ degrees with a step size of 0.02 degrees and an exposure time of 2 seconds per step. LED lamps for photocatalytic experiments were purchased from Kessil Lightning.[1] ^{1}H-, ^{13}C-, and ^{19}F spectra were recorded on a Varian 400 spectrometer (400 MHz, Agilent), an Ascend^{TM} 400 spectrometer (400 MHz, cryoprobe, Bruker), a Varian 600 spectrometer (600 MHz, Agilent), a Bruker Avance II spectrometer (200 MHz, Bruker) and an Bruker Avance 400 spectrometer (400 MHz, Bruker) at 298 K, and are reported in ppm relative to the residual solvent peaks. Peaks are reported as: s = singlet, d = doublet, t = triplet, q = quartet, m = multiplet or unresolved, with coupling constants in Hz. ^{13}C{1H} cross polarization magic angle spinning (CP/MAS) measurements were carried out using a Bruker range Avance 400 MHz Solid State spectrometer operating at 100.6 MHz and a Bruker 4 mm double resonance probe-head operating at a spinning rate of 10 kHz. Nitrogen sorption measurements were performed at 77 K using an Autosorb-iQ-MP from Quantachrome. Prior the analysis the samples were dried and

degassed at 150 °C for 12 h. Using the N_2 adsorption isotherms, the surface areas were calculated over a pressure range 0.05-0.1 = p/p0 using Brunauer-Emmett-Teller (BET) methods. The pore size distributions were calculated from the adsorption isotherms by Quenched Solid State Functional Theory (QSDFT) using N_2 sorption data collected at 77 K. We used the carbon cylindrical pore model for analyzing the distribution. Thermogravimetric analysis (TGA) measurements were carried out under nitrogen atmosphere on a Mettler Toledo TGA 1 Stare thermal instrument with a heating rate of 10 K min^{-1}. Solid state diffuse reflectance ultraviolet-visible spectroscopy (UV-vis) spectra have been collected on a Varian Cary 300 UV-Vis Spectrophotometer. UV-vis absorption spectra of COF suspensions were collected using a Shimadzu UV-1900. Fluorescence spectra were measured using a microplate reader (SpectraMax M5, Molecular Devices). Photoluminescence lifetime was measured using the time-correlated single photon counting technique (TCSPC, FluoTime 250, fluorescence lifetime spectrometer). The Fourier transform infrared spectroscopy (FTIR) analyses of the samples were carried on Varian 640IR spectrometer equipped with an ATR cell. The scanning electron microscope (SEM) analyses of COF samples were performed on a Zeiss Gemini SEM 500 scanning electron microscope. Transmission electron microscopy (TEM) analyses were performed on a FEI Tecnai G2 20 S-TWIN transmission electron microscope (FEI Company, Eindhoven, Netherlands) equipped with a LaB_6 source at 200 kV acceleration voltage. Images were recorded with a GATAN MS794 P CCD camera. X-Ray photoelectron spectra were measured on a K-Alpha™ + X-ray Photoelectron Spectrometer System (Thermo Scientific) with Hemispheric 180° dual-focus analyzer with 128-channel detector. The X-ray monochromator used micro focused Al-Kα radiation. High resolution mass spectrometry (HR-MS) was performed on an LTQ Orbitrap XL spectrometer using electronspray ionization (ESI) or atmospheric pressure chemical ionization (APCI). Inductively coupled plasma - optical emission spectrometry (ICP-OES) was carried out using a Horiba Ultra 2 instrument equipped with a photomultiplier tube detection system. Analytical thin layer chromatography (TLC) was performed on pre-coated TLC-sheets, ALUGRAM Xtra SIL G/UV254 sheets (Macherey-Nagel) and visualized with 254 nm light or staining solutions followed by heating. Purification of final compounds was carried out by flash chromatography using Silica 60 M (0.04-0.063 mm) silica gel (Sigma Aldrich). Centrifugation was carried out using an Eppendorf 5430 centrifuge. Electron paramagnetic resonance (EPR) measurements in X-band (microwave frequency ≈ 9.8 GHz) were performed at 300 K by a Bruker EMX CW-micro spectrometer equipped with an ER 4119HS-WI high-sensitivity optical resonator with a grid in the front side. The samples were illuminated by a 300 W Xe lamp with 420 nm cut-off filter (LOT Oriel). All

the samples were measured under the same conditions (microwave power: 6.99 mW, receiver gain: 1 × 104, modulation frequency: 100 kHz, modulation amplitude: 3 G, Sweep time: 122.8 s). g values have been calculated from the resonance field B_0 and the resonance frequency ν using the resonance condition $h\nu = g\beta B_0$. The calibration of the g values was performed using DPPH (2,2-diphenyl-1- picrylhydrazyl) (g = 2.0036 ± 0.00004).

S2. Setup for photochemical reactions

Photochemical experiments involving visible light irradiation were carried out using Kessil PR160L-440 (440nm, blue light) or Kessil PR160L-525 (525 nm, green light) LED lamps with the respective power settings.[1] One or two lamps were used, depending on the required light intensity to irradiate reaction vessels located on a stirring plate (lamp-vessel distance: 4.5 cm; stirring speed: 800 rpm, Figure S2). To avoid heating of the reaction mixture, fans were used for cooling.

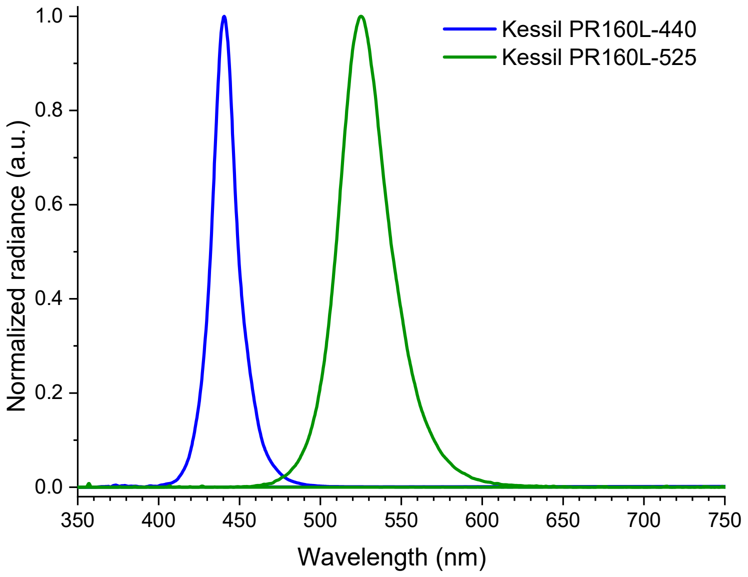

Figure S 1. Emission spectra of Kessil PR160L-400 (blue) and Kessil PR160L-525 (green).

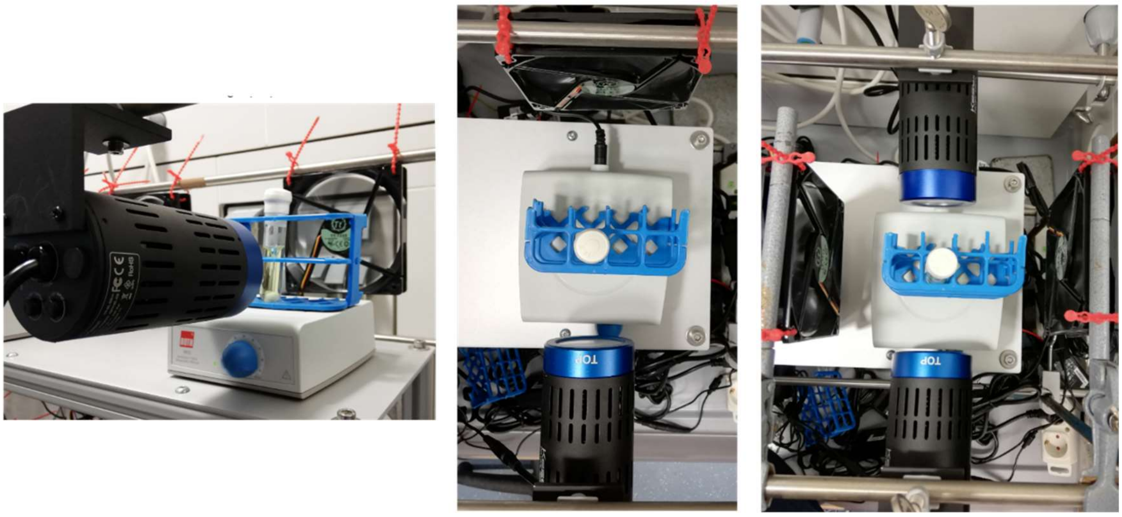

Figure S 2. Configuration of the experimental setup using one or two LED lamps.

Experiments using red light were carried out using a Kessil H160 Tuna Flora LED in “red” mode (Figure S3). Two sealed reaction vessels were placed between two lamps on a stirring plate (4.5 cm distance from each lamp). To avoid heating of the reaction mixture, a fan was used for cooling. All reactions were performed with maximum stirring speed.

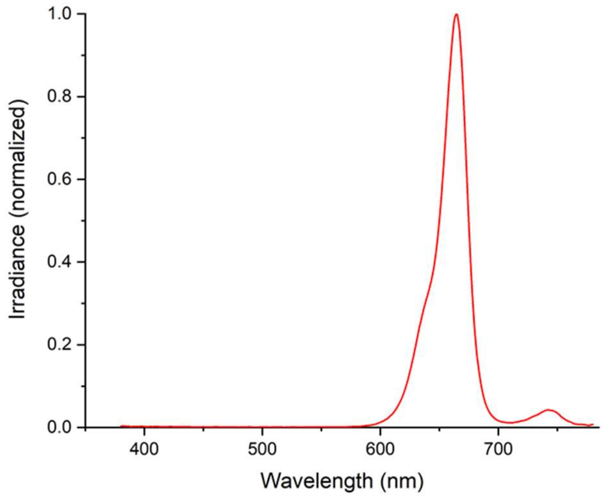

Figure S 3 . Emission spectra of the Kessil H160 Tuna Flora LED in “red” mode.

S3. Synthesis of organic linkers and COFs

S3.1 Synthesis of the organic linkers

Synthesis of 2,6-diaminoacridine:

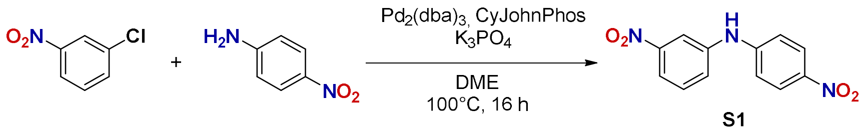

Scheme S 1. Synthesis of 3-nitro-*N*-(4-nitrophenyl)aniline (S1) via Buchwald-Hartwig coupling.

3-nitro-*N*-(4-nitrophenyl)aniline (S1): To a flame dried and three times evacuated and backfilled Schlenk flask were under argon counterflow 206 mg (0.22 mmol, 3 mol%) of $Pd_2(dba)_3$ and 80 mg (2-biphenyl)dicyclohexylphosphine (CyJohnPhos, 0.22 mmol, 3 mol%) added. 15 mL of anhydrous 1,2-dimethoxyehtane (DME) were added and the resulting suspension was stirred for 5 min at room temperature. Subsequently, 1.18 g (7.5 mmol, 1.0 eq.) of 1-chloro-3-nitrobenzene, 1.03 g (7.5 mmol, 1.0 eq.) of 4-nitroaniline and 2.23 g (10.5 mmol, 1.4 eq.) of K_3PO_4 were added and the mixture was heated to 100 °C for 24 h while stirring was maintained. After cooling to room temperature, the suspension was diluted with 300 mL of Et_2O/EtOAc (1:1), filtered through Celite and concentrated using the rotary evaporator. The crude material was purified by column chromatography on silica gel using 30 % EtOAc in cyclohexane to get the product as an orange powder (1.85 g, 95%).

^{1}H NMR (400 MHz, DMSO-d_6) δ 9.67 (s, 1H), 8.16 (d, J = 9.2 Hz, 2H), 7.86 (ddd, J = 7.8, 2.2, 1.2 Hz, 1H), 7.68 (ddd, J = 8.1, 2.1, 1.2 Hz, 1H), 7.63 (t, J = 8.0 Hz, 1H), 7.21 (d, J = 9.3 Hz, 2H). ^{13}C NMR (101 MHz, DMSO-d_6) δ 149.13, 148.66, 141.91, 139.37, 130.90, 126.10, 125.40, 116.86, 114.83, 113.41.

These data are in full agreement with those previously published in the literature.[2]

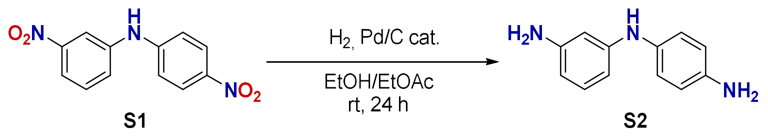

Scheme S 2. Synthesis of 3-(4-aminoanilino)aniline (S2) from 3-nitro-*N*-(4-nitrophenyl)aniline (S1).

3-(4-aminoanilino)aniline (S2): A suspension of 1.66 g (6.4 mmol) of 3-nitro-*N*-(4-nitrophenyl)aniline (**S1**), 332 mg (0.31 mmol, 4.9 mol%) of Pd/C (10 wt% Pd), 200 mL EtOH

and 100 mL EtOAc were degassed for 15 min using an argon purge. Subsequently, the suspension was degassed for 10 min using and H_2 purge and thereafter stirred for 24 h under hydrogen atmosphere. The catalyst was removed by filtration through Celite and the solvent was concentrated under vacuum and dried at 65 °C under high vacuum to give a dark brown solid (1.23 g, 96 %).

^{1}H NMR (200 MHz, DMSO-d_6) δ 7.11 (s, 1H), 6.83 – 6.68 (m, 3H), 6.51 (d, *J* = 8.6 Hz, 2H), 6.06 (t, *J* = 2.0 Hz, 1H), 5.99 (ddd, *J* = 7.9, 2.1, 0.8 Hz, 1H), 5.88 (ddd, *J* = 7.8, 2.0, 0.9 Hz, 1H), 4.74 (bs, 4H). ^{13}C NMR (50 MHz, DMSO-d_6) δ 149.20, 147.28, 143.21, 132.35, 129.14, 122.39, 114.69, 104.20, 103.03, 99.56.

These data are in full agreement with those previously published in the literature.[2]

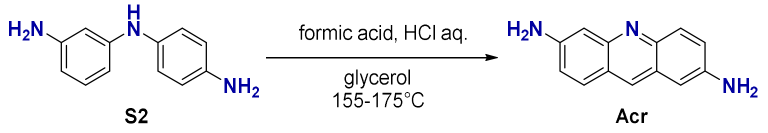

Scheme S 3. Synthesis of 2,6-diaminoacridine (Acr) from 3-(4-aminoanilino)aniline (S2).

2,6-diaminoacridine (Acr): To 1.23 g (6.1 mmol, 1.0 eq.) of 3-(4-aminoanilino)aniline (**S2**) were 4 mL glycerol, 235 µl (6.1 mmol, 1.0 eq.) of formic acid and 660 µl (7.9 mmol, 1.3 eq.) of 37 % aqueous HCl solution added. The reaction mixture was heated to 155 °C during 30 min, kept at this temperature for 30 min before heating it to 175 °C for another 30 min. After cooling to room temperature 2.5 mL of aqueous sulfuric acid (30 $\%_{w/v}$) were added and the reaction was heated to 95 °C for 10 min. Subsequently, the reaction was diluted to 25 mL with H_2O and the reaction mixture was kept at 0 °C for 1 h. The precipitated acid sulfate of the compound was filtered off, washed with water (40 mL) and Et_2O (60 mL). The dark red powder was boiled in 10 mL of aqueous NaOH (1 M) to precipitate the 2,6-diaminoacridine. The dark brown compound was filtered and washed with ice cold water (60 mL) and cold Et_2O (10 mL) and dried under vacuum at 65 °C (407 mg, 32 %).

^{1}H NMR (400 MHz, DMSO-d_6) δ 8.35 – 8.24 (m, 1H), 7.67 (dd, *J* = 9.0, 2.9 Hz, 2H), 7.22 (dd, *J* = 9.2, 2.3 Hz, 1H), 6.99 (dd, *J* = 9.0, 2.0 Hz, 1H), 6.84 – 6.82 (m, 1H), 6.82 – 6.80 (m, 1H), 5.83 – 5.78 (m, 1H), 5.43 – 5.39 (m, 1H). ^{13}C NMR (101 MHz, DMSO-d_6) δ 148.54, 148.07, 144.44, 144.07, 130.64, 128.51, 128.23, 125.84, 124.52, 121.45, 120.55, 103.65, 103.48. HR-ESI-MS [M+H$^+$] (m/z)): 210.1028 (th.: 210.1026).

Synthesis of aldehyde linkers:

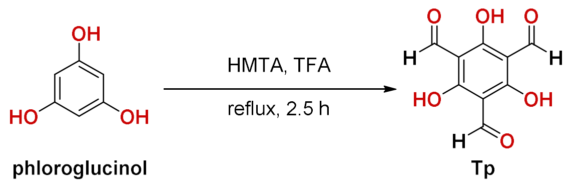

Scheme S 4. Synthesis of 1,3,5-triformylphloroglucino (Tp) from phloroglucinol.

1,3,5-triformylphloroglucinol (Tp): To 10.0 g (80 mmol, 1.0 eq.) of phloroglucinol and 25.0 g (179 mmol, 2.2 eq.) of hexamethylenetetramine (HMTA) were 150 mL trifluoroacetic acid (TFA) added slowly under argon atmosphere at 0 °C. After complete addition, the suspension was heated at 100 °C for 2.5 h. The reaction mixture was cooled to around 50 °C and 240 mL of 3 M HCl were added, and the solution was heated at 100 ºC for 1 h. After cooling to room temperature, the solution was filtered through Celite, extracted with 3x 200 mL dichloromethane, dried over magnesium sulfate, and filtered. Rotary evaporation of the solution afforded of an off-white powder. A pure sample was obtained by washing the solid sample with 10 mL of cold EtOH followed by sublimation under reduced pressure (2.76 g, 16 %).

^{1}H NMR (200 MHz, $CDCl_3$) δ 14.11 (s, 1H), 10.14 (s, 1H). ^{13}C NMR (50 MHz, $CDCl_3$) δ 192.17, 173.71, 103.05.

These data are in full agreement with those previously published in the literature.[3]

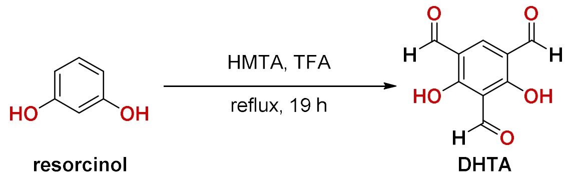

Scheme S 5. Synthesis of 2,4-dihydroxy-1,3,5-triformylcarbaldehyde (DHTA) from resorcinol.

2,4-dihydroxy-1,3,5-triformylcarbaldehyde (DHTA): To 3.6 g (33 mmol, 1.0 eq.) of resorcinol and 10.0 g (71 mmol, 2.2 eq.) of hexamethylenetetramine (HMTA) were 35 mL trifluoroacetic acid added slowly under argon atmosphere at 0 °C. After complete addition, the suspension was heated at 130 °C for 16 h and afterwards 3 h at 150 °C. The reaction mixture was cooled to around 100 °C and 55 mL of 3 M HCl were added, and the solution was heated at 105 ºC for 30 min. After cooling to room temperature, the solution was filtered through Celite, extracted with 3x 50 mL dichloromethane, dried over magnesium sulfate, and filtered. Rotary evaporation of the solution afforded of an off-white powder. A pure sample was

obtained by washing the solid sample with 10 mL of cold EtOH followed by sublimation under reduced pressure (1.37 g, 21 %).

^{1}H NMR (400 MHz, DMSO-d_6) δ 10.26 (s, 1H), 10.09 (s, 2H), 8.40 (s, 1H). ^{13}C NMR (101 MHz, DMSO-d_6) δ 193.93, 189.97, 169.97, 140.66, 115.80, 109.95.

These data are in full agreement with those previously published in the literature.[4]

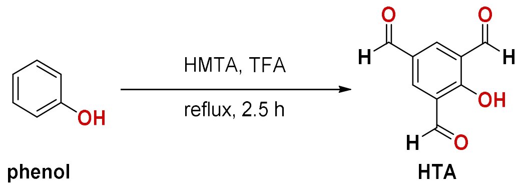

Scheme S 6. Synthesis of 2-hydroxy-1,3,5-triformylcarbaldehyde (HTA) from phenol.

2-hydroxy-1,3,5-triformylcarbaldehyde (HTA): To 3.5 g (36.7 mmol, 1.0 eq.) of phenol and 10.1 g (71.3 mmol, 2.0 eq.) of hexamethylenetetramine (HMTA) were 50 mL trifluoroacetic acid added slowly under argon atmosphere at 0 °C. After complete addition, the suspension was heated at 120 °C for 20 h and afterwards 30 min at 150 °C. The reaction mixture was cooled to around 120 °C and 50 mL of 3 M HCl were added, and the solution was heated at 120 ºC for 30 min. While cooling to room temperature a yellow precipitate was formed, which was filtered, washed with 20 mL of cold EtOH and dried under vacuo to give an off-white solid (3.64 g, 56%). Pure sample was obtained by sublimation of the crude material under reduced pressure.

^{1}H NMR (200 MHz, DMSO-d_6) δ 10.30 (s, 2H), 9.99 (s, 1H), 8.52 (s, 2H). ^{1}H NMR (200 MHz, $CDCl_3$) δ 12.16 (s, 1H), 10.32 (s, 2H), 10.01 (s, 1H), 8.51 (s, 2H). ^{13}C NMR (50 MHz, DMSO-d_6) δ 191.65, 190.72, 165.88, 137.31, 128.30, 124.09.

These data are in full agreement with those previously published in the literature.[5]

S3.2 Synthesis of covalent organic frameworks (COFs)

Tp-Acr COF: A Pyrex tube (o.d. × i.d. = 15 × 10 mm^2 and length 15 cm) is charged with triformylphloroglucinol (**Tp**) (21 mg, 0.1 mmol), 2,6-diaminoacridine (**Acr**) (31.5 mg, 0.15 mmol), 1.5 mL of mesitylene, 1.5 mL of dioxane and 0.5 mL of 6 M aqueous acetic acid. This mixture was sonicated for 15 minutes in order to get a homogenous dispersion. The tube was then flash frozen at 77 K (liquid N_2 bath) and degassed by three freeze-pump-thaw cycles. The tube was sealed off and then heated at 120 °C for 3 days. A dark red colored precipitate was collected by filtration, washed with anhydrous acetone, methanol and cyclohexane before Soxhlet extraction with methanol. The powder collected was then solvent exchanged twice, with a cyclohexane and then dried at 120 °C to give a dark red colored powder (46 mg, 98 %).

DHTA-Acr COF: The synthesis of **DHTA-Acr COF** was carried out by utilizing the same protocol with a mixture of 2,4-dihydroxybenzene-1,3,5-tricarbaldehyde (**DHTA**) (19.4 mg, 0.1 mmol), 2,6-diaminoacridine (**Acr**) (31.5 mg, 0.15 mmol), 1.5 mL of mesitylene, 1.5 mL of dioxane and 0.5 mL of 6 M aqueous acetic acid. This mixture was sonicated for 15 minutes in order to get a homogenous dispersion. The tube was then flash frozen at 77 K (liquid N2 bath) and degassed by three freeze-pump-thaw cycles. The tube was sealed off and then heated at 120 °C for 3 days. A dark red colored precipitate was collected by filtration, washed with anhydrous acetone, methanol and cyclohexane before Soxhlet extraction with methanol. The powder collected was then solvent exchanged twice, with a cyclohexane and then dried at 120 °C to give a dark red colored powder (46 mg, 99 %).

HTA-Acr COF: The synthesis of **HTA-Acr COF** was carried out by utilizing the same protocol with a mixture of 2-hydroxybenzene-1,3,5-tricarbaldehyde (**HTA**) (17.8 mg, 0.1 mmol), 2,6-diaminoacridine (**Acr**) (31.5 mg, 0.15 mmol), 1.5 mL of *n*-BuOH, 1.5 mL of anhydrous *o*-DCB and 0.5 mL of 6 M aqueous acetic acid. This mixture was sonicated for 15 minutes in order to get a homogenous dispersion. The tube was then flash frozen at 77 K (liquid N2 bath) and degassed by three freeze-pump-thaw cycles. The tube was sealed off and then heated at 120 °C for 3 days. A dark red colored precipitate was collected by filtration, washed with anhydrous acetone, methanol and cyclohexane before Soxhlet extraction with methanol. The powder collected was then solvent exchanged twice, with a cyclohexane and then dried at 120 °C to give a dark red colored powder (37 mg, 84 %).

S4. Structure modeling and atomic coordinates of COFs

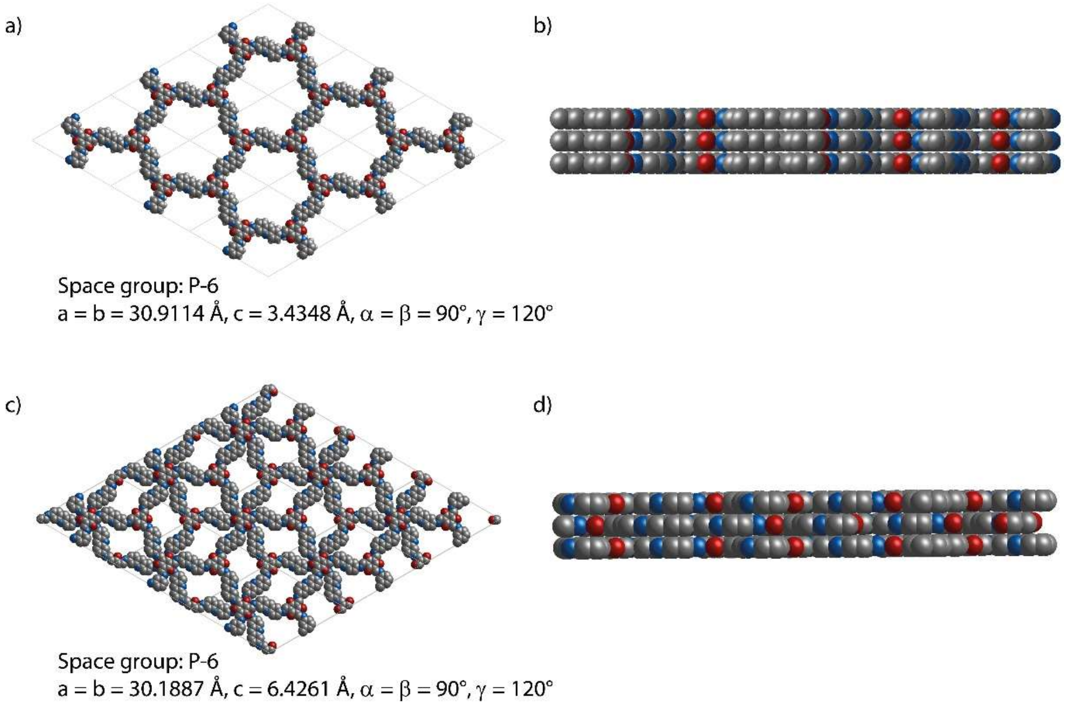

Figure S 4. Computationally determined structures of Tp-Acr. a) The theoretical structure of Tp-Acr with eclipsed (AA) stacking arrangement. b) Side view of Tp-Acr structure with eclipsed (AA) stacking arrangement. c) The theoretical structure of Tp-Acr with staggered (AB) stacking arrangement. d) Side view of Tp-Acr structure with staggered (AB) stacking arrangement.

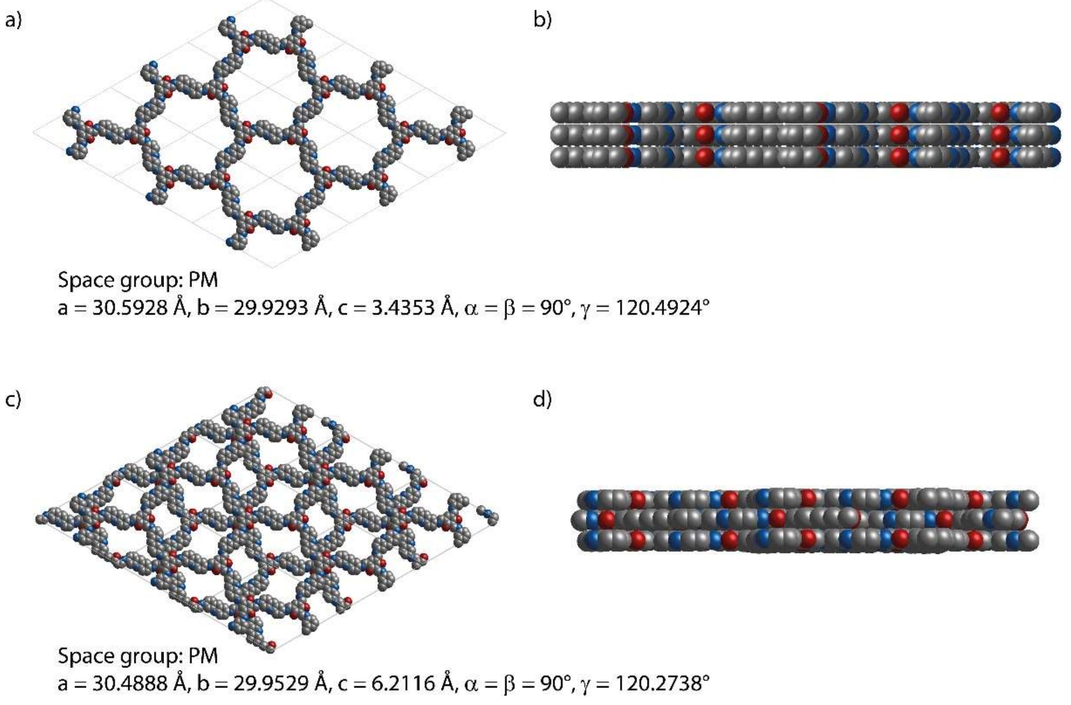

Figure S 5. Computationally determined structures of DHTA-Acr. a) The theoretical structure of DHTA-Acr with eclipsed (AA) stacking arrangement. b) Side view of DHTA-Acr structure with eclipsed (AA) stacking arrangement. c) The theoretical structure of DHTA-Acr with staggered (AB) stacking arrangement. d) Side view of DHTA-Acr structure with staggered (AB) stacking arrangement.

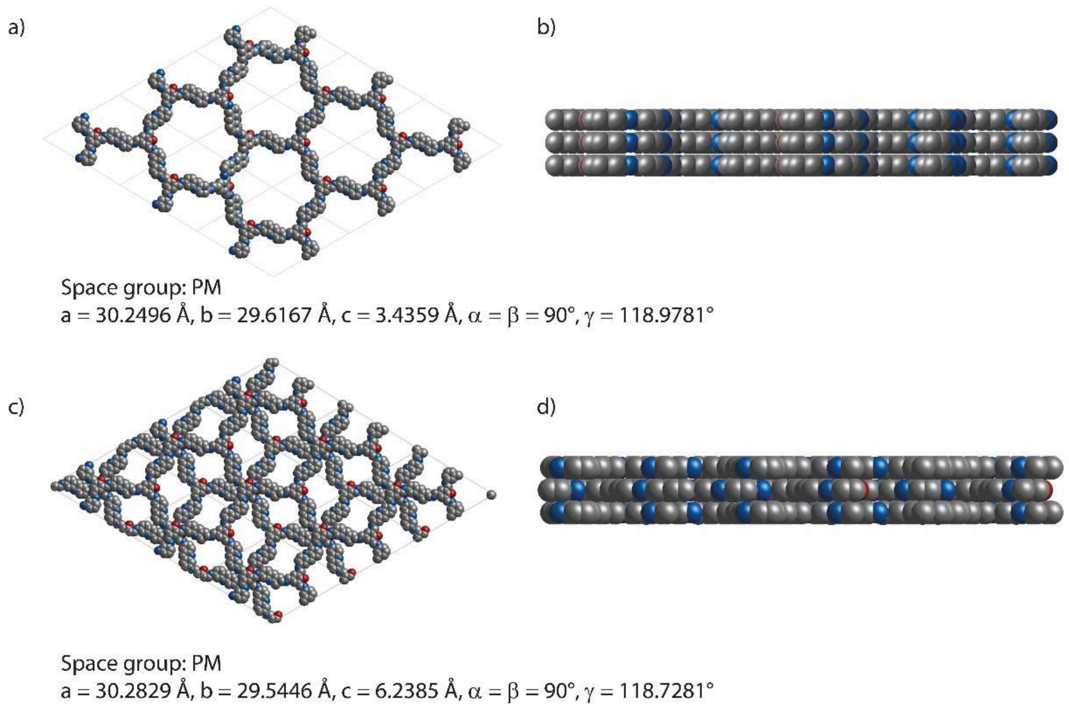

Figure S 6. Computationally determined structures of HTA-Acr. a) The theoretical structure of HTA-Acr with eclipsed (AA) stacking arrangement. b) Side view of HTA-Acr structure with eclipsed (AA) stacking arrangement. c) The theoretical structure of HTA-Acr with staggered (AB) stacking arrangement. d) Side view of HTA-Acr structure with staggered (AB) stacking arrangement.

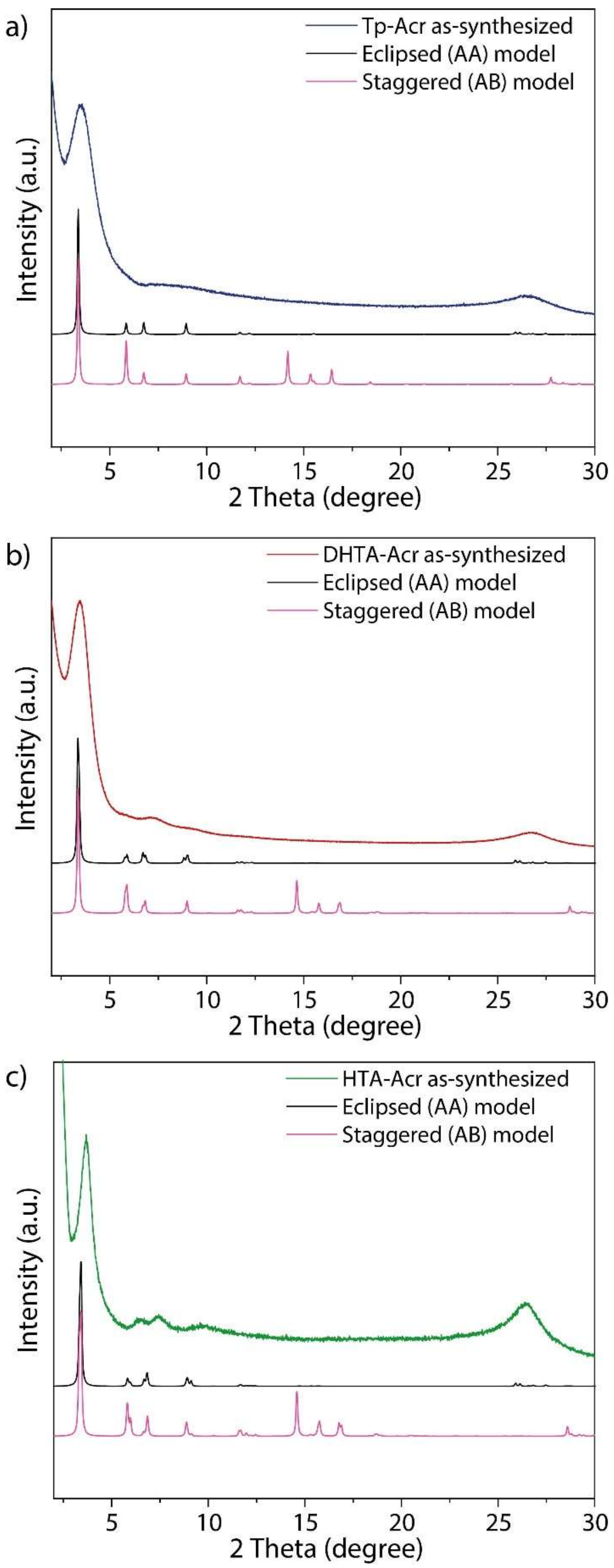

Figure S 7. Comparison between simulated and experimental PXRD patterns for a) Tp-Acr, b) DHTA-Acr and HTA-Acr COFs, showing a good match between the experimental diffractogram and the eclipsed stacking model (AA).

Table S 1. Fractional atomic coordinates for Tp-Acr COF.

**Tp-Acr-COF**<br>Space group symmetry: P-6 (174)<br>a = b = 30.9114 Å; c = 3.4348 Å<br>α = β = 90°; γ = 120°				
**Atom name**	**Atom**	**x**	**y**	**z**
H1	H	0.44644	0.40004	0
H2	H	0.46699	0.59166	0
H3	H	0.36561	0.40202	0
H4	H	0.32101	0.45088	0
C5	C	0.54847	0.44164	0
C6	C	0.60253	0.46881	0
C7	C	0.62804	0.52263	0
C8	C	0.6009	0.54886	0
H9	H	0.5268	0.40048	0
H10	H	0.62148	0.59027	0
H11	H	0.66942	0.54455	0
C12	C	0.44374	0.5506	0
C13	C	0.38973	0.52526	0
C14	C	0.36241	0.47142	0
C15	C	0.38778	0.44341	0
O16	O	0.27439	0.56395	0
N17	N	0.36115	0.55203	0
C18	C	0.30132	0.61027	0
C19	C	0.35822	0.63396	0
C20	C	0.38373	0.60706	0
C21	C	0.46926	0.52263	0
C22	C	0.44121	0.46874	0
N23	N	0.52124	0.5475	0
H24	H	0.42476	0.62902	0
H25	H	0.32187	0.52808	0
O26	O	0.72219	0.43631	0
N27	N	0.63293	0.44395	0
C28	C	0.69679	0.38986	0
C29	C	0.63972	0.36411	0
C30	C	0.61239	0.38919	0
C31	C	0.52111	0.46795	0
C32	C	0.54758	0.52186	0
C33	C	0.46752	0.44151	0
H34	H	0.57146	0.36585	0
H35	H	0.67198	0.46913	0

Table S 2. Fractional atomic coordinates for DHTA-Acr COF.

**DHTA-Acr-COF**<br>Space group symmetry: PM (6)<br>a = 30.5928; b = 29.9293 Å; c = 3.4353 Å<br>α = β = 90°; γ = 120.4924°				
**Atom name**	**Atom**	**x**	**y**	**z**
H1	H	0.44528	0.39443	0
H2	H	0.59584	0.04762	0
H3	H	0.96004	0.5596	0
H4	H	0.46471	0.58747	0
H5	H	0.36468	0.39517	0
H6	H	0.32011	0.44378	0
C7	C	0.54672	0.43811	0
C8	C	0.6004	0.46656	0
C9	C	0.62524	0.52108	0
C10	C	0.59794	0.54682	0
H11	H	0.5256	0.39633	0
H12	H	0.61804	0.5888	0
H13	H	0.66627	0.54396	0
C14	C	0.44169	0.54581	0
C15	C	0.38813	0.5196	0

C16	C	0.36118	0.46501	0
C17	C	0.38653	0.43715	0
O18	O	0.27334	0.55829	0
N19	N	0.35979	0.54633	0
C20	C	0.30106	0.60517	0
C21	C	0.35718	0.62886	0
C22	C	0.38241	0.60207	0
C23	C	0.46716	0.51805	0
C24	C	0.43958	0.46342	0
N25	N	0.5187	0.54393	0
H26	H	0.4231	0.62478	0
H27	H	0.32074	0.52207	0
O28	O	0.72453	0.4418	0
N29	N	0.63125	0.44252	0
C30	C	0.70007	0.39464	0
C31	C	0.64319	0.36637	0
C32	C	0.61315	0.39038	0
C33	C	0.51917	0.46399	0
C34	C	0.5451	0.51864	0
C35	C	0.46598	0.43647	0
H36	H	0.57265	0.36454	0
H37	H	0.66988	0.46875	0
H38	H	0.40194	0.87345	0
H39	H	0.59001	0.96127	0
H40	H	0.54012	0.86675	0
C41	C	0.55801	0.11073	0
C42	C	0.53324	0.13979	0
C43	C	0.47982	0.11263	0
C44	C	0.45189	0.05835	0
H45	H	0.5987	0.12888	0
H46	H	0.41085	0.03834	0
H47	H	0.45962	0.13364	0
C48	C	0.44269	0.89145	0
C49	C	0.467	0.86188	0
C50	C	0.52037	0.88822	0
C51	C	0.549	0.94214	0
O52	O	0.4337	0.70739	0
N53	N	0.44008	0.8058	0
C54	C	0.38769	0.68662	0
C55	C	0.36257	0.71853	0
C56	C	0.38863	0.77386	0
C57	C	0.47155	0.94584	0
C58	C	0.52485	0.97104	0
N59	N	0.44826	0.9743	0
H60	H	0.36531	0.79138	0
H61	H	0.4639	0.79072	0
H62	H	0.57559	0.29257	0
N63	N	0.56037	0.19604	0
C64	C	0.61638	0.31174	0
C65	C	0.63976	0.28168	0
C66	C	0.61202	0.22662	0
C67	C	0.53009	0.05646	0
C68	C	0.4768	0.03009	0
C69	C	0.55476	0.02804	0
H70	H	0.63436	0.20826	0
H71	H	0.53768	0.2127	0
H72	H	0.12572	0.53095	0
H73	H	0.04725	0.64078	0
H74	H	0.14005	0.68259	0
C75	C	0.89605	0.45624	0
C76	C	0.86758	0.40143	0
C77	C	0.89373	0.37401	0
C78	C	0.94653	0.39995	0
H79	H	0.87788	0.47909	0
H80	H	0.96579	0.37799	0
H81	H	0.873	0.33203	0
C82	C	0.11033	0.55629	0
C83	C	0.14131	0.61092	0
C84	C	0.11748	0.64067	0
C85	C	0.06457	0.61695	0
H86	H	0.29235	0.71675	0
N87	N	0.19627	0.63752	0

C88	C	0.30919	0.69272	0
C89	C	0.27767	0.6384	0
C90	C	0.22389	0.61323	0
C91	C	0.05753	0.5326	0
C92	C	0.03438	0.56288	0
N93	N	0.02871	0.48017	0
H94	H	0.20402	0.57162	0
H95	H	0.21443	0.67778	0
O96	O	0.71564	0.28134	0
N97	N	0.81276	0.37191	0
C98	C	0.69617	0.30807	0
C99	C	0.72796	0.36576	0
C100	C	0.77986	0.39347	0
C101	C	0.94937	0.48246	0
C102	C	0.9744	0.4539	0
C103	C	0.97836	0.53669	0
H104	H	0.79782	0.43508	0
H105	H	0.79792	0.3322	0

Table S 3. Fractional atomic coordinates for HTA-Acr COF.

**HTA-Acr-COF**<br>Space group symmetry: PM (6)<br>a = 30.2496; b = 29.6167 Å; c = 3.4359 Å<br>α = β = 90°; γ = 118.9781°				
**Atom name**	**Atom**	**x**	**y**	**z**
H1	H	0.45098	0.39971	0
H2	H	0.59571	0.04	0
H3	H	0.95459	0.56059	0
H4	H	0.46949	0.59409	0
H5	H	0.37111	0.4026	0
H6	H	0.32628	0.45239	0
C7	C	0.55152	0.44029	0
C8	C	0.60495	0.46675	0
C9	C	0.63017	0.52109	0
C10	C	0.60328	0.54843	0
H11	H	0.52994	0.39878	0
H12	H	0.62361	0.59022	0
H13	H	0.67106	0.5425	0
C14	C	0.44709	0.5526	0
C15	C	0.39364	0.52724	0
C16	C	0.36719	0.47292	0
C17	C	0.39268	0.44436	0
O18	O	0.27272	0.56071	0
N19	N	0.36451	0.55417	0
C20	C	0.29871	0.60746	0
C21	C	0.355	0.63263	0
C22	C	0.38396	0.60647	0
C23	C	0.47283	0.52397	0
C24	C	0.44544	0.46957	0
N25	N	0.52427	0.54853	0
H26	H	0.42446	0.63052	0
H27	H	0.32581	0.52992	0
H28	H	0.7183	0.42938	0
N29	N	0.63484	0.44053	0
C30	C	0.69738	0.38782	0
C31	C	0.644	0.36206	0
C32	C	0.61525	0.38817	0
C33	C	0.52452	0.46762	0
C34	C	0.55053	0.522	0
C35	C	0.47164	0.44156	0
H36	H	0.57486	0.36396	0
H37	H	0.67377	0.46457	0
H38	H	0.40098	0.87292	0
H39	H	0.5879	0.95154	0
H40	H	0.53624	0.85749	0
C41	C	0.55942	0.10646	0
C42	C	0.53513	0.13698	0

C43	C	0.48195	0.11177	0
C44	C	0.45366	0.05794	0
H45	H	0.59998	0.12342	0
H46	H	0.41279	0.03942	0
H47	H	0.4622	0.13401	0
C48	C	0.44144	0.88874	0
C49	C	0.46441	0.85698	0
C50	C	0.51752	0.88073	0
C51	C	0.54711	0.93434	0
H52	H	0.42223	0.70463	0
N53	N	0.4361	0.80117	0
C54	C	0.38166	0.68687	0
C55	C	0.35758	0.71758	0
C56	C	0.38464	0.77231	0
C57	C	0.47115	0.94259	0
C58	C	0.52415	0.9654	0
N59	N	0.44879	0.9728	0
H60	H	0.36233	0.79173	0
H61	H	0.45827	0.7832	0
H62	H	0.57618	0.2898	0
N63	N	0.56237	0.19289	0
C64	C	0.61681	0.3077	0
C65	C	0.64052	0.27707	0
C66	C	0.61378	0.22236	0
C67	C	0.53103	0.05243	0
C68	C	0.47793	0.02815	0
C69	C	0.55483	0.02213	0
H70	H	0.63646	0.20349	0
H71	H	0.53976	0.21042	0
H72	H	0.12359	0.53314	0
H73	H	0.04035	0.64186	0
H74	H	0.13304	0.68386	0
C75	C	0.89343	0.45747	0
C76	C	0.86667	0.40285	0
C77	C	0.89438	0.376	0
C78	C	0.94696	0.40221	0
H79	H	0.87401	0.47988	0
H80	H	0.9674	0.38066	0
H81	H	0.87503	0.33422	0
C82	C	0.1072	0.5583	0
C83	C	0.13675	0.61268	0
C84	C	0.11158	0.64212	0
C85	C	0.05872	0.61829	0
H86	H	0.28751	0.71855	0
N87	N	0.19154	0.63921	0
C88	C	0.30438	0.69375	0
C89	C	0.27358	0.6399	0
C90	C	0.22009	0.61492	0
C91	C	0.05453	0.53453	0
C92	C	0.02997	0.56443	0
N93	N	0.02722	0.48235	0
H94	H	0.20136	0.57351	0
H95	H	0.20896	0.67933	0
O96	O	0.71789	0.27792	0
N97	N	0.81212	0.37275	0
C98	C	0.69661	0.3038	0
C99	C	0.72559	0.36149	0
C100	C	0.77921	0.39193	0
C101	C	0.94661	0.48398	0
C102	C	0.97312	0.45589	0
C103	C	0.97404	0.53801	0
H104	H	0.79433	0.43324	0
H105	H	0.79884	0.33333	0

S5. Characterization of COFs

S5.1 FT-IR results of Tp-Acr, DHTA-Acr and HTA-Acr COFs

Fourier transform infrared (FT-IR) spectra show the disappearance of the vibration of the amino group of the 2,6-diaminoacridine linker at around 3400 cm^{-1}, and of the stretching vibration of C=O groups of the aldehyde linker (1690-1640 cm^{-1}). The bands of the newly formed C=O and C=C bonds were merged into one single peak at 1580 cm^{-1} for the Tp-Acr COF, at 1552 cm^{-1} for the DHTA-Acr COF and 1578 cm^{-1} for the HTA-Acr COF, respectively. Furthermore, the C-N stretching vibration appeared around 1270 cm^{-1} for all COFs. Distinctive spectral bands of the acridine linker at ~800 cm^{-1} can be also found in the acridine COFs.

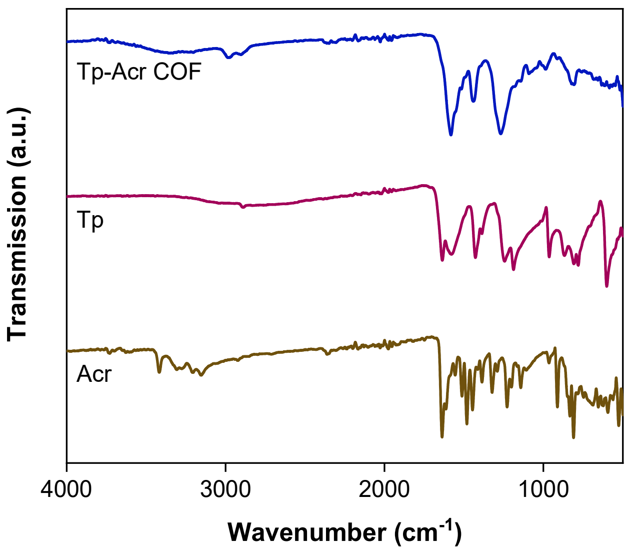

Figure S 8. FT-IR analyses of Tp-Acr COF in comparison with the corresponding aldehyde (Tp) and amine (Acr) showing the formation of the framework structure.

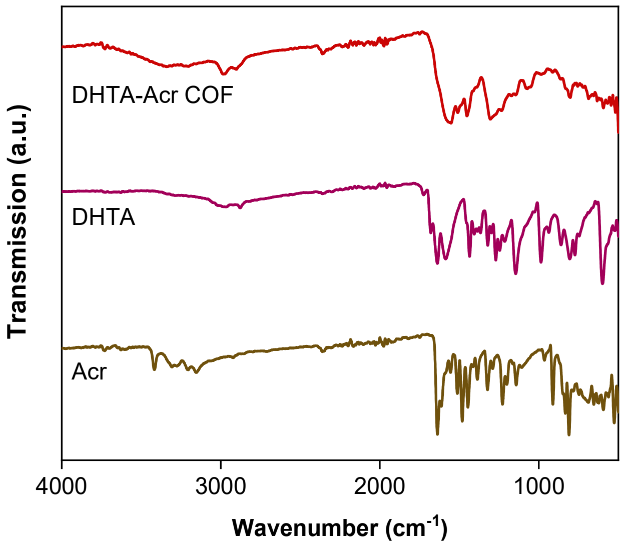

Figure S 9. FT-IR analyses of DHTA-Acr COF in comparison with the corresponding aldehyde (DHTA) and amine (Acr) showing the formation of the framework structure.

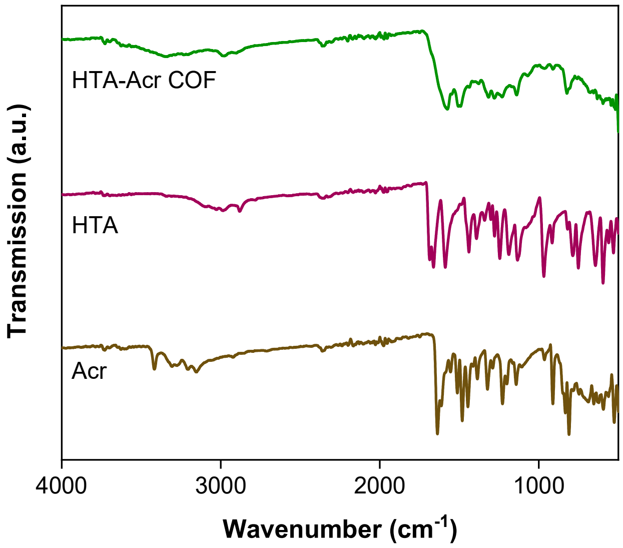

Figure S 10. FT-IR analyses of HTA-Acr COF in comparison with the corresponding aldehyde (HTA) and amine (Acr) showing the formation of the framework structure.

S5.2 Pore size distribution for COFs

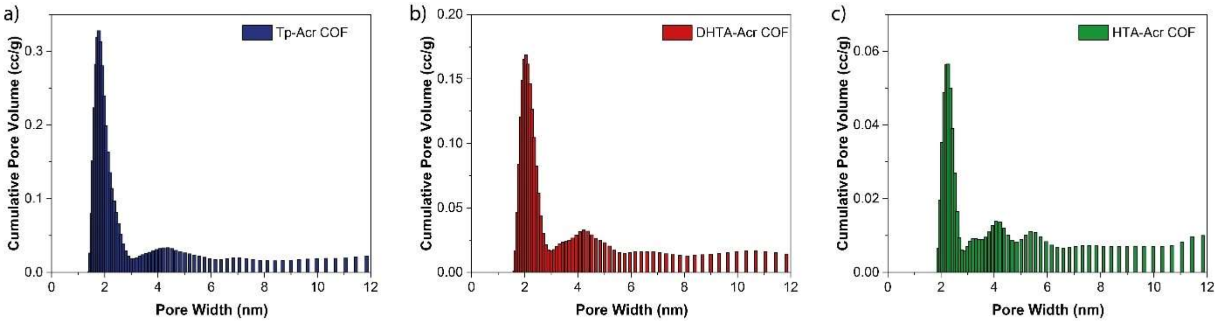

Figure S 11. Pore size distribution of (a) Tp-Acr, (b) DHTA-Acr and HTA-Acr COFs, showing the pores distribution close to the ideal pore size calculated from simulated structures.

Tp-Acr COF:

Method = Quenched Solid State Functional Theory (QSDFT)

Model = N_2 at 77 K on carbon cylindrical pore (fitting error = 1.6%)

DHTA-Acr COF:

Method = Quenched Solid State Functional Theory (QSDFT)

Model = N_2 at 77 K on carbon cylindrical pore (fitting error = 3.0%)

HTA-Acr COF:

Method = Quenched Solid State Functional Theory (QSDFT)

Model = N_2 at 77 K on carbon cylindrical pore (fitting error = 3.1%)

S5.3 SEM & TEM analysis of Tp-Acr, DHTA-Acr and HTA-Acr COFs

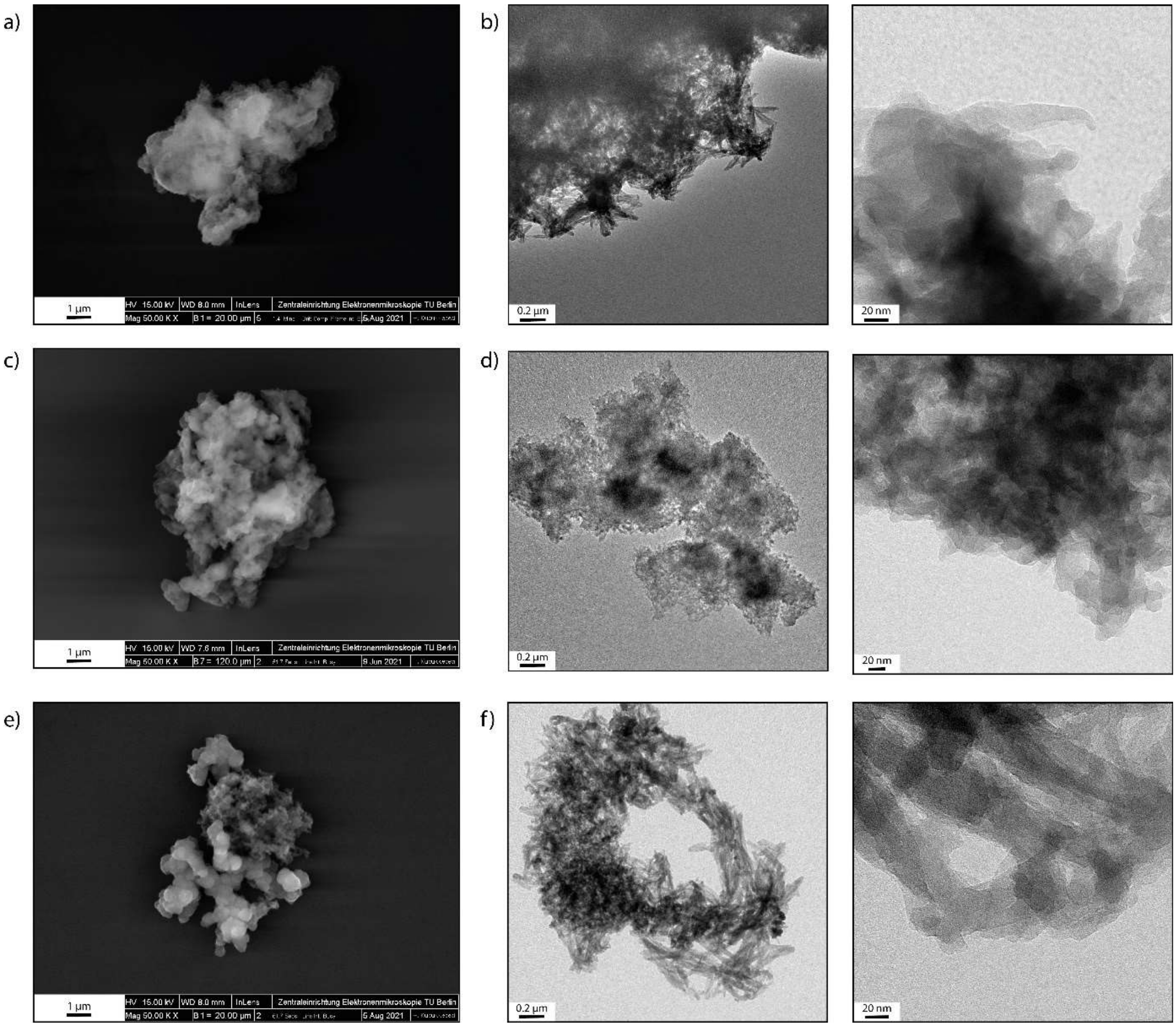

Figure S 12. SEM and TEM analyses of Tp-Acr, DHTA-Acr and HTA-Acr. (a, c, e) SEM images of Tp-Acr, DHTA-Acr and HTA-Acr COFs showing the flower like structures, respectively. (b, d, f) TEM images of Tp-Acr, DHTA-Acr and HTA-Acr COFs showing the morphology and the layered structure of the COF matrix, respectively.

S5.4 Chemical and thermal stability of Tp-Acr, DHTA-Acr and HTA-Acr COFs

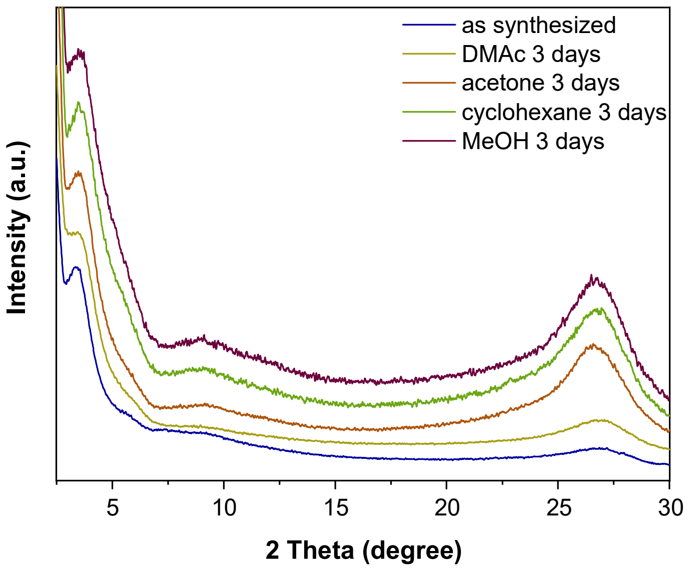

Figure S 13. Chemical stability of Tp-Acr. The PXRD profiles of the COF sample treated for 3 days in different solvents compared to the as-synthesized COF.

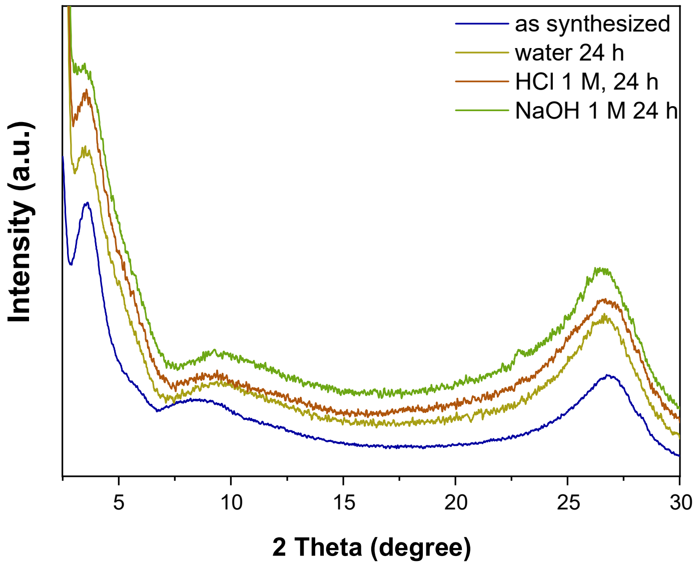

Figure S 14. Chemical stability of Tp-Acr. The PXRD profiles of the COF sample treated for 24 h in different aqueous neutral, acidic and alkaline conditions compared to the as-synthesized COF.

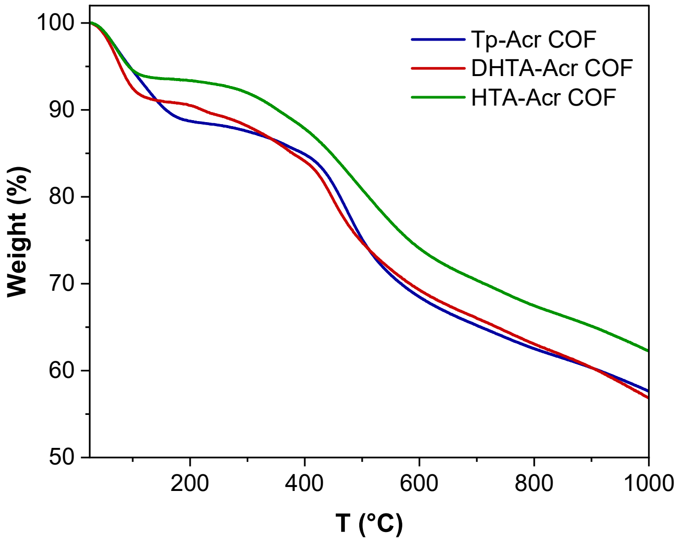

Figure S 15. Thermogravimetric analyses (TGA) for Tp-Acr, DHTA-Acr and HTA-Acr COFs, showing the termal stability of the COFs below 300 °C, under nitrogen atmosphere.

S5.5 UV-vis of Tp-Acr, DHTA-Acr and HTA-Acr COFs

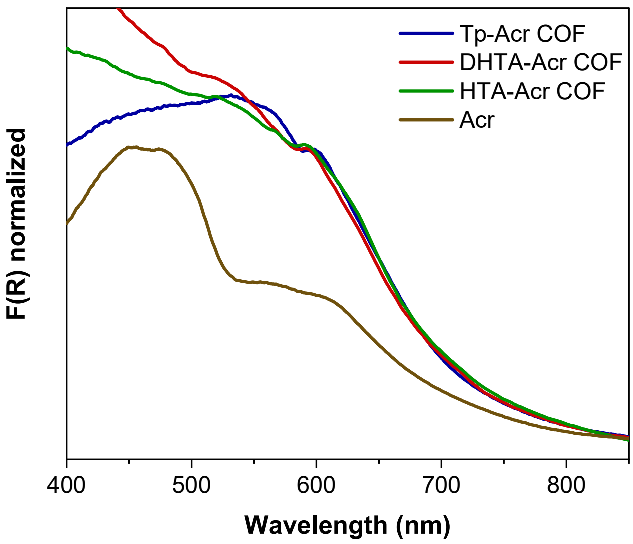

Figure S 16. UV-vis diffuse reflectance spectroscopy analysis of Tp-Acr, DHTA-Acr and HTA-Acr in comparison to 2,6-diaminoacridine (Acr).

S5.6 Photoluminescence measurements and fluorescence life-time of Tp-Acr, DHTA-Acr and HTA-Acr COFs

Preparation of the samples for luminescence studies:

0.5 mg of COF was dispersed in 10 ml dimethylacetamide by sonicating for 5 minutes at 40 °C. Prior the UV-vis and PL measurements the bigger particles were let to sediment to obtain a nearly clear solution. The clear COF dispersed solutions were used for the PL and steady-state time-resolved fluorescence decay measurements.

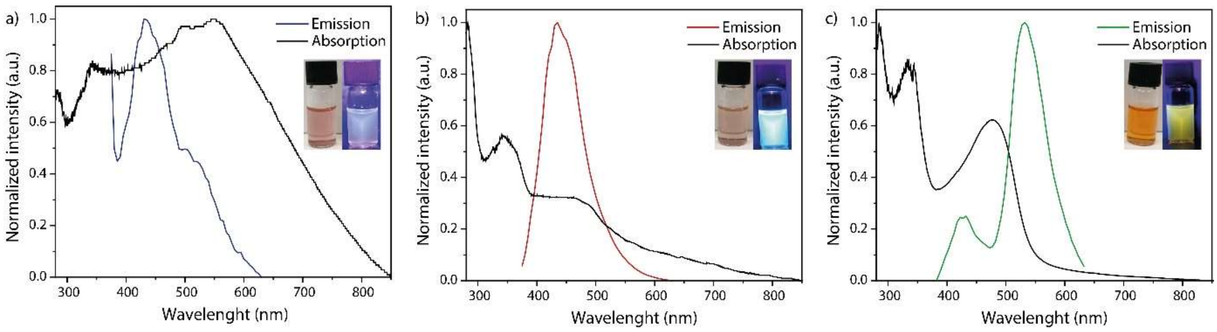

Figure S 17.Steady-state absorption and emission profiles of dispersed a) Tp-Acr, b) DHTA-Acr and c) HTA-Acr in dimethylacetamide. For the fluorescence measurements the samples were excited at a wavelength of 360 nm. Optical images at ambient light and upon excitation under a 365 nm UV-lamp is shown in every case.

Table S 4. Life-time decay components and amplified average life-time

COF	$\lambda_{exc.}$ (nm)	$\lambda_{col.}$ (nm)	a_1	τ_1 (ns)	a_2	τ_2 (ns)	τ_{av} (ns)
Tp-Acr	360	430	221	0.0212	6.02	5.26	0.16
DHTA-Acr	360	430	11.1	4.62			4.62
HTA-Acr	360	430	17.4	0.111	8.85	4.56	1.61

S5.7 EPR-results of Tp-Acr, DHTA-Acr and HTA-Acr COFs

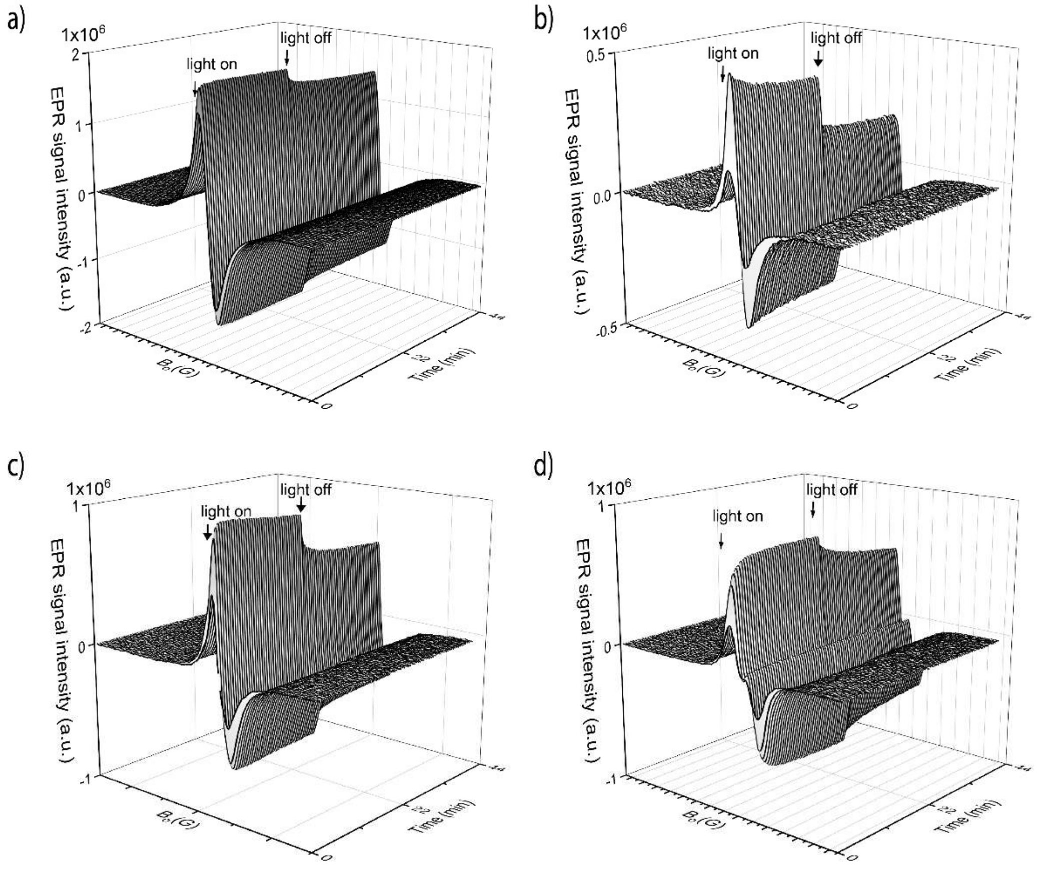

Figure S 18. Time resolved EPR conduction band e⁻ signals of (a) Tp-Acr, (b) DHTA-Acr, (c) HTA-Acr and (d) Tp-DAA under dark condition (0 min) and during visible light irradiation (0.5-22 min) and after the light was switched off (22.5-44 min).

S6. Synthesis and Characterization of Tp-DAA COF[6]

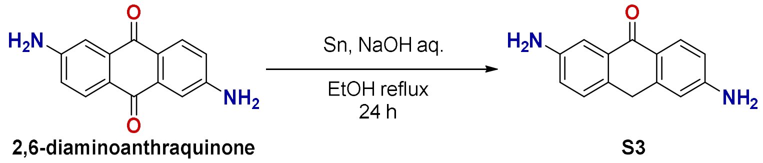

Scheme S 7. Synthesis of 2,6-diaminoanthrone (S3) from 2,6-diaminoanthraquinone.

2,6-diaminoanthrone (3): 2.50 g (10.5 mmol, 1.0 eq.) of 2,6-diaminoanthraquinone were combined with 7.47 g (63 mmol, 6.0 eq) of tin powder (> 150µm), 44 mL of 2.5 M aqueous NaOH and 50 mL of EtOH. The reaction was heated to reflux under argon atmosphere for 24 h, when the still hot reaction mixture was poured into 250 mL of H2O and stirred for 20 min. The resulting precipitate was filtered and dried under vacuo to give the product as a yellow-brown solid (1.80g, 77 %).

^{1}H NMR (200 MHz, DMSO-*d$_6$*) δ 7.87 (d, *J* = 8.6 Hz, 1H), 7.33 (d, *J* = 2.4 Hz, 1H), 7.15 (d, *J* = 8.1 Hz, 1H), 6.84 (dd, *J* = 8.1, 2.5 Hz, 1H), 6.62 (dd, *J* = 8.7, 2.0 Hz, 1H), 6.53 (d, *J* = 1.5 Hz, 1H), 6.04 (s, 2H), 5.19 (s, 2H), 4.04 (s, 2H). ^{13}C NMR (50 MHz, DMSO-*d$_6$*) δ 181.70, 152.91, 147.19, 143.63, 132.46, 128.90, 128.86, 127.72, 120.68, 119.06, 113.27, 110.56, 109.78, 30.97.

These data are in full agreement with those previously published in the literature.[7]

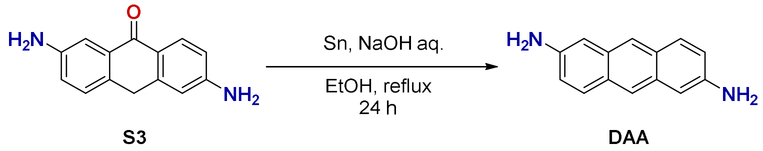

Scheme S 8. Synthesis of 2,6-diaminoanthracene from 2,6-diaminoanthrone (S3).

2,6-diaminoanthracene (DAA): 1.80 g (8 mmol, 1.0 eq.) of 2,6-diaminoantrhone (**S3**) were combined with 2.43 g (64 mmol, 8.0 eq.) of $NaBH_4$, 36 mL of 2.5 M aqueous NaOH and 40 mL of EtOH. The reaction mixture was refluxed for 6 h. The still hot reaction was poured into 280 mL of water and stirred for 15 min. The formed solid was filtered and washed with water (3x 20 mL) and EtOH (3x 20 mL). The product was dried under vacuo to give the product as a yellow-brown solid (810 mg, 48.6 %).

^{1}H NMR (700 MHz, DMSO-*d$_6$*) δ 7.82 (s, 2H), 7.63 (d, *J* = 8.9 Hz, 2H), 6.93 (dd, *J* = 8.9, 2.1 Hz, 2H), 6.79 (d, *J* = 1.7 Hz, 2H), 5.21 (s, 4H). ^{13}C NMR (176 MHz, DMSO-*d$_6$*) δ 144.05, 130.65, 127.96, 127.24, 121.35, 120.46, 103.76.

These data are in full agreement with those previously published in the literature.[8]

Tp-DAA COF: A Pyrex tube (o.d. × i.d. = 15 × 10 mm2 and length 15 cm) is charged with triformylphloroglucinol (**Tp**) (21 mg, 0.1 mmol), 2,6-diaminoanthracene (**DAA**) (31.2 mg, 0.15 mmol), 1.5 mL of mesitylene, 1.5 mL of dioxane and 0.5 mL of 6 M aqueous acetic acid. This mixture was sonicated for 15 minutes in order to get a homogenous dispersion. The tube was then flash frozen at 77 K (liquid N2 bath) and degassed by three freeze-pump-thaw cycles. The tube was sealed off and then heated at 120 °C for 3 days. A dark red colored precipitate was collected by filtration, washed with anhydrous acetone, methanol and cyclohexane to give a deep red colored powder (46 mg, 98 %).

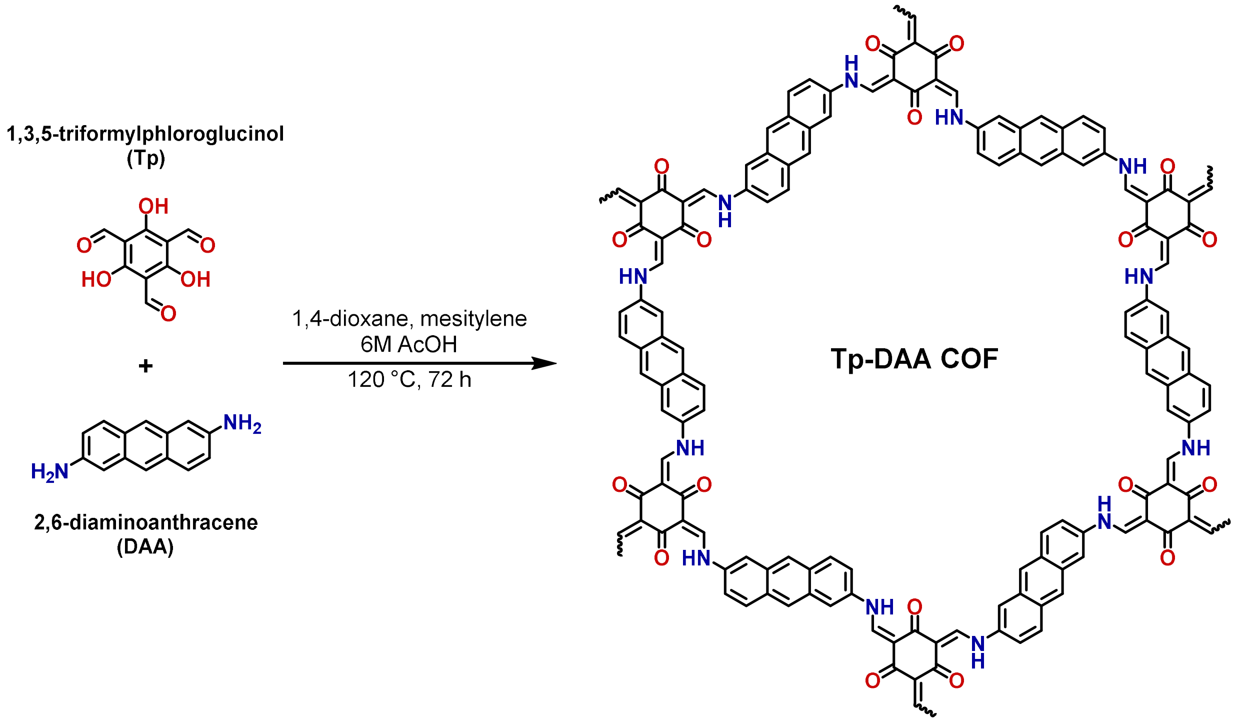

Scheme S 9. Synthesis of Tp-DAA COF from 1,3,5-triformylphloroglucinol (Tp) and 2,6-diaminoanthracene (DAA) using 1,4-dioxane: mesitylene (1:1) as solvent combination and acetic acid (6M) as catalyst.

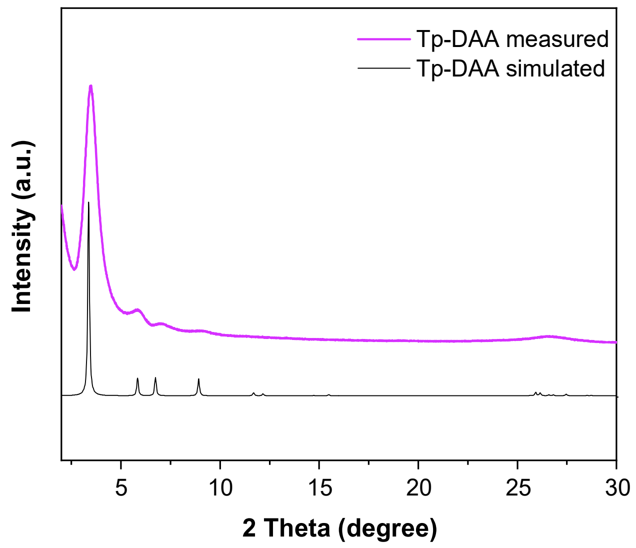

Figure S 19. Experimental and simulated powder X-ray diffraction pattern for Tp-DAA-COF (AA stacking) showing the matching of the pattern.

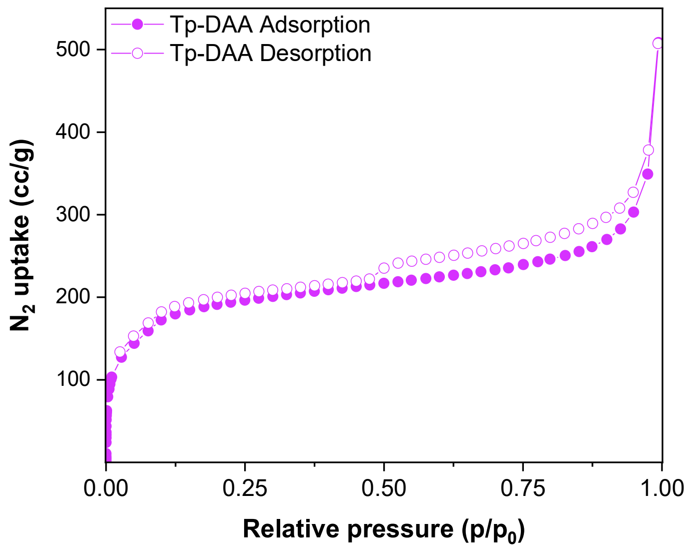

Figure S 20. N_2 sorption isotherms for Tp-DAA. Using Brunauer-Emmett-Teller (BET) analysis the surface area was calculated 716 m²/g for Tp-DAA.

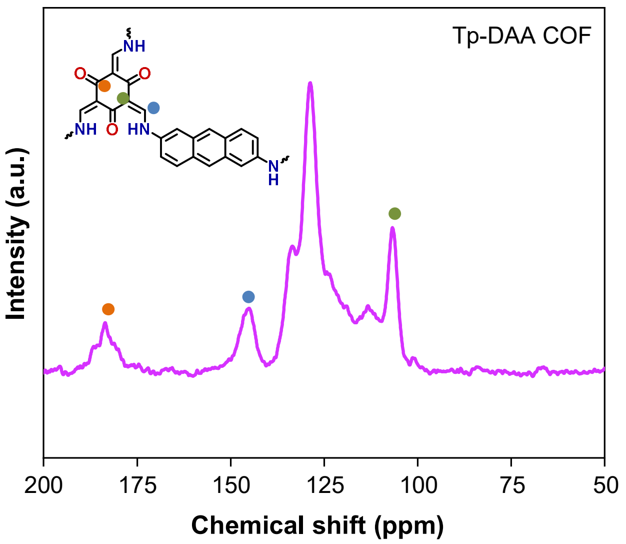

Figure S 21. ^{13}**C CP-MAS solid-state NMR spectra of Tp-DAA.**

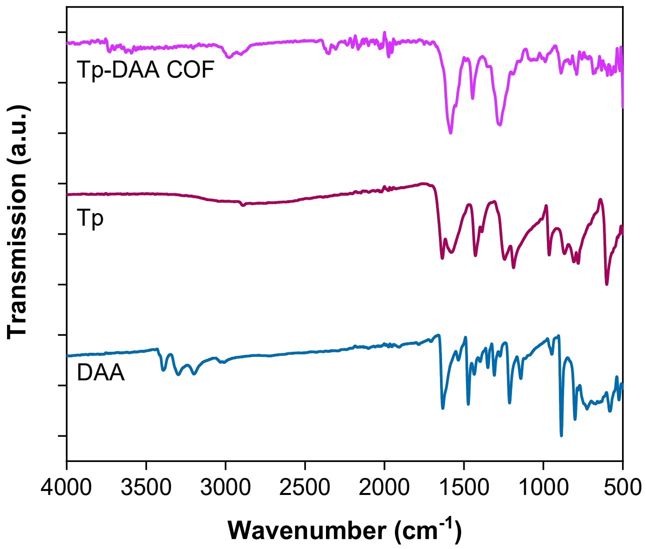

Figure S 22. FT-IR analyses of Tp-DAA COF in comparison with the corresponding aldehyde (Tp) and amine (DAA) showing the formation of the framework structure.

S7. Synthesis of model compound (SA-Acr)

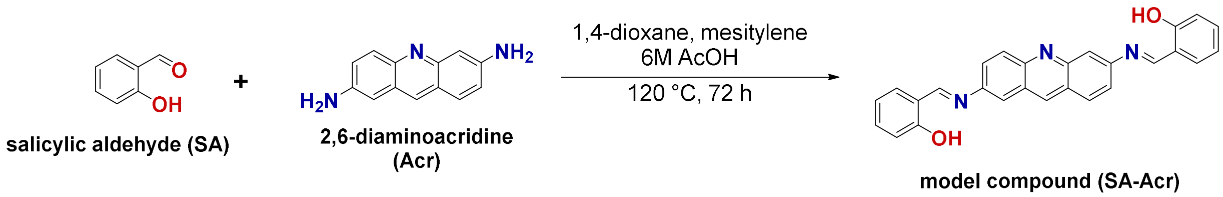

Scheme S 10. Synthesis of the model compound SA-Acr.

Model compound (SA-Acr): To a Schlenk flask were 63 mg (0.30 mmol, 1.0 eq.) of 2,6-diaminoacridine (Acr), 68 µl (0.66 mmol, 2.2 eq.) and 5 mL of 1,4-dioxane added. To the resulting suspension 1.0 mL of 6 M aqueous acetic acid were added. The flask was closed and heated to 100 °C for 24 h while stirring the reaction mixture. After cooling to room temperature, the resulting yellow-brown suspension was filtered and washed with 10 mL acetone to remove residual starting materials. The model compound was subsequently dried under vacuo to give the desired compound as a dark brown powder (71 mg, 57%).

ESI-MS [M+H$^+$] (m/z)): 418.1549 (th.: 418.1550).

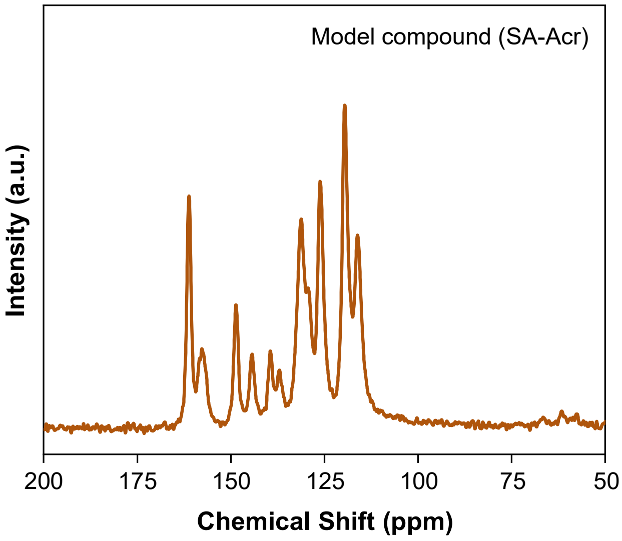

Figure S 23. ^{13}C CP-MAS solid-state NMR spectra of the model compound SA-Acr.

S8. Photocatalytic reaction optimization

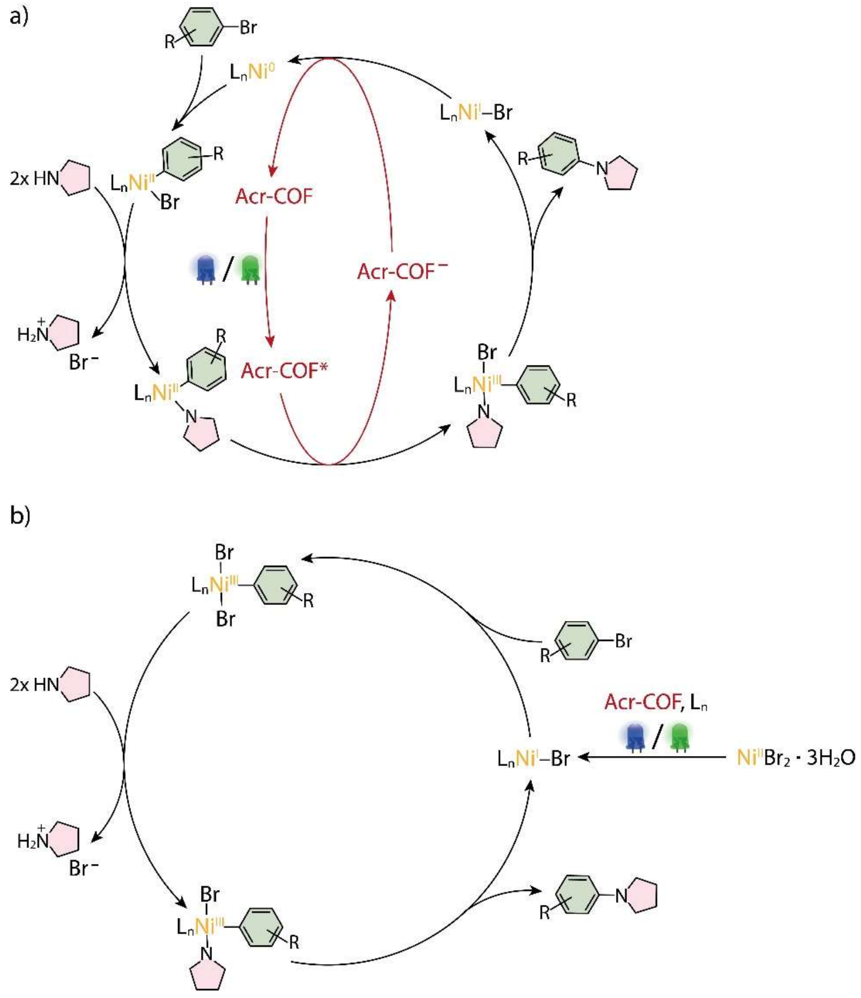

Figure S 24. Proposed mechanisms invoked in the nickel mediated metallaphotocatalytic C–N cross-coupling: a) oxidation-state modulation;[9] b) thermally sustained $Ni^{I/III}$ cycle.[10] L_n = pyrrolidine.

S8.1 General experimental procedure for screening experiments

An oven dried vial (16 x 100 mm) equipped with a stir bar was charged with the Ni^{II} catalyst (5-10 µmol, 5 mol%) and the Acridine-COF (2-4 mg). Subsequently, bromobenzotrifluoride (100-200 µmol) and pyrrolidine (300-450 µmol, 37.0 µL, 3.0 equiv.) and the solvent (anhydrous, 2 mL) were added and the vial was sealed with a septum and Parafilm. The reaction mixture was sonicated for 5-10 min followed by stirring for 5 min until a fine dispersion of the solids was achieved and the mixture was then degassed by bubbling N_2 for 15 min. The mixture was stirred at 800 rpm and irradiated with two LED lamps (440 nm) at full power. After the respective reaction time, one equivalent of 1,3,5-trimethoxybenzene (0.3 mmol, 50.5 mg) was added. An aliquot of the reaction mixture (~300 µL) was filtered, diluted with DMSO-d_6 and subjected to ^{1}H-NMR analysis.

S8.2 Screening of Acridine-COFs

Table S 5. Screening of acridine based COFs using two red LEDs.[a]

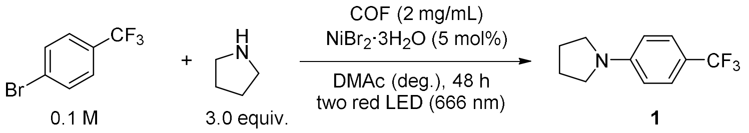

Entry	Acridine-COF	Conversion [%][b]	1 [%][c]
1	Tp-Acr	2	2
2	DHTA-Acr	13	n.d.[d]
3	HTA-Acr	11	1

[a]Reaction conditions: 4-bromobenzotrifluoride (0.2 mmol), pyrrolidine (0.6 mmol), $NiBr_2 \cdot 3H_2O$ (5 mol%), Acridine-COF (4 mg), DMAc (anhydrous, 2 mL), 2 red LED (100%) for 48h. [b]Conversion of 4-bromobenzotrifluoride determined by ^{1}H-NMR using 1,3,5-trimethoxybenzene as internal standard. [c]NMR yields determined by ^{1}H-NMR using 1,3,5-trimethoxybenzene as internal standard. [d]not detected.

S8.3 Control studies

Table S 6. Control studies using two green LEDs.[a]

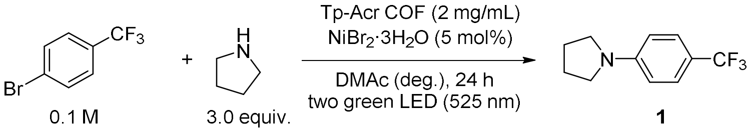

Entry	Deviation from standard conditions	Conversion [%][b]	1 [%][c]
1	None	54	52
2	Tp-DAA COF	28	27
3	2,6-diaminoacridine (5-mol%) instead of COF	6	n.d.[d]
4	Model compound (SA-Acr) instead of COF	9	n.d.
5	No Tp-Acr COF	7	3

[a]Reaction conditions: 4-bromobenzotrifluoride (0.2 mmol), pyrrolidine (0.6 mmol), $NiBr_2 \cdot 3H_2O$ (5 mol%), Acridine-COF (4 mg), DMAc (anhydrous, 2 mL), 2 green LED (100%) for 24h. [b]Conversion of 4-bromobenzotrifluoride determined by ^{1}H-NMR using 1,3,5-trimethoxybenzene as internal standard. [c]NMR yields determined by ^{1}H-NMR using 1,3,5-trimethoxybenzene as internal standard. [d]not detected.

S9. Photocatalysis - recycling studies

An oven dried vial (13 x 80 mm) equipped with a stir bar was charged with Acridine-COF (4 mg) and $NiBr_2 \cdot 3H_2O$ (4.1 mg, 15 μmol, 5 mol%). Subsequently, 4-bromobenzotrifluoride (67.5 mg, 42.0 μl, 0.3 mmol, 1.0 equiv.) and pyrrolidine (64.0 mg, 73.9 μl, 0.9 mmol, 3.0 equiv.) and DMAc (anhydrous, 3 mL) were added and the vial was sealed with a septum and Parafilm. The reaction mixture was sonicated for 5-10 min followed by stirring for 5 min until fine dispersion of the solids was achieved and the mixture was then degassed by bubbling N_2 for 10 min. The mixture was stirred at 800 rpm and irradiated with one or two LED lamps (440 nm) at full power. After the respective reaction time, one equivalent of 1,3,5-trimethoxybenzene (50.5 mg, 0.3 mmol) was added and the mixture was stirred for 5 min. The reaction mixture was centrifuged at 3500 rpm for 10 min and the liquid phase was carefully separated and analyzed by ^{1}H-NMR. The Acridine-COF was washed 2 times with DMAc (anhydrous, 3 mL, followed by centrifugation at 3500 rpm for 10 min and separation of the liquid phase), lyophilized (overnight) and reused in the next reaction. Alternatively, the Acridine-COF was washed first with methanol 3 mL(followed by centrifugation at 3000 rpm for 10 min and separation of the liquid phase) and then with hexane (followed by centrifugation at 3500 rpm for 10 min and separation of the liquid phase), dried under reduced pressure and reused in the next reaction.

Table S 7. Reusability of Tp-Acr-COF using 2 blue lamps.[a]

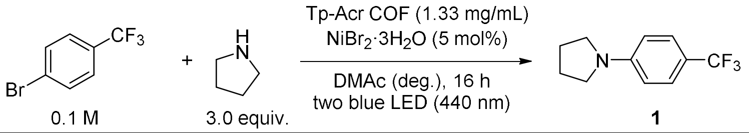

Cycle	1 [%][b]
1	98
2	82
3	73
4	41
5	65

[a]Reaction conditions: 4-bromobenzotrifluoride (0.3 mmol), pyrrolidine (0.9 mmol), $NiBr_2 \cdot 3H_2O$ (5 mol%), Acr-COF (4 mg - reused), DMAc (anhydrous, 3 mL), 2 blue LED (100%) for 16h. [b]NMR yields determined by ^{1}H-NMR using 1,3,5-trimethoxybenzene as internal standard.

Table S 8. Reusability of Tp-Acr COF using 1 blue lamp.[a]

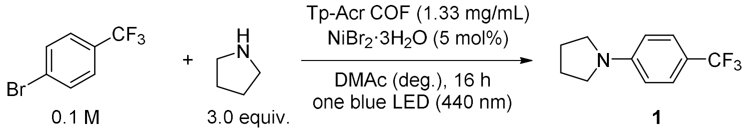

Cycle	1 [%][b]
1	53
2	52
3	49
4	27
5	23

[a]Reaction conditions: 4-bromobenzotrifluoride (0.3 mmol), pyrrolidine (0.9 mmol), $NiBr_2{\cdot}3H_2O$ (5 mol%), Acr-COF (4 mg - reused), DMAc (anhydrous, 3 mL), 1 blue LED (100%) for 16h. [b]NMR yields determined by ^{1}H-NMR using 1,3,5-trimethoxybenzene as internal standard.

Table S 9. Reusability of Tp-Acr COF using 2 blue lamp and adding only $NiBr_2{\cdot}3H_2O$ in the first cycle.[a]

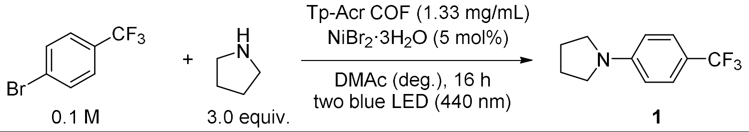

Cycle	1 [%][b]
1	95
2	6
3	6
4	10
5	12

[a]Reaction conditions: 4-bromobenzotrifluoride (0.3 mmol), pyrrolidine (0.9 mmol), $NiBr_2{\cdot}3H_2O$ (5 mol%), Acr-COF (4 mg - reused), DMAc (anhydrous, 3 mL), 2 blue LED (100%) for 16h. [b]NMR yields determined by ^{1}H-NMR using 1,3,5-trimethoxybenzene as internal standard.

Table S 10. Reusability of Tp-Acr COF using two blue lamp and washing with MeOH and hexane.[a]

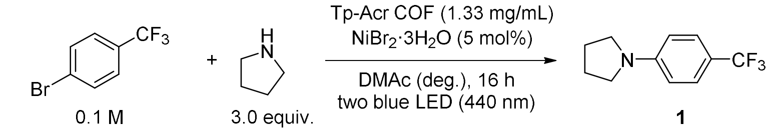

Cycle	1 [%][b]
1	94
2	95
3	82
4	65
5	63

[a]Reaction conditions: 4-bromobenzotrifluoride (0.3 mmol), pyrrolidine (0.9 mmol), $NiBr_2{\cdot}3H_2O$ (5 mol%), Acr-COF (4 mg - reused), DMAc (anhydrous, 3 mL), 2 blue LED (100%) for 16h. [b]NMR yields determined by ^{1}H-NMR using 1,3,5-trimethoxybenzene as internal standard.

S9.1 Analysis of recycled Tp-Acr COF

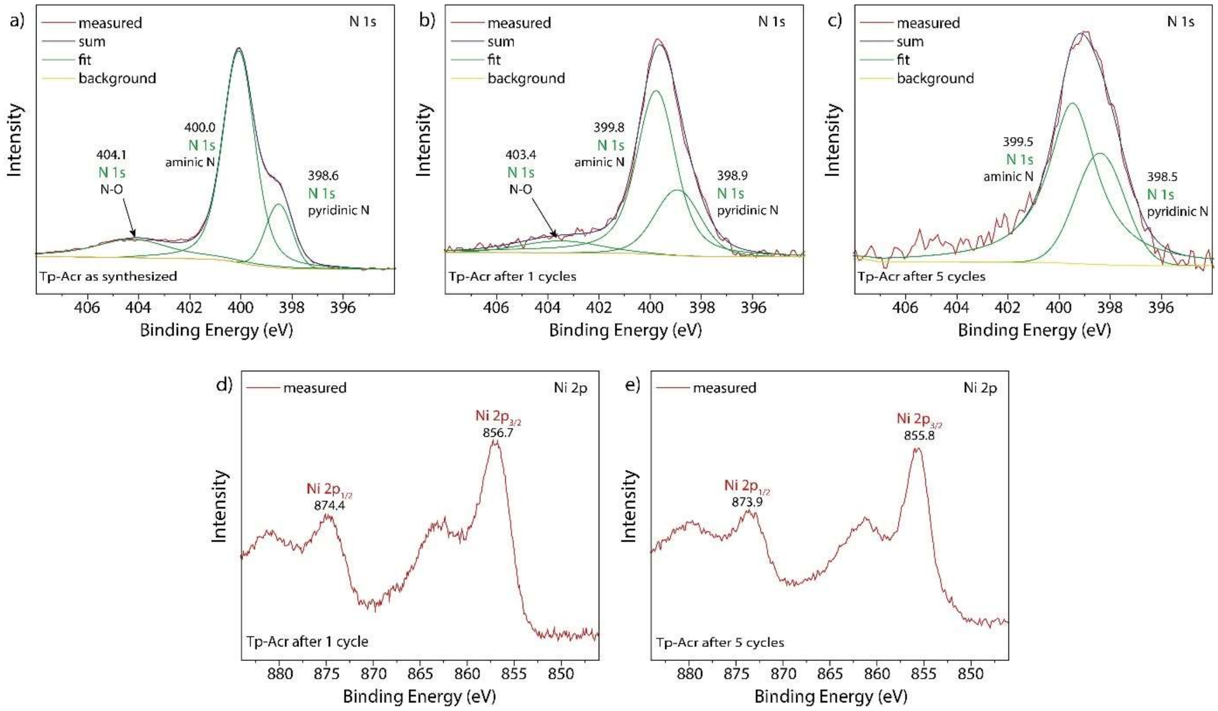

Figure S 25. (a, b, c) N 1s XPS core level spectra of the TP-Acr COF as synthesized (a), after the first reaction cycle (b) and after 5 recycling cycles (c) of photocatalytic dual nickel C-N cross coupling. (d, e) Ni 2p XPS core level spectra of the TP-Acr COF after the first reaction cycle (d) and after 5 recycling cycles (e) of photocatalytic dual nickel C-N cross coupling.

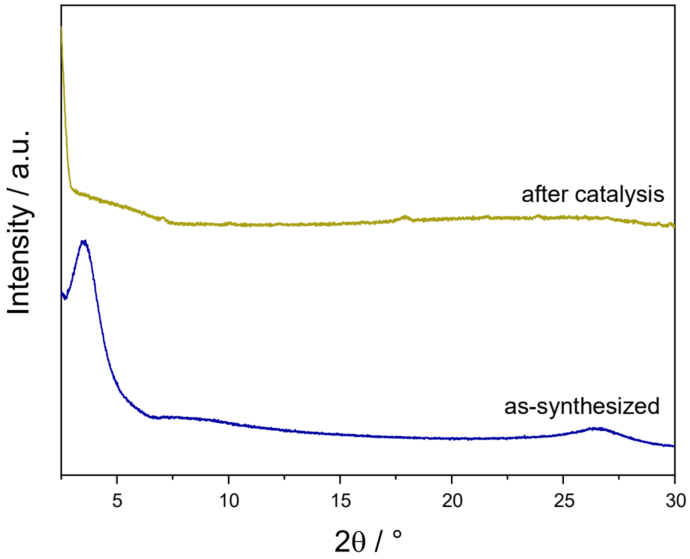

Figure S 26. FT-IR analyses of Tp-Acr COF before and after photocatalysis, showing that the framework structure loses crystallinity under photocatalysis conditions

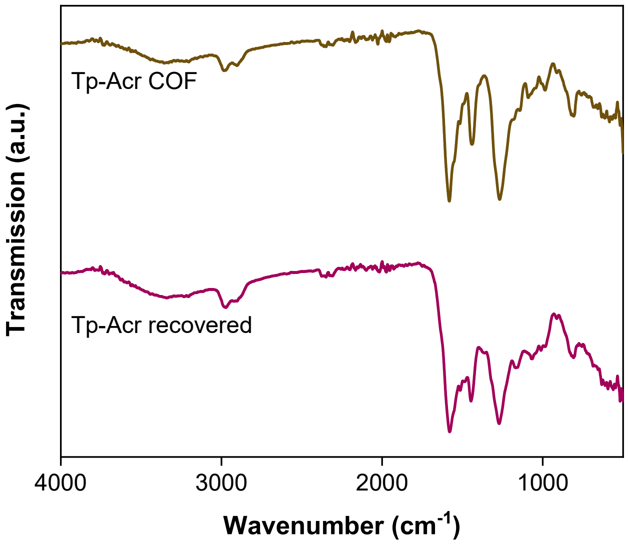

Figure S 27. FT-IR analyses of Tp-Acr COF before and after photocatalysis, showing that the framework structure remains stable under photocatalysis conditions.

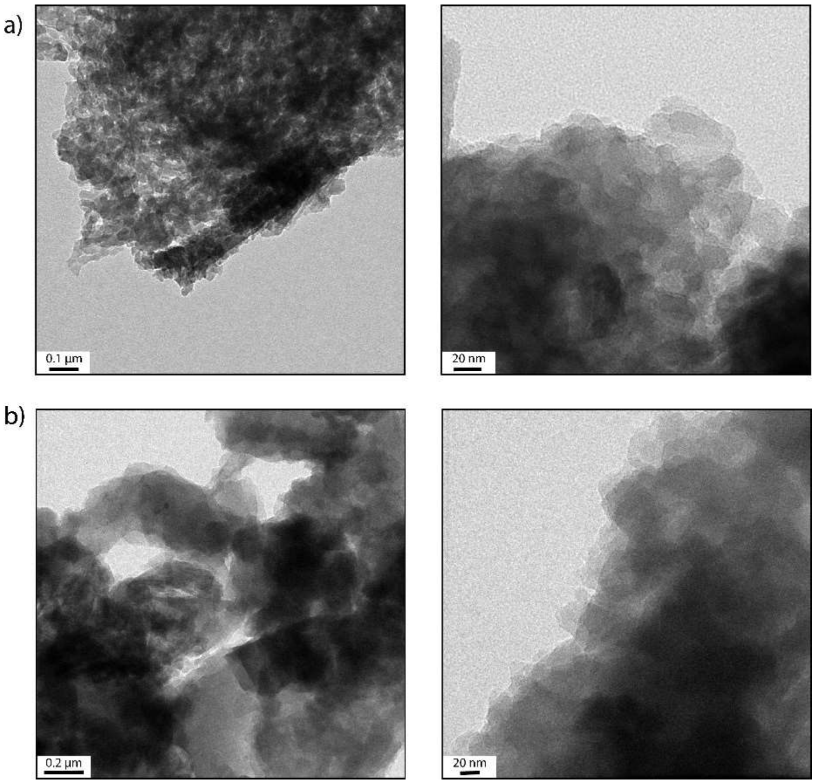

Figure S 28. TEM analyses of Tp-Acr after 1 (a) and 5 (b) cycles of photocatalysis, respectively, showing the morphology and the layered structure of the COF matrix

S10. Scope and limitations

General experimental procedure. An oven dried vial (13 x 95 mm) equipped with a stir bar was charged with $NiBr_2 \cdot 3H_2O$ (4.1 mg, 15 µmol, 5 mol%), aryl bromide (0.3 mmol, 1 equiv.) and Tp-2,6-Acr-COF (4.0 mg). Subsequently, the amine (0.9 mmol, 3 equiv.) and DMAc (anhydrous, 3 mL) were added and the vial was sealed with a septum and parafilm. The reaction mixture was sonicated for 5-10 min and the mixture was then degassed by bubbling N_2 for 10 min. The mixture was stirred at 800 rpm and irradiated with two LED lamps (440 nm) at full power. After the respective reaction time, one equivalent of 1,3,5-trimethoxybenzene (50.5 mg, 0.3 mmol, internal standard) was added. An aliquot (~300 µL) of the reaction mixture was diluted with DMSO-d_6 and subjected to ^{1}H-NMR analysis. After full consumption of the arene starting material, the liquid phase was diluted with H_2O (40 mL) and extracted with ethyl acetate (3 x 30 mL). The combined organic phases were washed with H_2O (40 mL), $NaHCO_3$ solution (40 mL) and brine (40 mL), dried over Na_2SO_4 and concentrated. The crude product was purified by flash column chromatography (SiO_2, Hexane/EtOAc). The final product was characterized by ^{1}H-NMR, ^{13}C-NMR, ^{19}F-NMR.

Table S 11. Scope of the C–N coupling reaction.[a]

Product	Reaction time	NMR yield[b]	Isolated yield
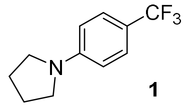 **1**	16 hours	95%	93%
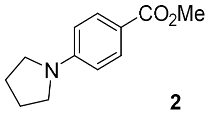 **2**	16 hours	90%	88%
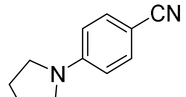 **3**	24 hours	97%	94%
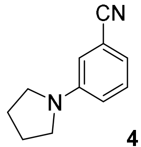 **4**	48 hours	94%	91%
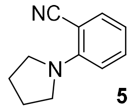 **5**	72 hours	92%	87%
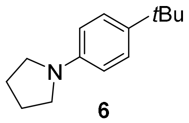 **6**	48 hours	68%	66%

[a]Reaction conditions according to general procedure. [b]NMR yields determined by ^{1}H-NMR using maleic acid as internal standard

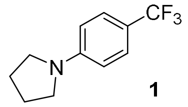

1

1-(4-(trifluoromethyl)phenyl)pyrrolidine: From pyrrolidine (64.0 mg, 73.9 µl, 0.9 mmol, 3.0 equiv.) and 4-bromobenzotrifluoride (67.5 mg, 42.0 µl, 0.3 mmol, 1.0 equiv.). Reaction time: 16 h. Purification with flash chromatography (3% ethyl acetate in hexane) afforded the title compound (59.8 mg, 0.28 mmol, 93%) as a white solid.

^{1}H NMR (400 MHz, $CDCl_3$) δ 7.45 (d, J = 8.6 Hz, 2H), 6.56 (d, J = 8.6 Hz, 2H), 3.45 – 3.20 (m, 4H), 2.16 – 1.94 (m, 4H). ^{13}C NMR (101 MHz, $CDCl_3$) δ 149.9, 126.5 (q, J = 3.7 Hz), 125.5 (q, J = 270.0 Hz), 116.7 (q, J = 32.4 Hz), 110.9, 47.6, 25.6. ^{19}F NMR (376 MHz, $CDCl_3$) δ -60.5 (s, 3F).

These data are in full agreement with those previously published in the literature.[11]

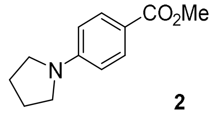

2

1-(4-methylbenzoate)pyrrolidine: From pyrrolidine (64.0 mg, 73.9 µl, 0.9 mmol, 3.0 equiv.) and 4-bromomethylbenzoate (64.5 mg, 0.3 mmol, 1.0 equiv.). Reaction time: 16 h. Purification with flash chromatography (5% ethyl acetate in hexane) afforded the title compound (54.3 mg, 0.26 mmol, 88%).

^{1}H NMR (400 MHz, $CDCl_3$) δ 7.89 (d, J = 8.9 Hz, 2H), 6.50 (d, J = 8.9 Hz, 2H), 3.85 (s, 3H), 3.50 – 3.19 (m, 4H), 2.13 – 1.90 (m, 4H).^{13}C NMR (101 MHz, $CDCl_3$) δ 167.8, 150.9, 131.5, 116.3, 110.7, 51.5, 47.6, 25.6.

These data are in full agreement with those previously published in the literature.[12]

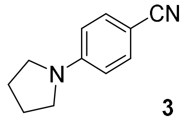

3

1-(4-benzonitrile)pyrrolidine: From pyrrolidine (64.0 mg, 73.9 µl, 0.9 mmol, 3.0 equiv.) and 4-bromobenzonitrile (54.6 mg, 0.3 mmol, 1.0 equiv.). Reaction time: 24 h. Purification with flash chromatography (5% ethyl acetate in hexane) afforded the title compound (48.5 mg, 0.28 mmol, 94%) as a white solid.

^{1}H NMR (400 MHz, $CDCl_3$) δ 7.43 (d, J = 8.8 Hz, 2H), 6.49 (d, J = 8.9 Hz, 2H), 3.44 – 3.19 (m, 4H), 2.16 – 1.94 (m, 4H). ^{13}C NMR (101 MHz, $CDCl_3$) δ= 150.1, 133.5, 121.16, 111.5, 96.6, 47.6, 25.5.

These data are in full agreement with those previously published in the literature.[13]

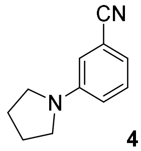

4

1-(3-benzonitrile)pyrrolidine: From pyrrolidine (64.0 mg, 73.9 µl, 0.9 mmol, 3.0 equiv.) and 3-bromobenzonitrile 4-bromobenzonitrile (54.6 mg, 0.3 mmol, 1.0 equiv.). Reaction time: 48 h. No internal standard (1,3,5-trimethoxybenzene was used) due to poor separation from the product during flash chromatography. Purification with flash chromatography (1. gradient 3-5% ethyl acetate in hexane; 2. Isocratic 5% ethyl acetate in hexane) afforded the title compound (47.1 mg, 0.27 mmol, 91%) as a white solid.

^{1}H NMR (400 MHz, $CDCl_3$) δ 7.33 – 7.21 (m, 1H), 6.91 (d, J = 7.5 Hz, 1H), 6.81 – 6.71 (m, 2H), 3.36 – 3.23 (m, 4H), 2.13 – 1.98 (m, 4H).). ^{13}C NMR (101 MHz, $CDCl_3$) δ 147.7, 129.8, 120.0, 118.6, 115.9, 114.4, 112.8, 47.7, 25.5.

These data are in full agreement with those previously published in the literature.[14]

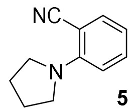

5

1-(2-benzonitrile)pyrrolidine: From pyrrolidine (64.0 mg, 73.9 µl, 0.9 mmol, 3.0 equiv.) and 2-bromobenzonitrile 4-bromobenzonitrile (54.6 mg, 0.3 mmol, 1.0 equiv.). Reaction time: 72 h. No internal standard (1,3,5-trimethoxybenzene was used) due to poor separation from the product during flash chromatography. Purification with flash chromatography (eluents: 1. gradient 3-5% ethyl acetate in hexane; 2. Isocratic 5% ethyl acetate in hexane) afforded the title compound (45.0 mg, 0.26 mmol, 87%) as a colorless oil.

^{1}H NMR (400 MHz, $CDCl_3$) δ 7.43 (m, 1H), 7.32 (m, 1H), 6.67 – 6.62 (m, 2H), 3.65 – 3.53 (m, 4H), 2.06 – 1.94 (m, 4H). ^{13}C NMR (101 MHz, $CDCl_3$) δ 150.1, 135.8, 133.5, 121.6, 116.0, 114.3, 94.4, 49.9, 25.8.

These data are in full agreement with those previously published in the literature.[15]

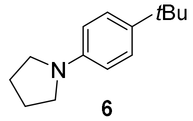

6

1-(4-(*tert*-butyl)phenyl)pyrrolidine: From pyrrolidine (64.0 mg, 73.9 µl, 0.9 mmol, 3.0 equiv.) and 4-bromo-*tert*-butylbenzene (63.9 mg, 52.2 µl, 0.3 mmol, 1.0 equiv.). Reaction time: 48 h. Purification with flash chromatography (3% ethyl acetate in hexane) afforded the title compound (40.3 mg, 0.20 mmol, 66%) as a colorless oil.

^{1}H NMR (400 MHz, $CDCl_3$) δ 7.34 (d, J = 8.7 Hz, 2H), 6.61 (d, J = 8.7 Hz, 2H), 3.42 – 3.30 (m, 4H), 2.11 – 2.02 (m, 4H), 1.40 (s, 9H). ^{13}C NMR (101 MHz, $CDCl_3$) δ 145.9, 138.0, 126.0, 111.4, 47.8, 33.8, 31.7, 25.6.

These data are in full agreement with those previously published in the literature.[12]

S11. References

[1] "https://www.kessil.com/photoreaction/PR160L.php," **n.d.**

[2] D. H. O'Donovan, B. Kelly, E. Diez-Cecilia, M. Kitson, I. Rozas, *New J. Chem.* **2013**, *37*, 2408–2418.

[3] J. H. Chong, M. Sauer, B. O. Patrick, M. J. MacLachlan, *Org. Lett.* **2003**, *5*, 3823–3826.

[4] S. Haldar, D. Chakraborty, B. Roy, G. Banappanavar, K. Rinku, D. Mullangi, P. Hazra, D. Kabra, R. Vaidhyanathan, *J. Am. Chem. Soc.* **2018**, *140*, 13367–13374.

[5] A. A. Anderson, T. Goetzen, S. A. Shackelford, S. Tsank, *Synth. Commun.* **2000**, *30*, 3227–3232.

[6] M. A. Khayum, S. Kandambeth, S. Mitra, S. B. Nair, A. Das, S. S. Nagane, R. Mukherjee, R. Banerjee, *Angew. Chem. Int. Ed.* **2016**, *55*, 15604–15608.

[7] R. Kantam, R. Holland, B. P. Khanna, K. D. Revell, *Tetrahedron Lett.* **2011**, *52*, 5083–5085.

[8] I. Rose, M. Carta, R. Malpass-Evans, M. C. Ferrari, P. Bernardo, G. Clarizia, J. C. Jansen, N. B. McKeown, *ACS Macro Lett.* **2015**, *4*, 912–915.

[9] E. B. Corcoran, M. T. Pirnot, S. Lin, S. D. Dreher, D. A. Dirocco, I. W. Davies, S. L. Buchwald, D. W. C. Macmillan, *Science* **2016**, *353*, 279–283.

[10] R. Sun, Y. Qin, D. G. Nocera, *Angew. Chem. Int. Ed.* **2020**, *59*, 9527–9533.

[11] E. Brenner, R. Schneider, Y. Fort, *Tetrahedron* **1999**, *55*, 12829–12842.

[12] M. H. S. A. Hamid, C. L. Allen, G. W. Lamb, A. C. Maxwell, H. C. Maytum, A. J. A. Watson, J. M. J. Williams, *J. Am. Chem. Soc.* **2009**, *131*, 1766–1774.

[13] C. Desmarets, R. Schneider, Y. Fort, *J. Org. Chem.* **2002**, *67*, 3029–3036.

[14] J. P. Wolfe, H. Tomori, J. P. Sadighi, J. Yin, S. L. Buchwald, *J. Org. Chem.* **2000**, *65*, 1158–1174.

[15] P. Yu, B. Morandi, *Angew. Chem.* **2017**, *129*, 15899–15903.

S12. Author Contributions

All authors contributed extensively to the work presented in this paper. M.T., P.P. and A.T. conceived the research project. M.T. conducted the synthesis of linkers as well as COFs and performed the characterizations. S.G. performed and evaluated the photocatalytic experiments. J.S. performed and evaluated the XPS analyses. J. Roeser. performed the simulations of the COFs. S.R. performed the photoluminescence measurements and photocatalytic experiments. J. Rabeah performed the EPR analyses. The whole project was directed by A.T and B.P. with the help from M.T. and P.P., M.T. wrote the manuscript with the input from the other authors.

S13. Copies of NMR spectra

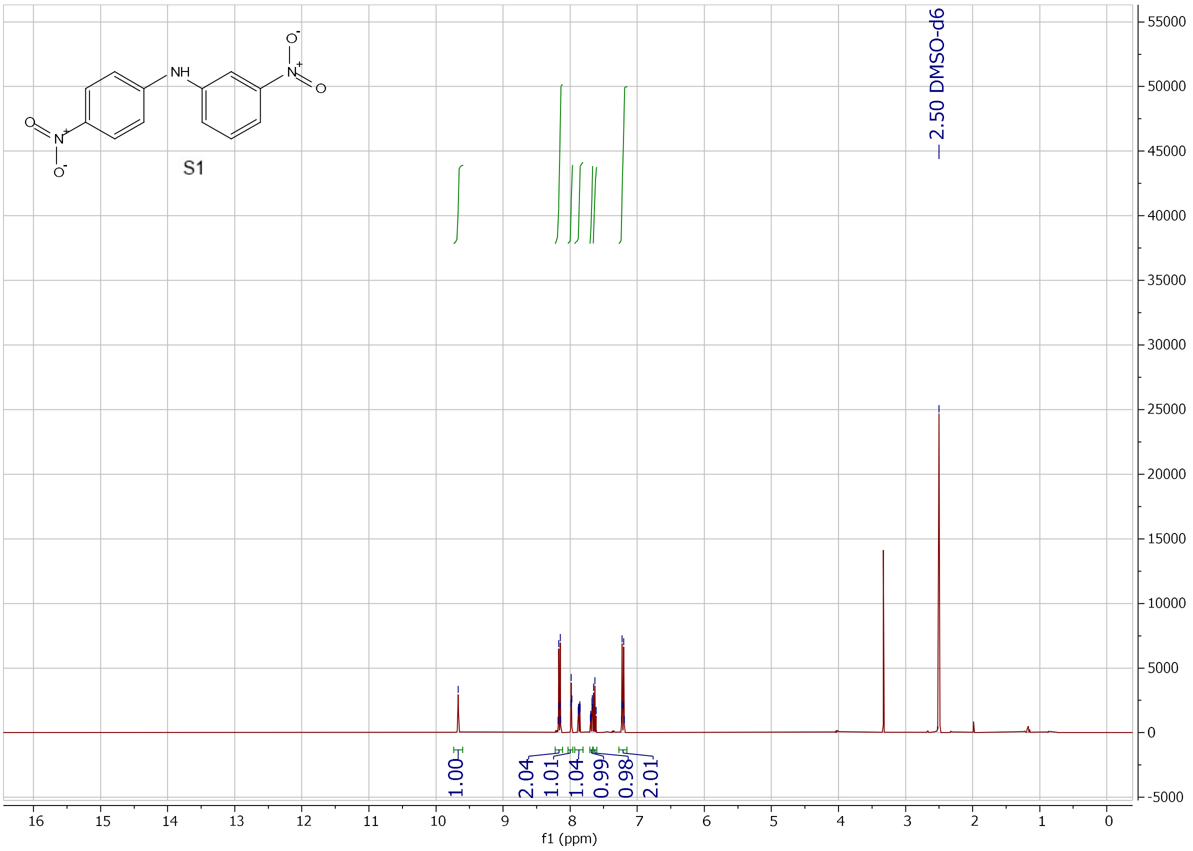

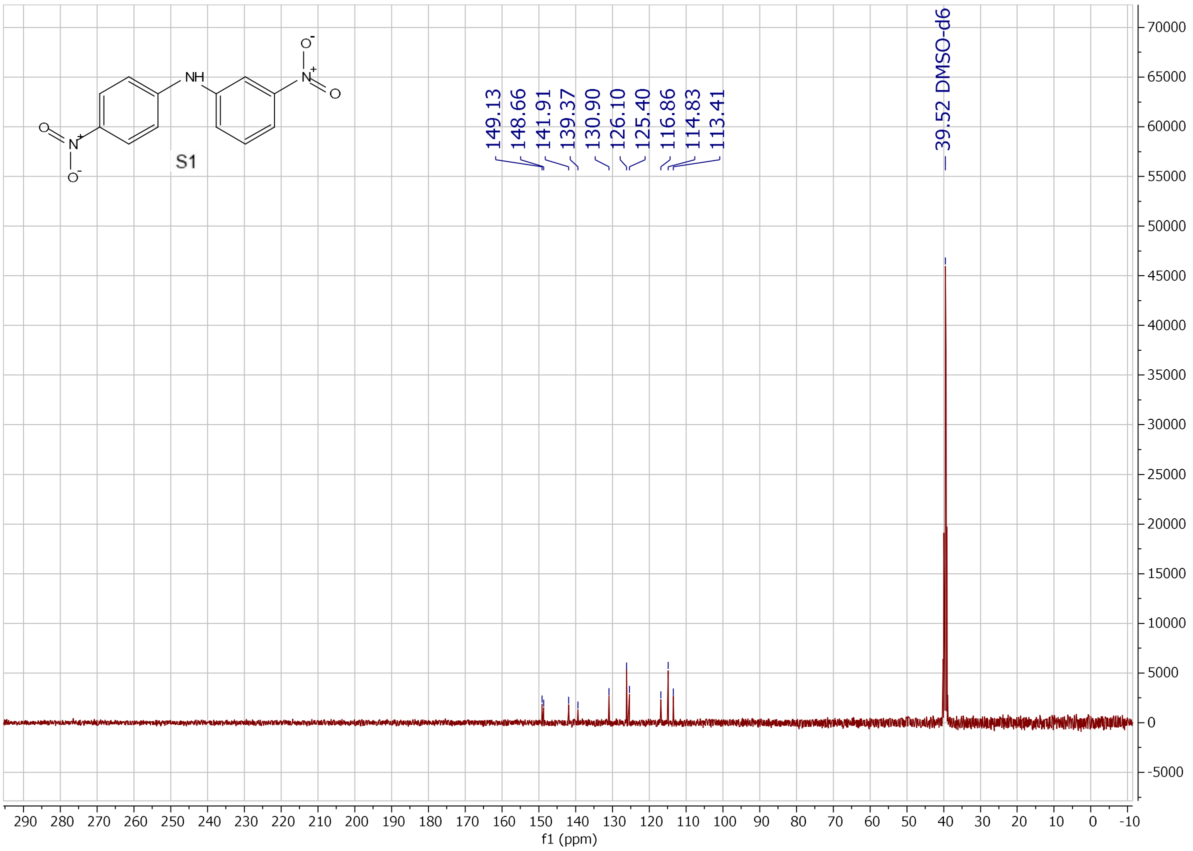

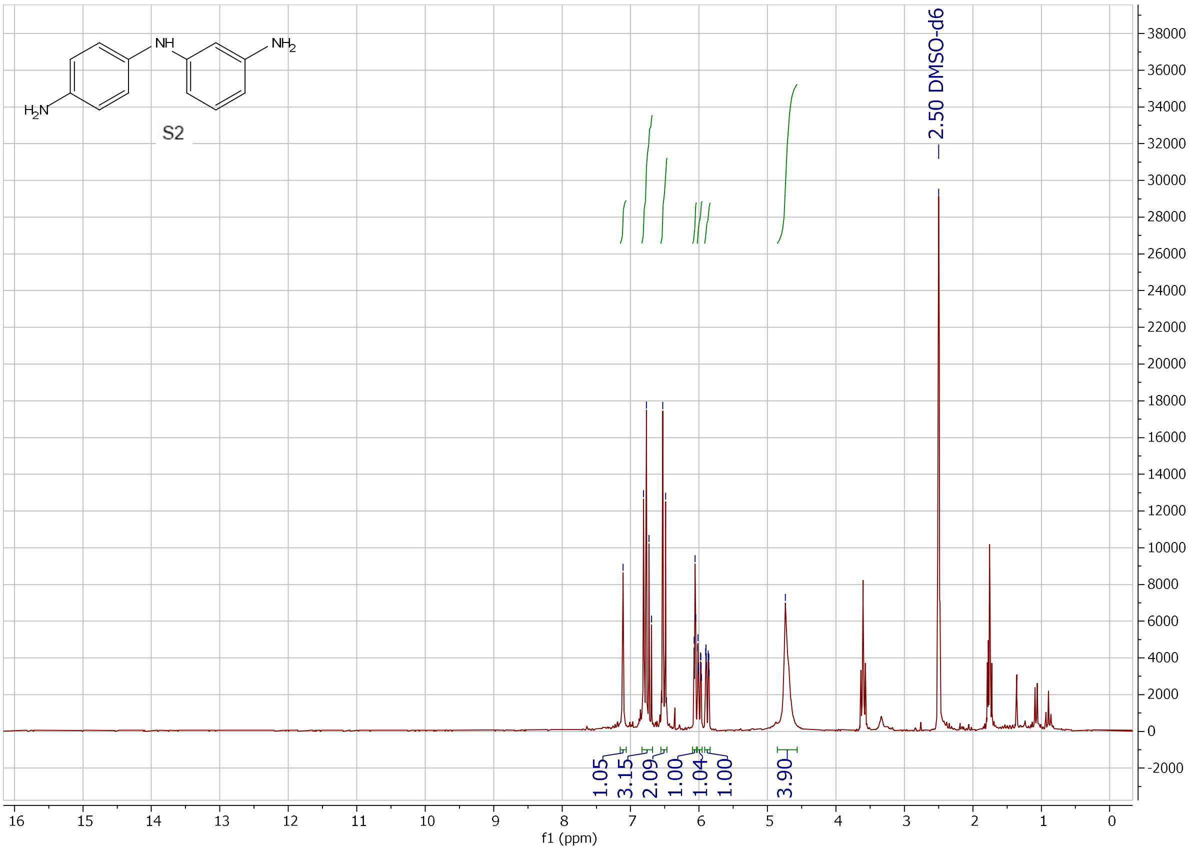
NH
NH2
H2N
S2
2.50 DMSO-d6
1.05
3.15
2.09
1.00
1.04
1.00
3.90
f1 (ppm)

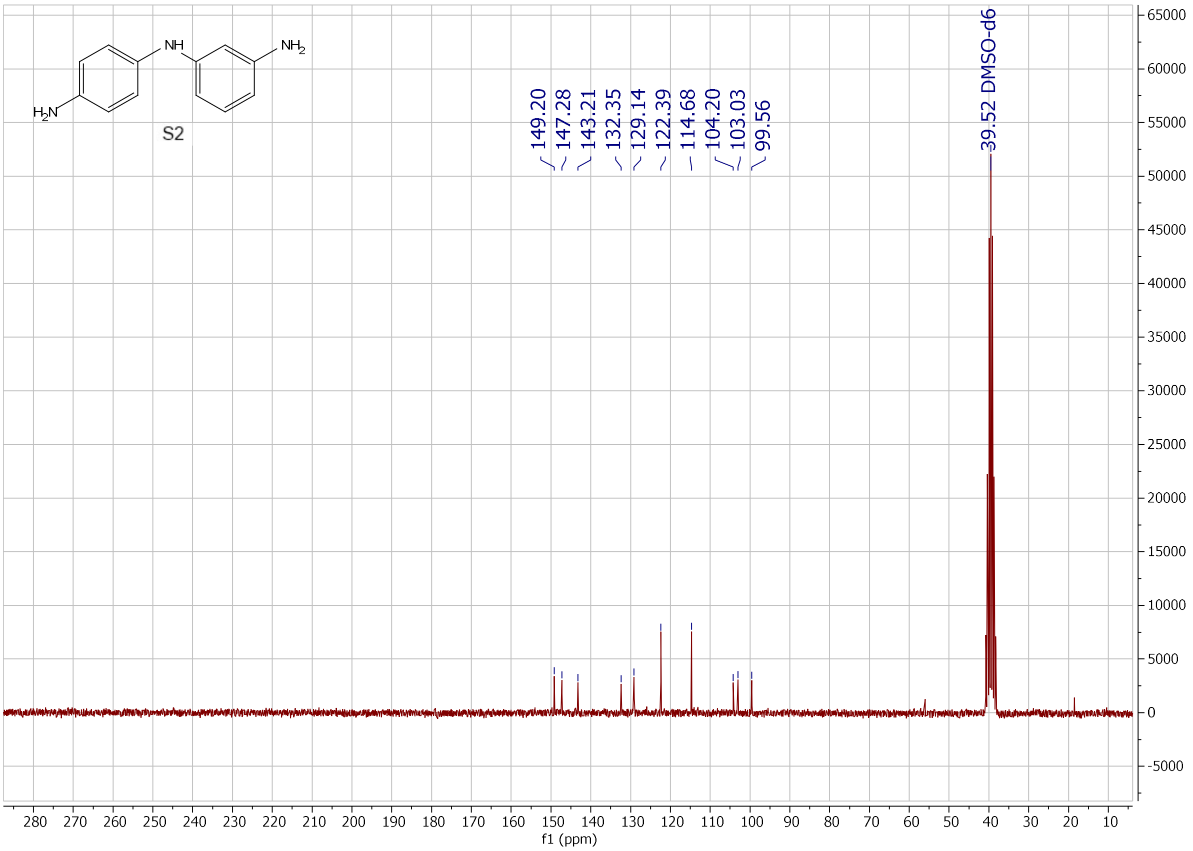
NH
NH2
H2N
S2
149.20
147.28
143.21
132.35
129.14
122.39
114.68
104.20
103.03
99.56
39.52 DMSO-d6
f1 (ppm)

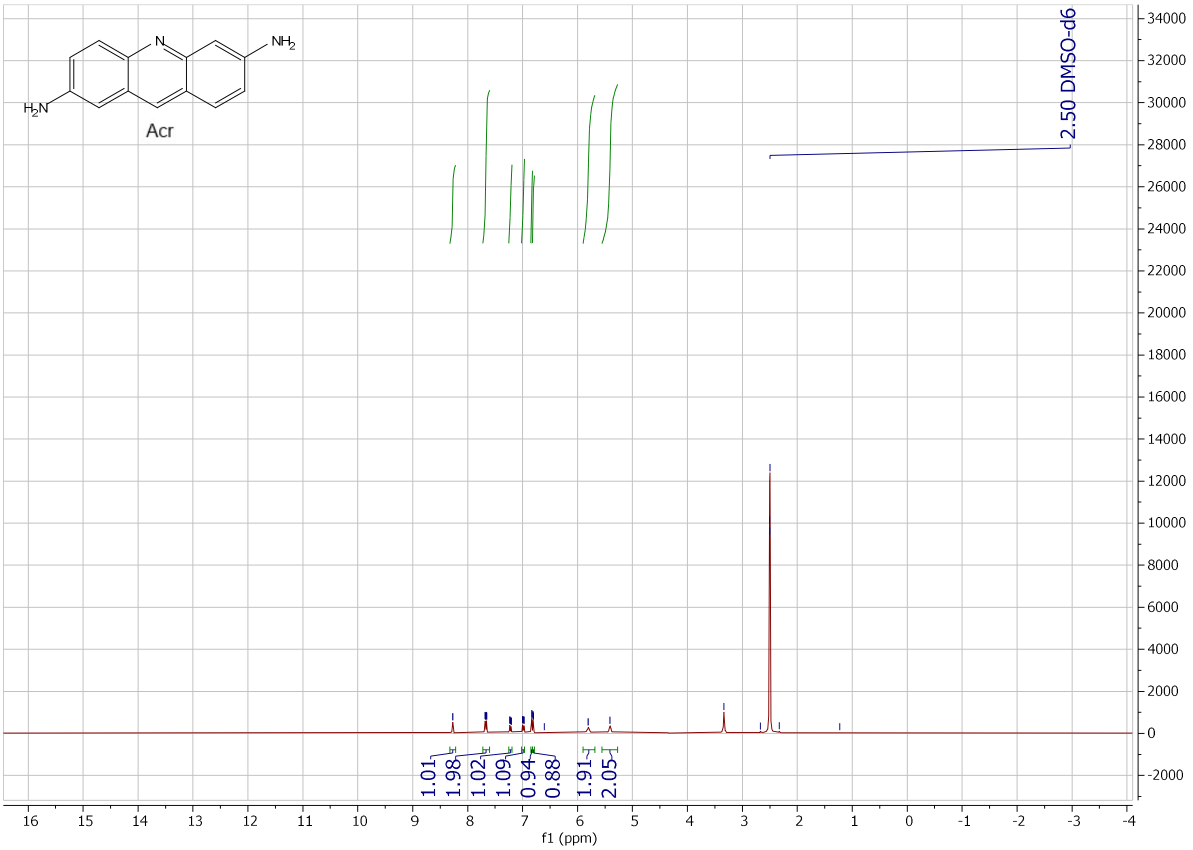

NH2
H2N
N
Acr
2.50 DMSO-d6
1.01
1.98
1.02
1.09
0.94
0.88
1.91
2.05
f1 (ppm)

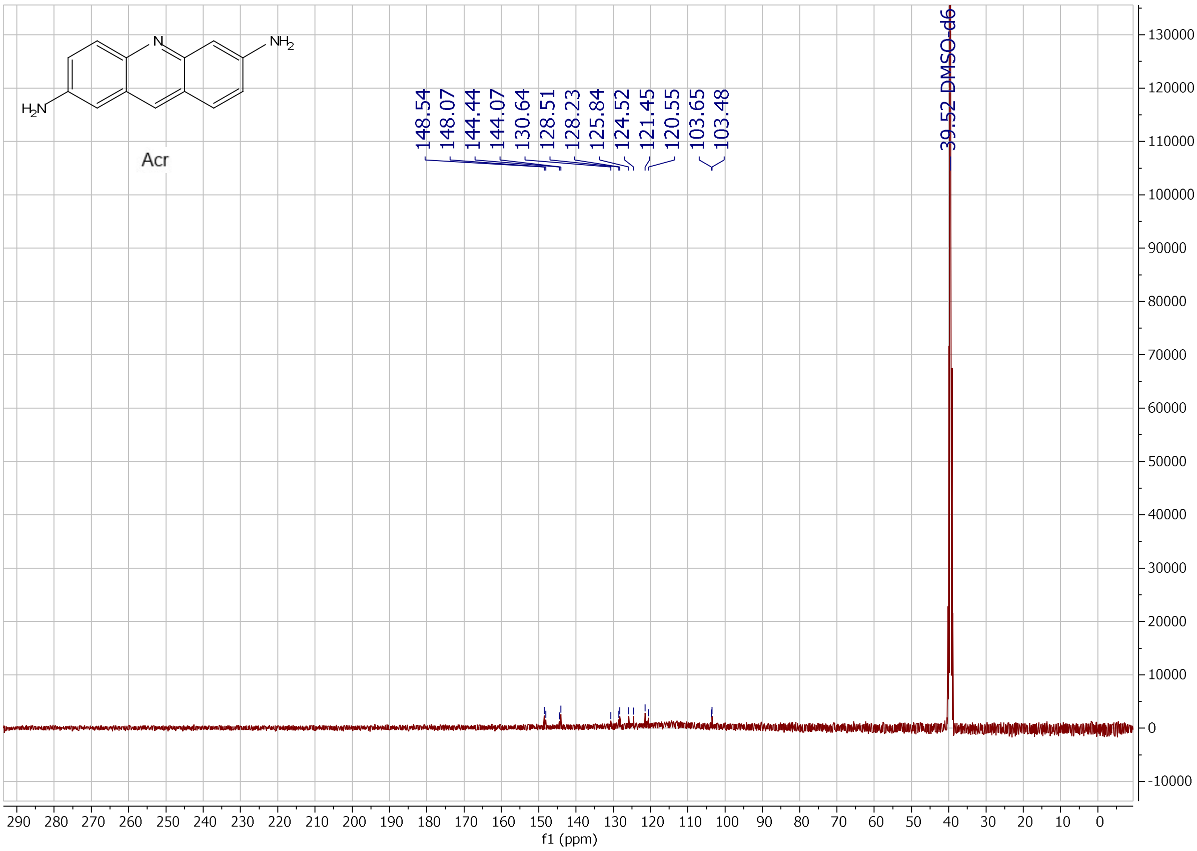

NH2
H2N
N
Acr
148.54
148.07
144.44
144.07
130.64
128.51
128.23
125.84
124.52
121.45
120.55
103.65
103.48
39.52 DMSO-d6
f1 (ppm)

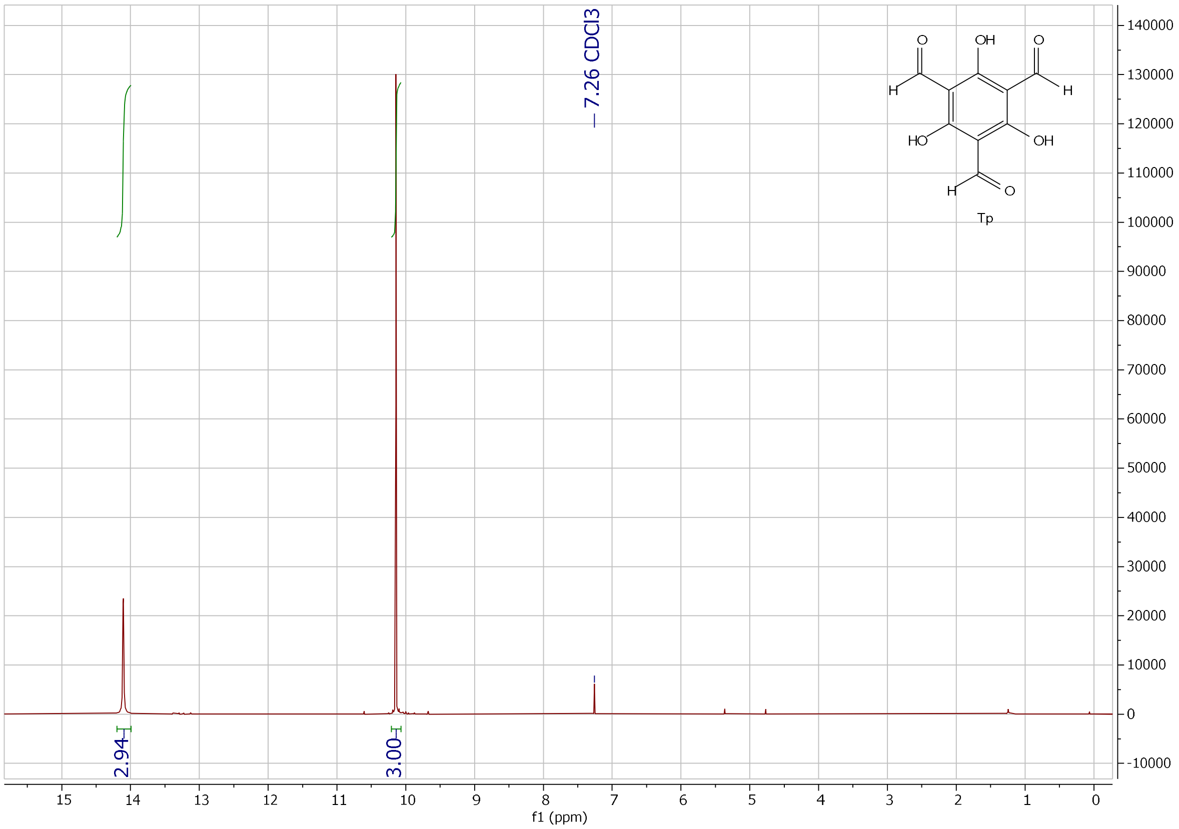
7.26 CDCl3
OH
O
O
H
H
HO
OH
H
O
Tp
2.94
3.00
f1 (ppm)

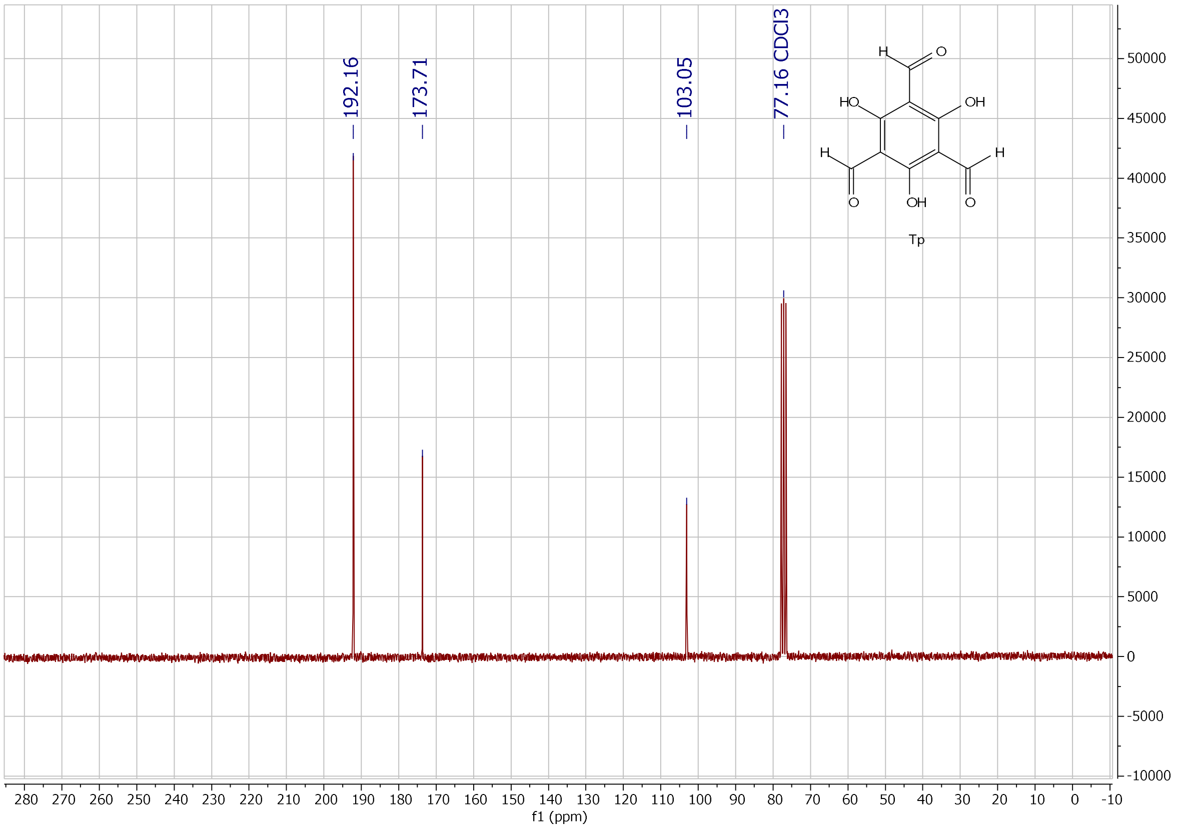
192.16
173.71
103.05
77.16 CDCl3
H
O
HO
OH
H
H
O
OH
O
Tp
f1 (ppm)

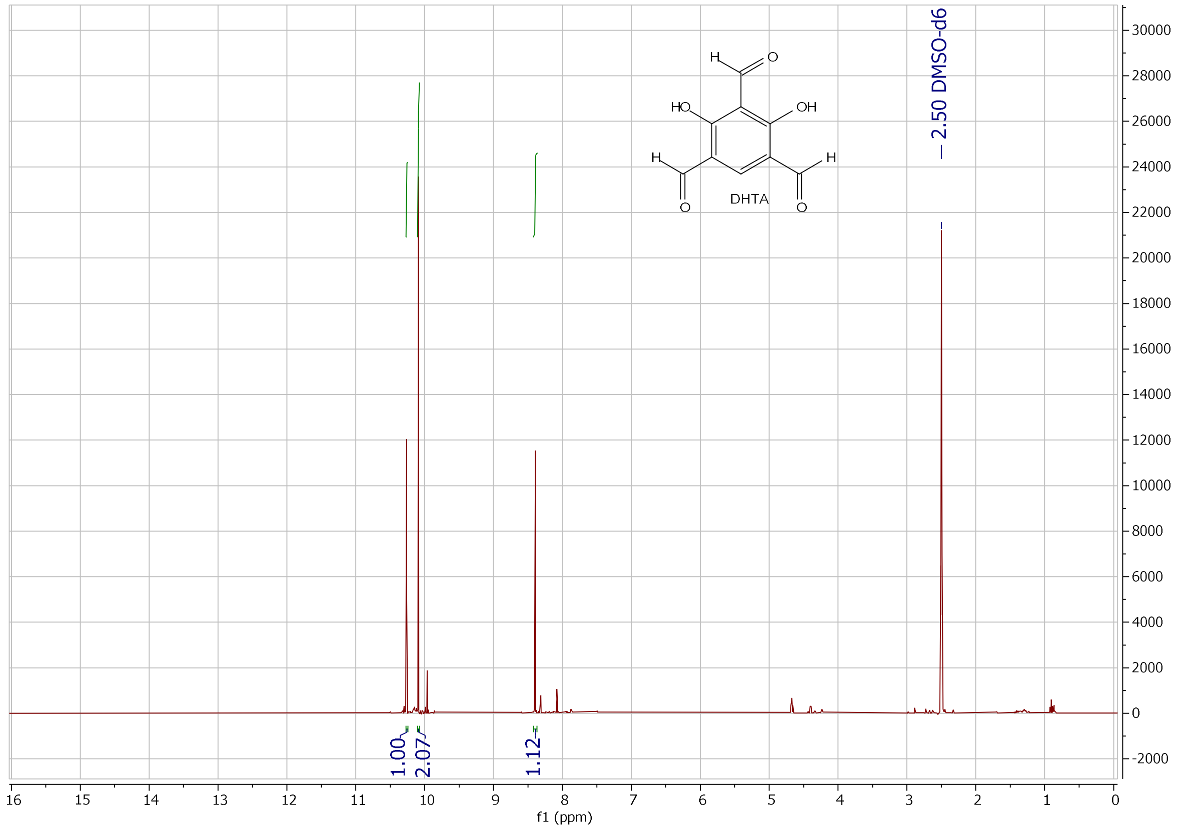

DHTA
2.50 DMSO-d6
1.00
2.07
1.12
f1 (ppm)

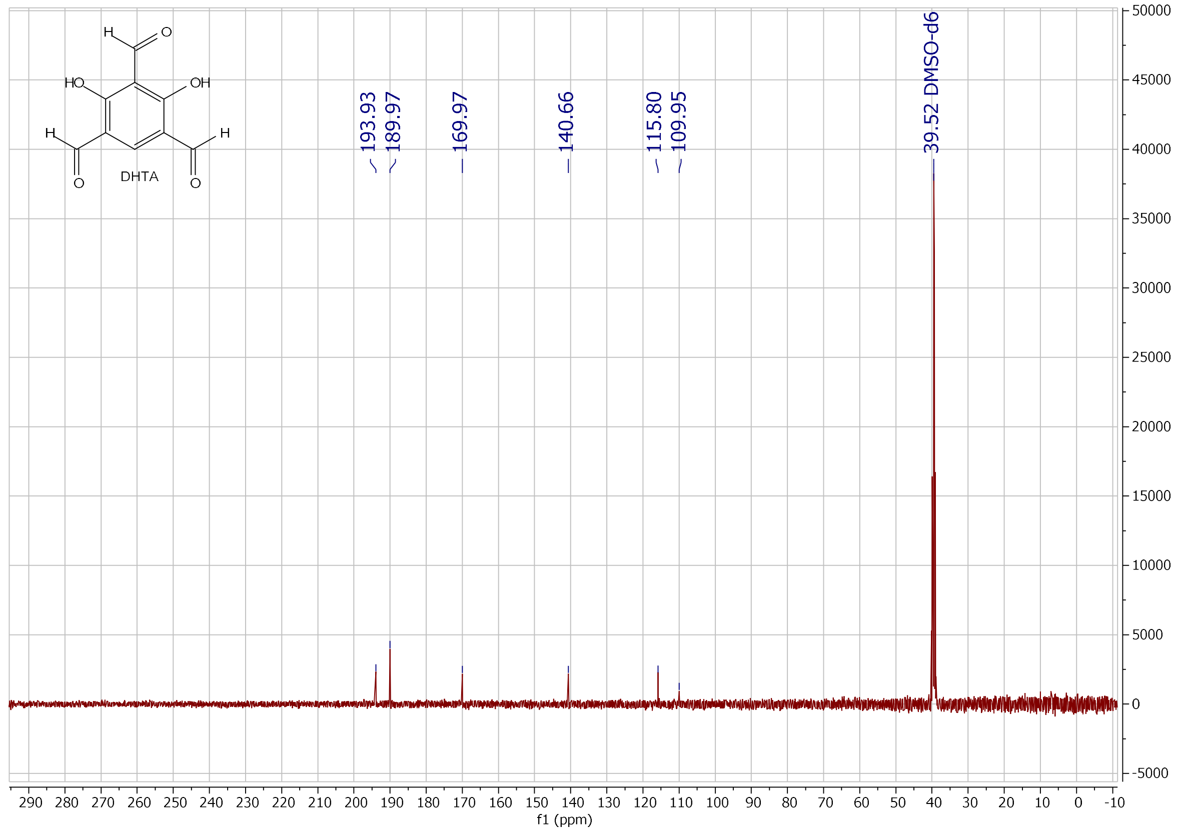

DHTA
193.93
189.97
169.97
140.66
115.80
109.95
39.52 DMSO-d6
f1 (ppm)

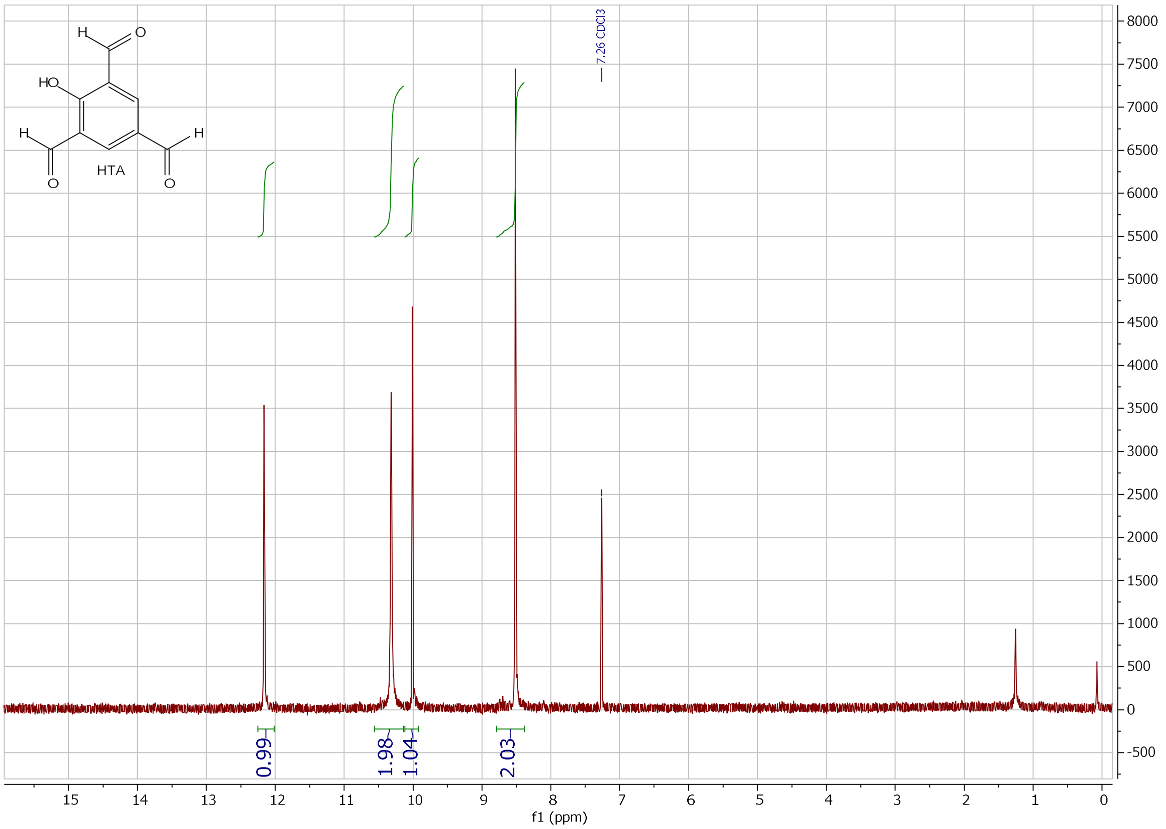
HTA
HO
7.26 CDCl3
0.99
1.98
1.04
2.03
f1 (ppm)

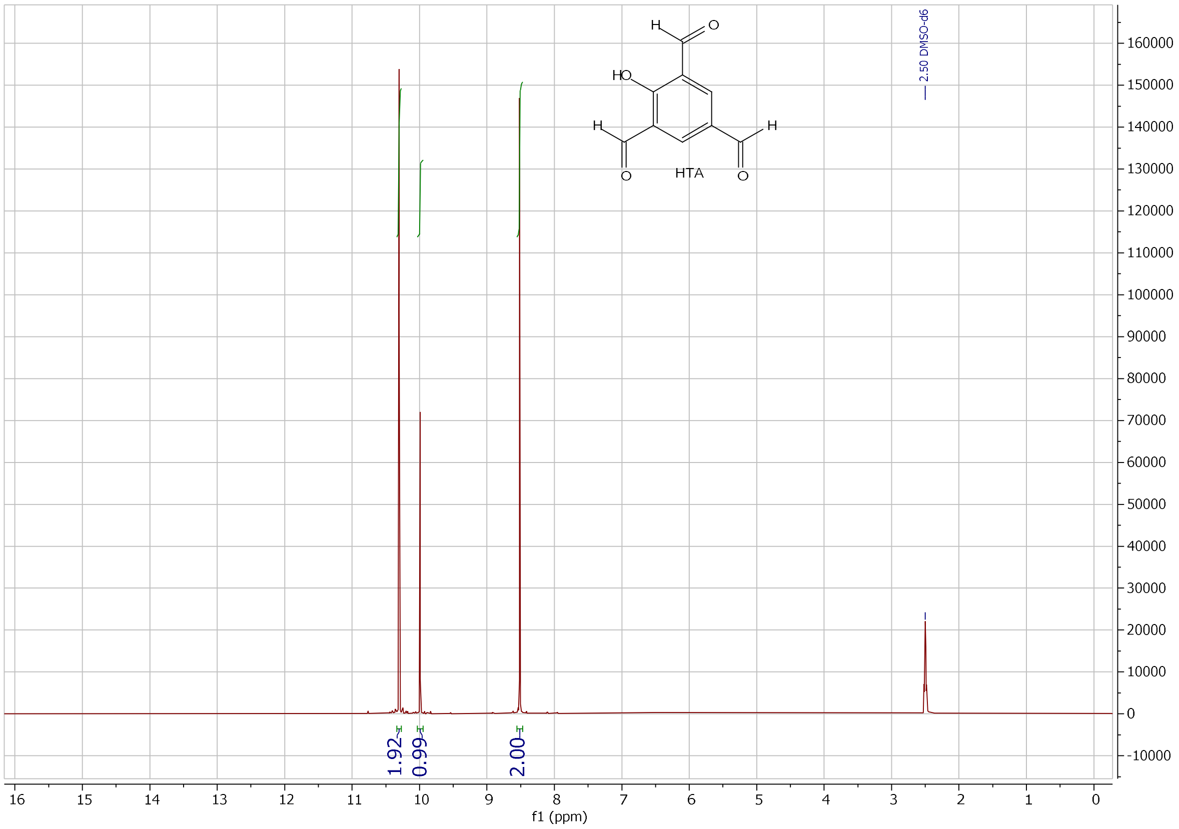
HTA
HO
2.50 DMSO-d6
1.92
0.99
2.00
f1 (ppm)

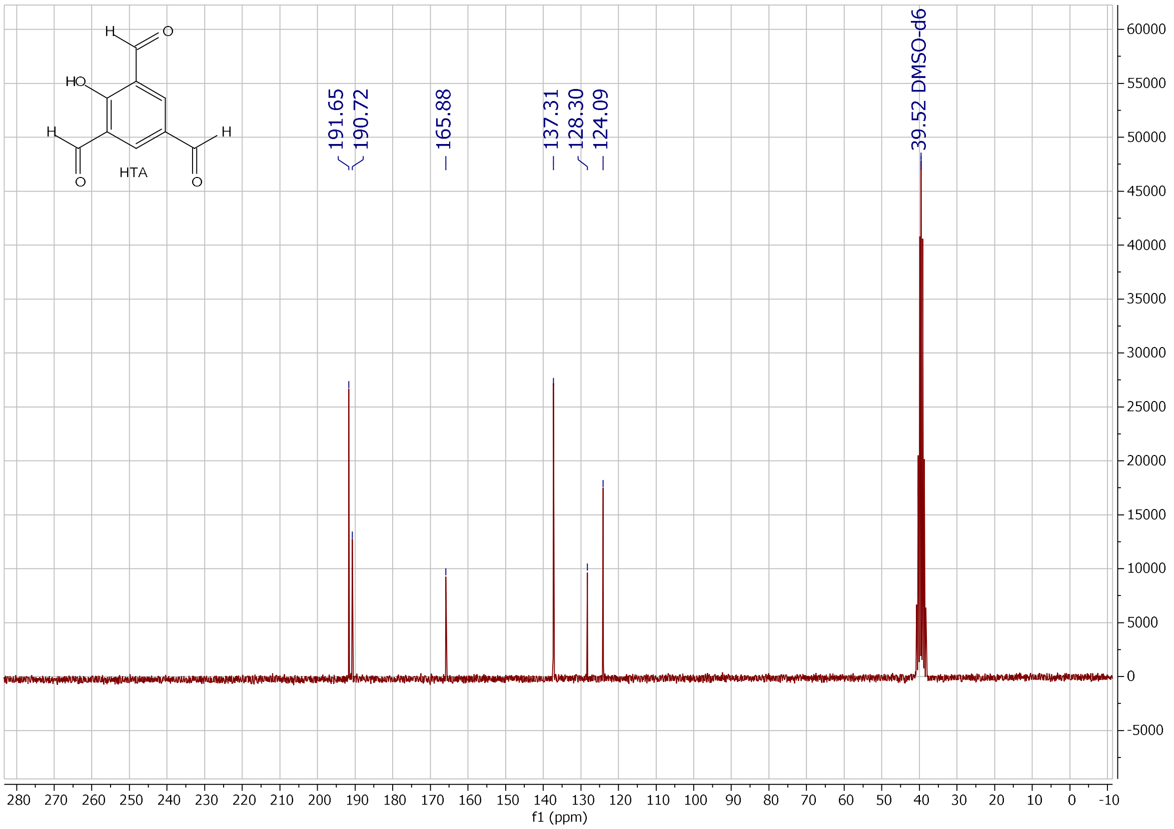

HTA
191.65
190.72
165.88
137.31
128.30
124.09
39.52 DMSO-d6
f1 (ppm)

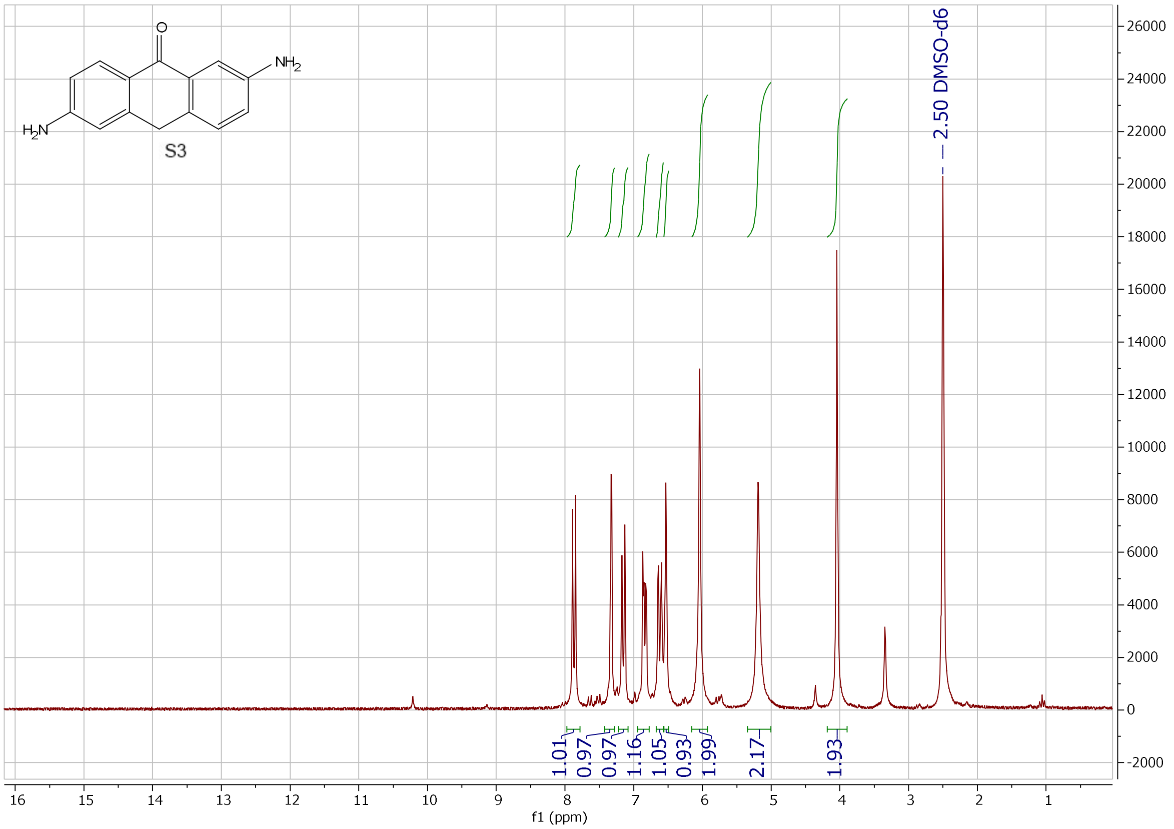

S3
2.50 DMSO-d6
1.01
0.97
0.97
1.16
1.05
0.93
1.99
2.17
1.93
f1 (ppm)

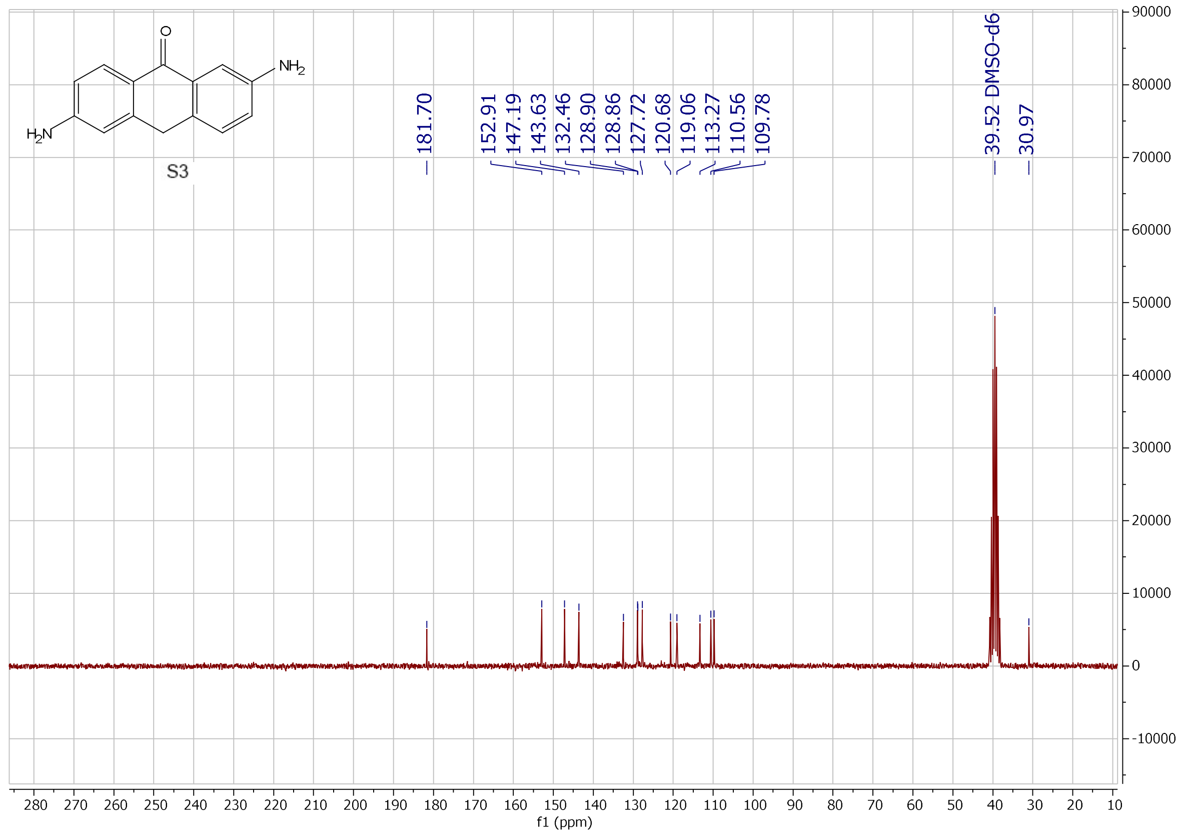
NH2
H2N
S3
181.70
152.91
147.19
143.63
132.46
128.90
128.86
127.72
120.68
119.06
113.27
110.56
109.78
39.52 DMSO-d6
30.97
f1 (ppm)

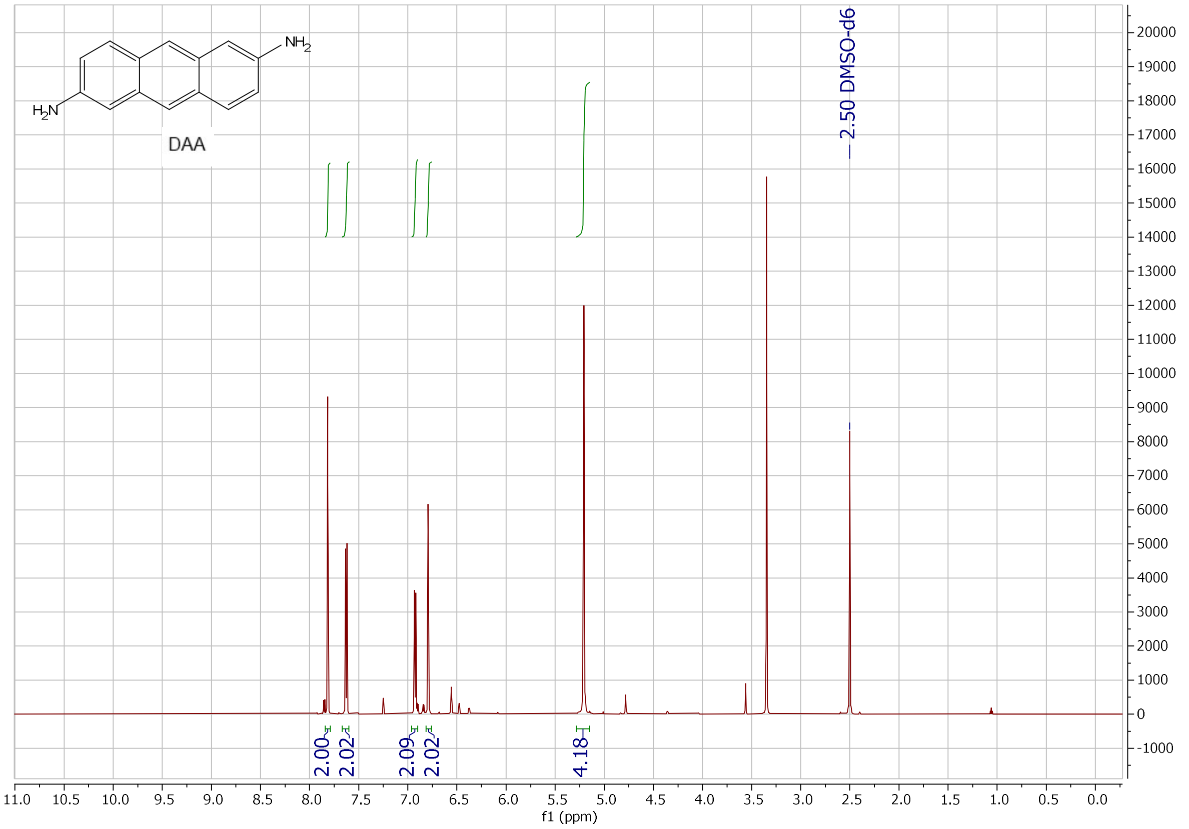
NH2
H2N
DAA
2.50 DMSO-d6
2.00
2.02
2.09
2.02
4.18
f1 (ppm)

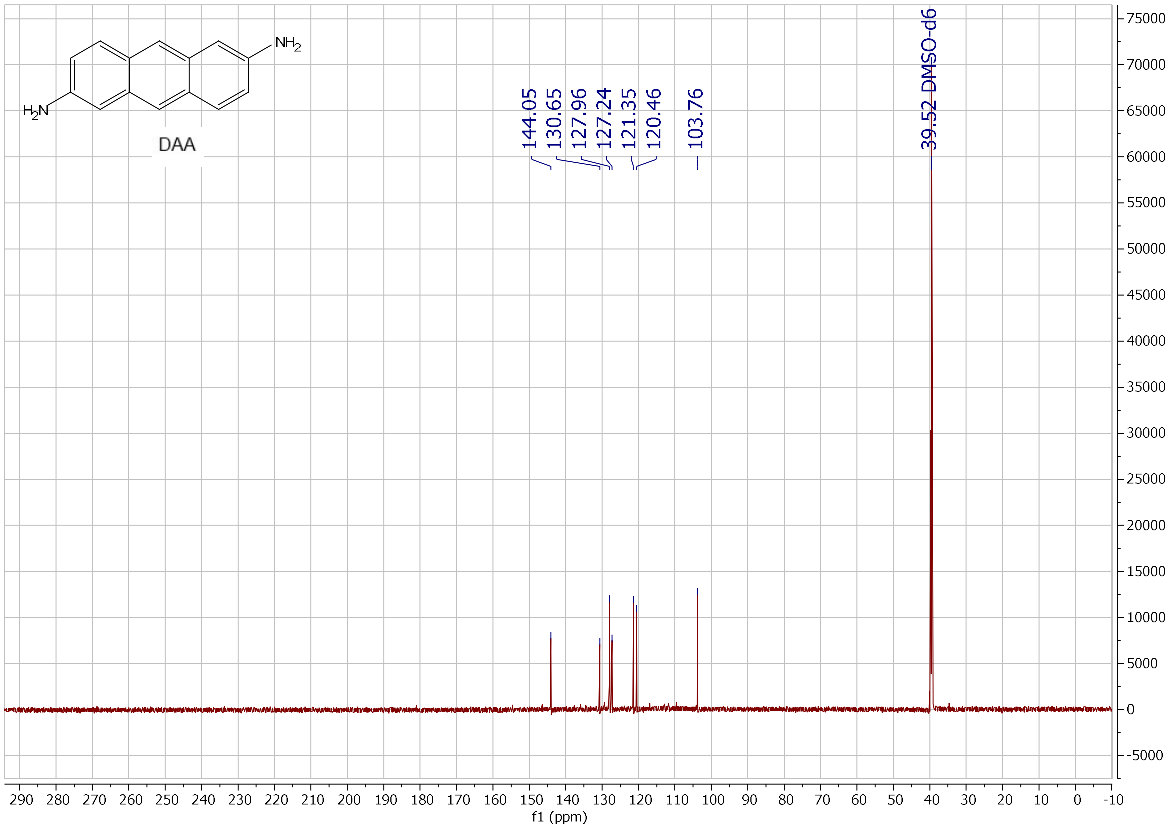

NH2
H2N
DAA
144.05
130.65
127.96
127.24
121.35
120.46
103.76
39.52 DMSO-d6
f1 (ppm)

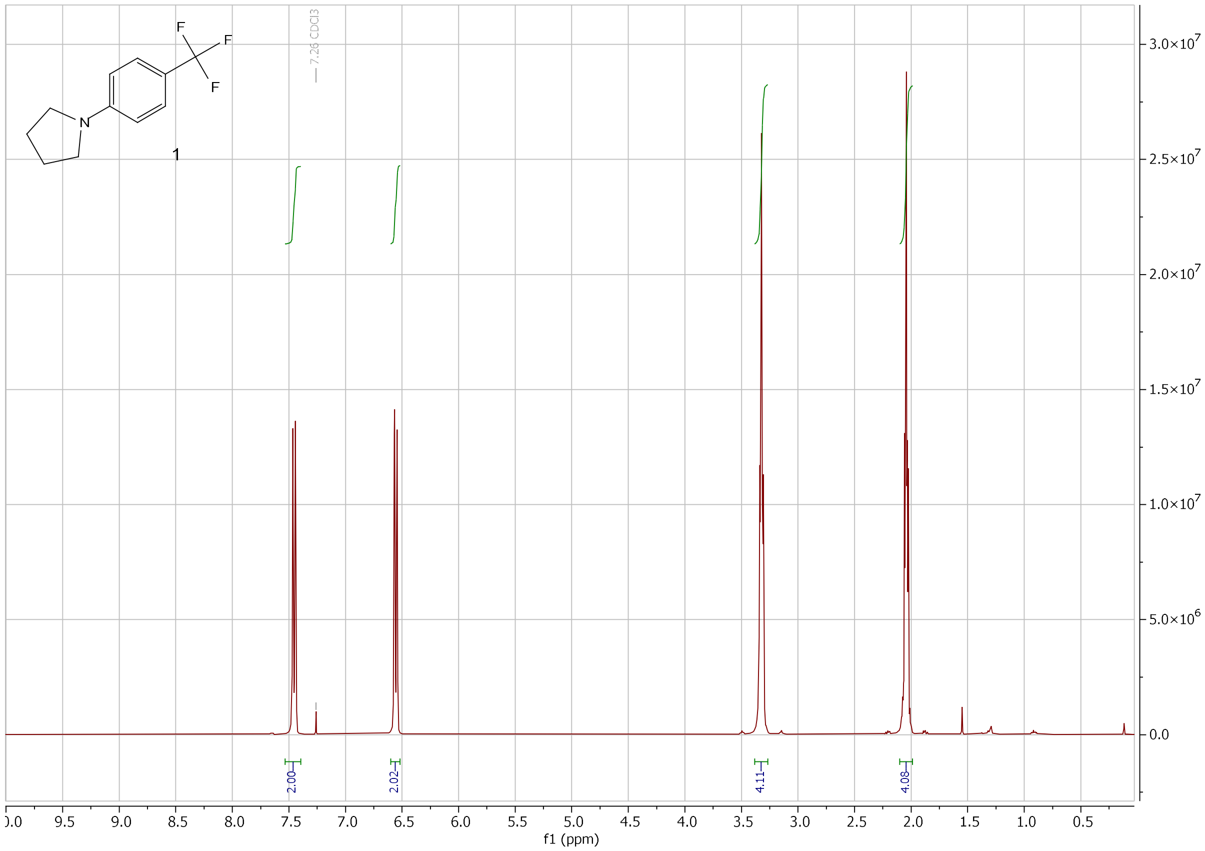

F
F
F
N
1
7.26 CDCl3
2.00
2.02
4.11
4.08
f1 (ppm)

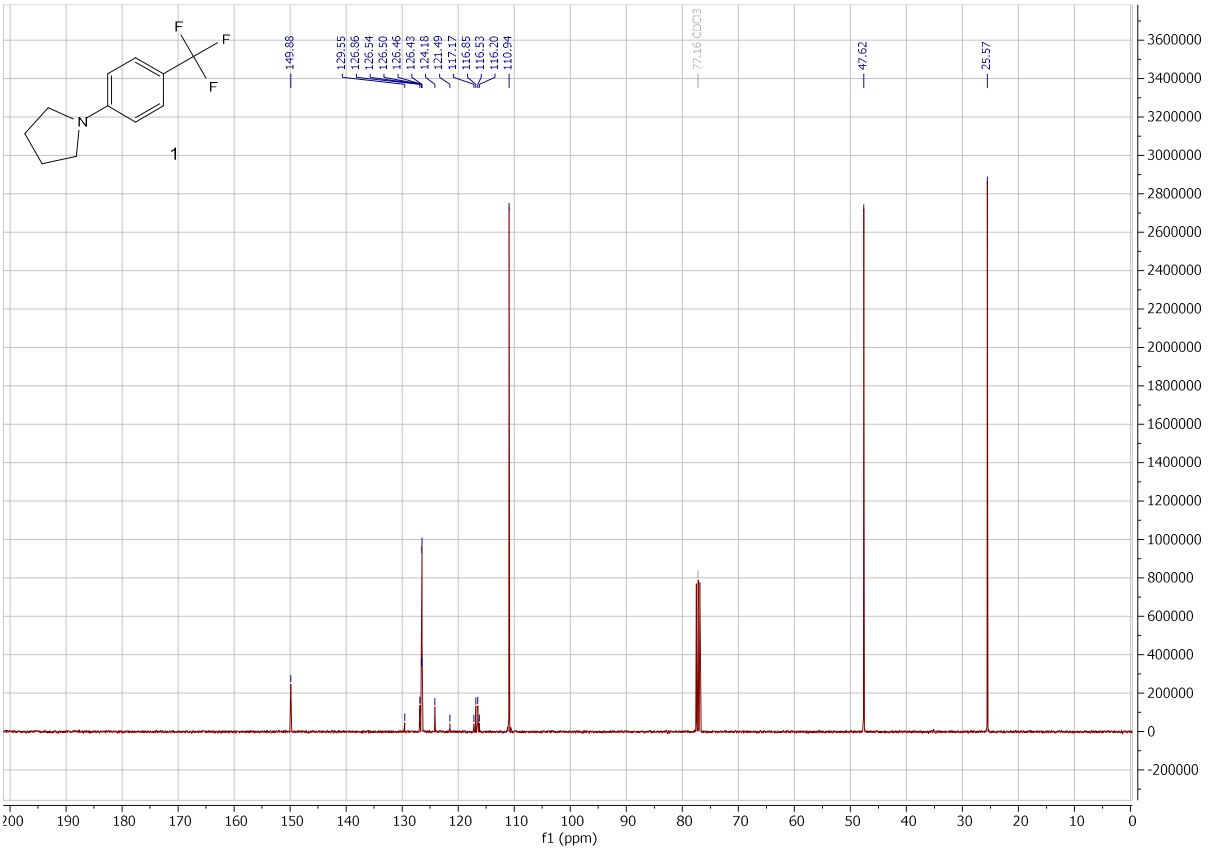
F
F
F
N
1
149.88
129.55
126.86
126.54
126.50
126.46
126.43
124.18
121.49
117.17
116.85
116.53
116.20
110.94
77.16 CDCl3
47.62
25.57
f1 (ppm)

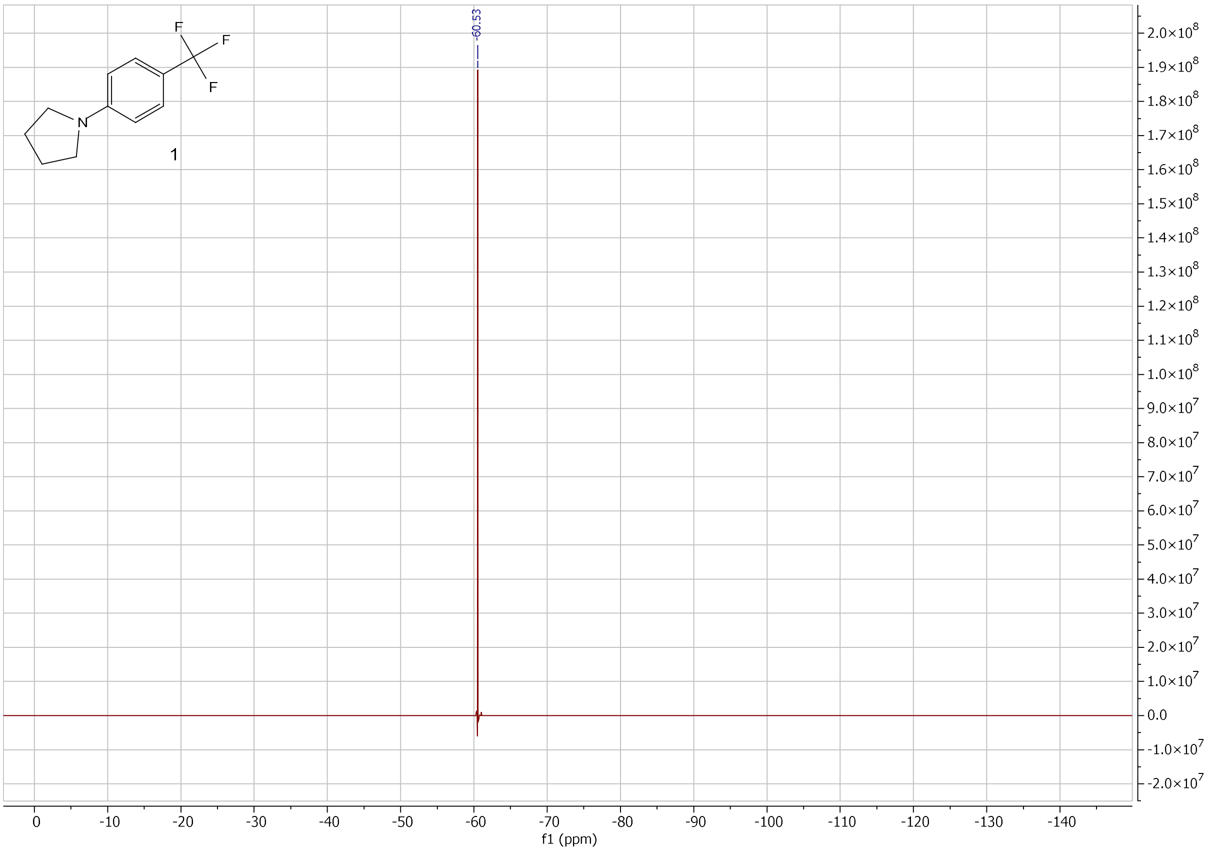
F
F
F
N
1
-60.53
f1 (ppm)

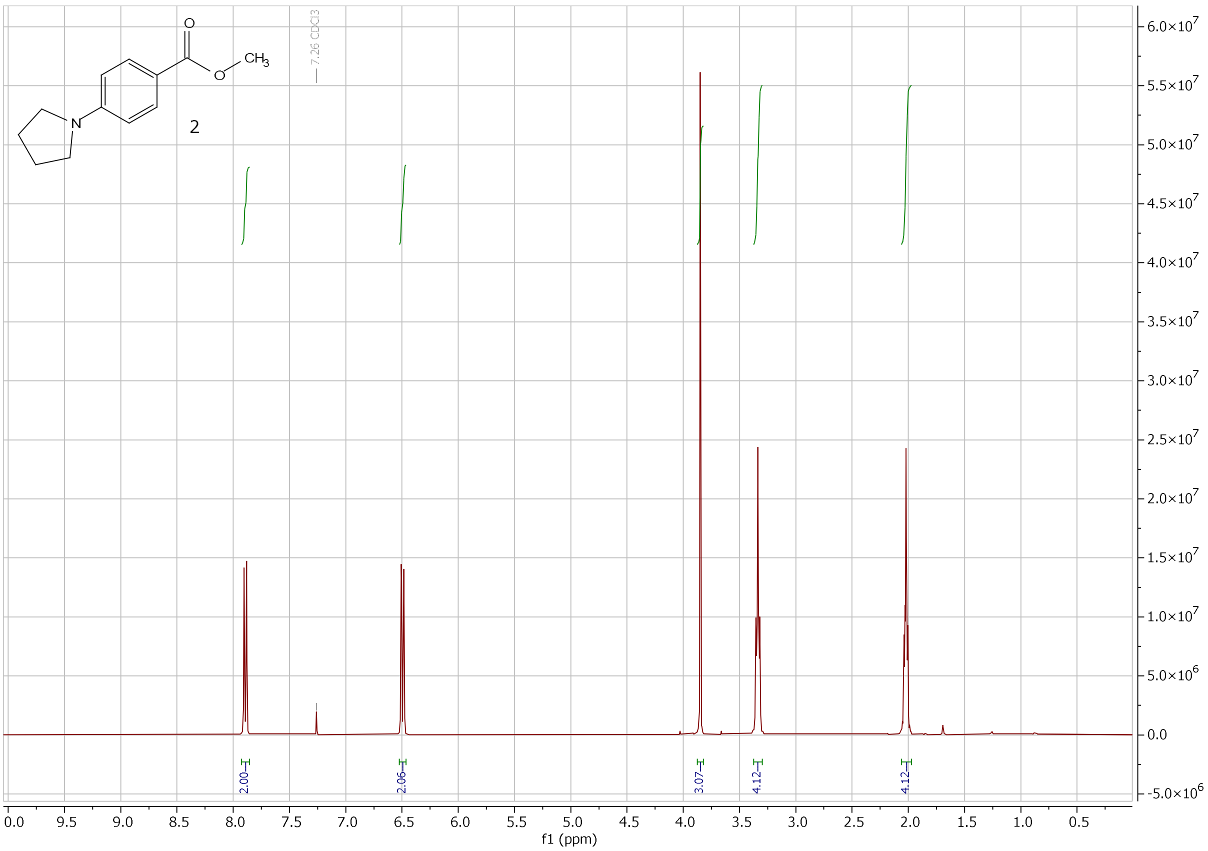
O
CH_3
N
2
7.26 CDCl3
2.00
2.06
3.07
4.12
4.12
f1 (ppm)

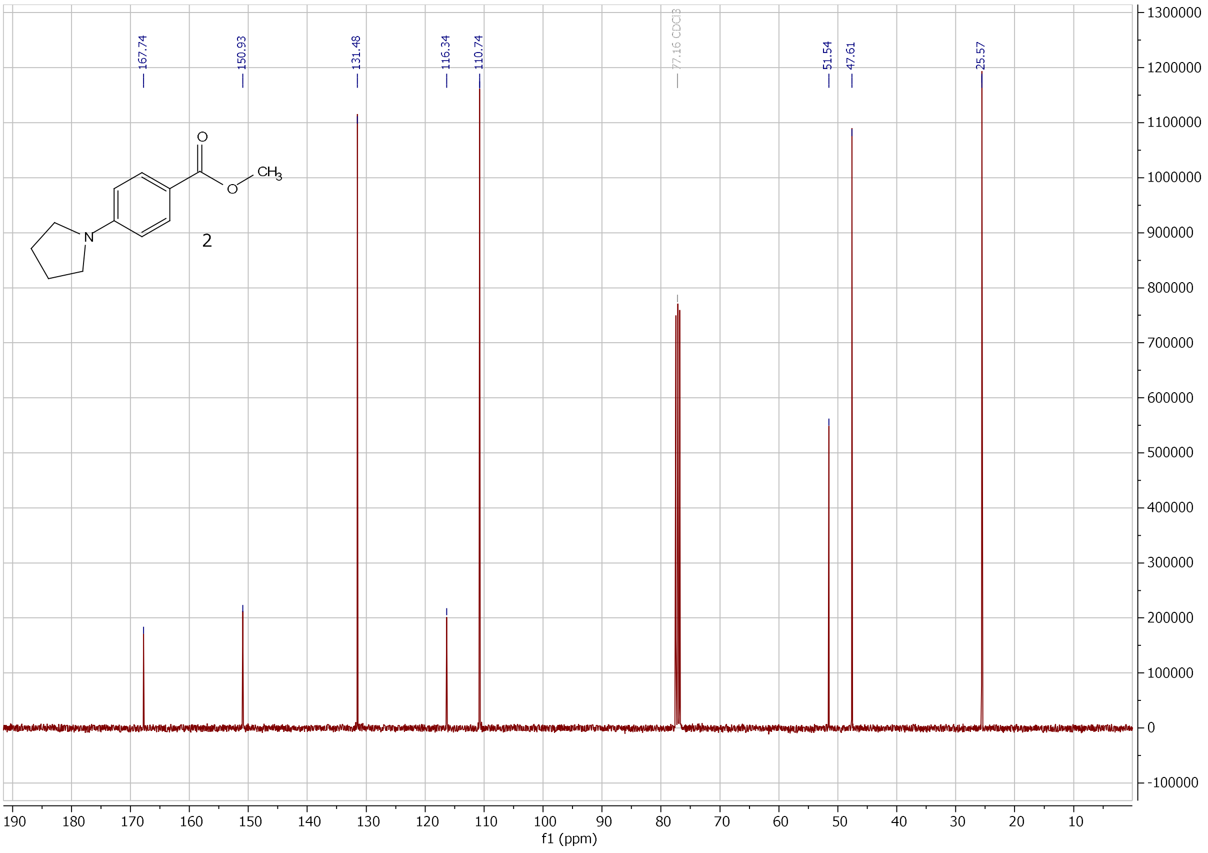
167.74
150.93
131.48
116.34
110.74
77.16 CDCl3
51.54
47.61
25.57
O
CH_3
N
2
f1 (ppm)

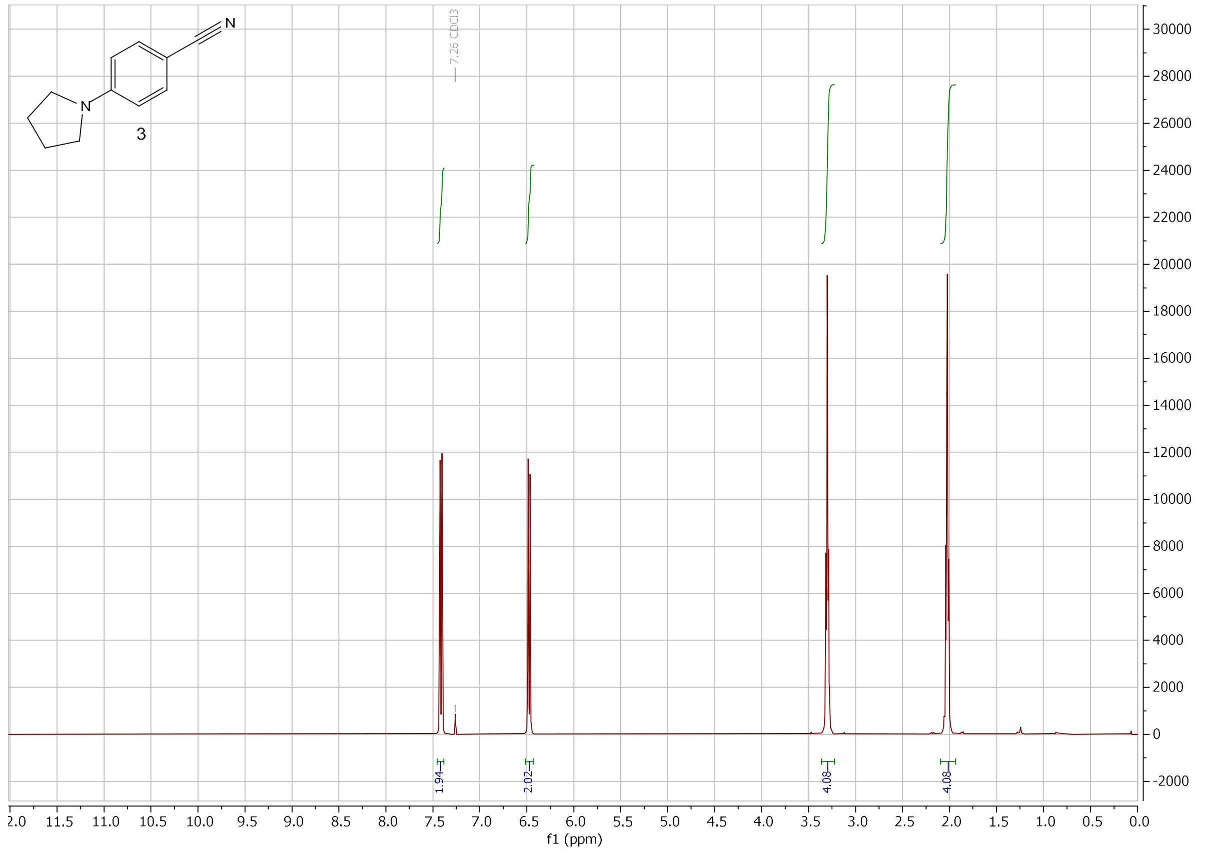

N
3
7.26 CDCl3
1.94
2.02
4.08
4.08
f1 (ppm)

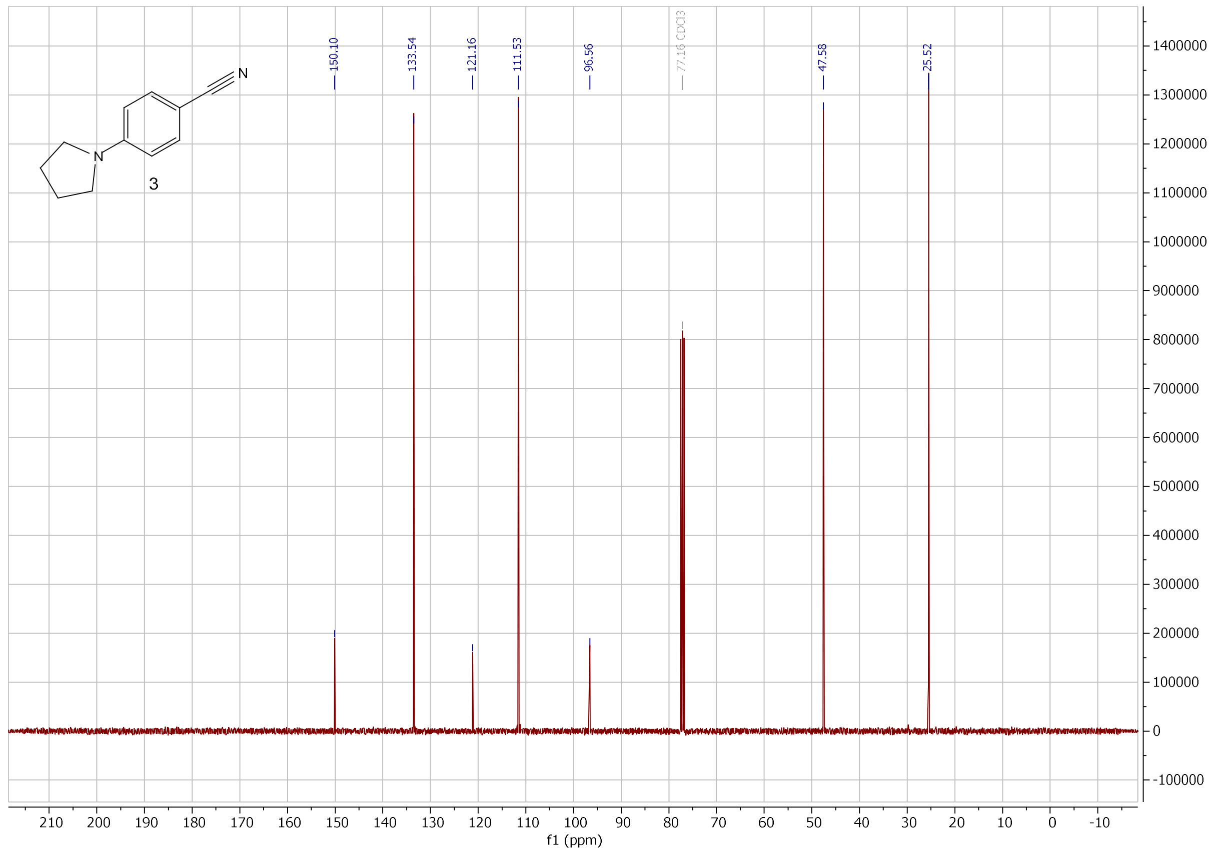

N
3
150.10
133.54
121.16
111.53
96.56
77.16 CDCl3
47.58
25.52
f1 (ppm)

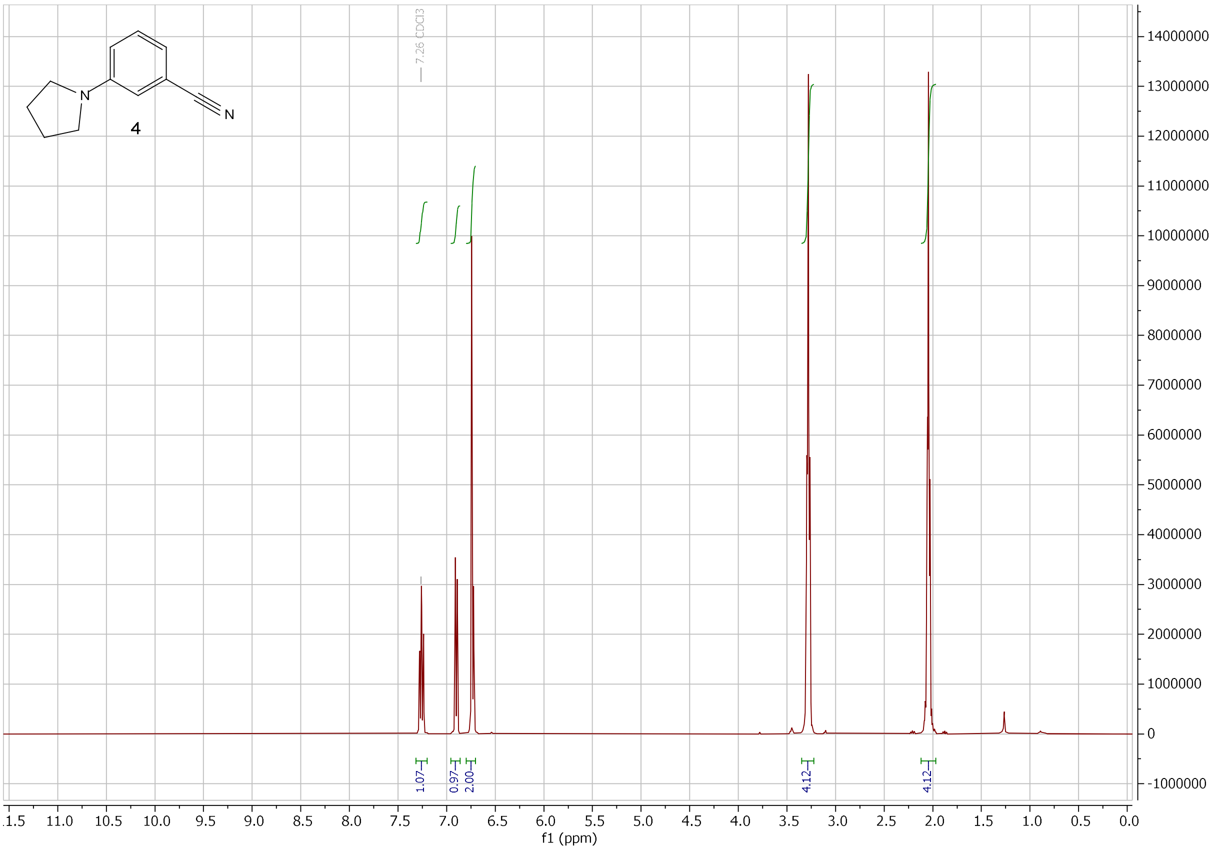

4
7.26 CDCl3
1.07
0.97
2.00
4.12
4.12
f1 (ppm)

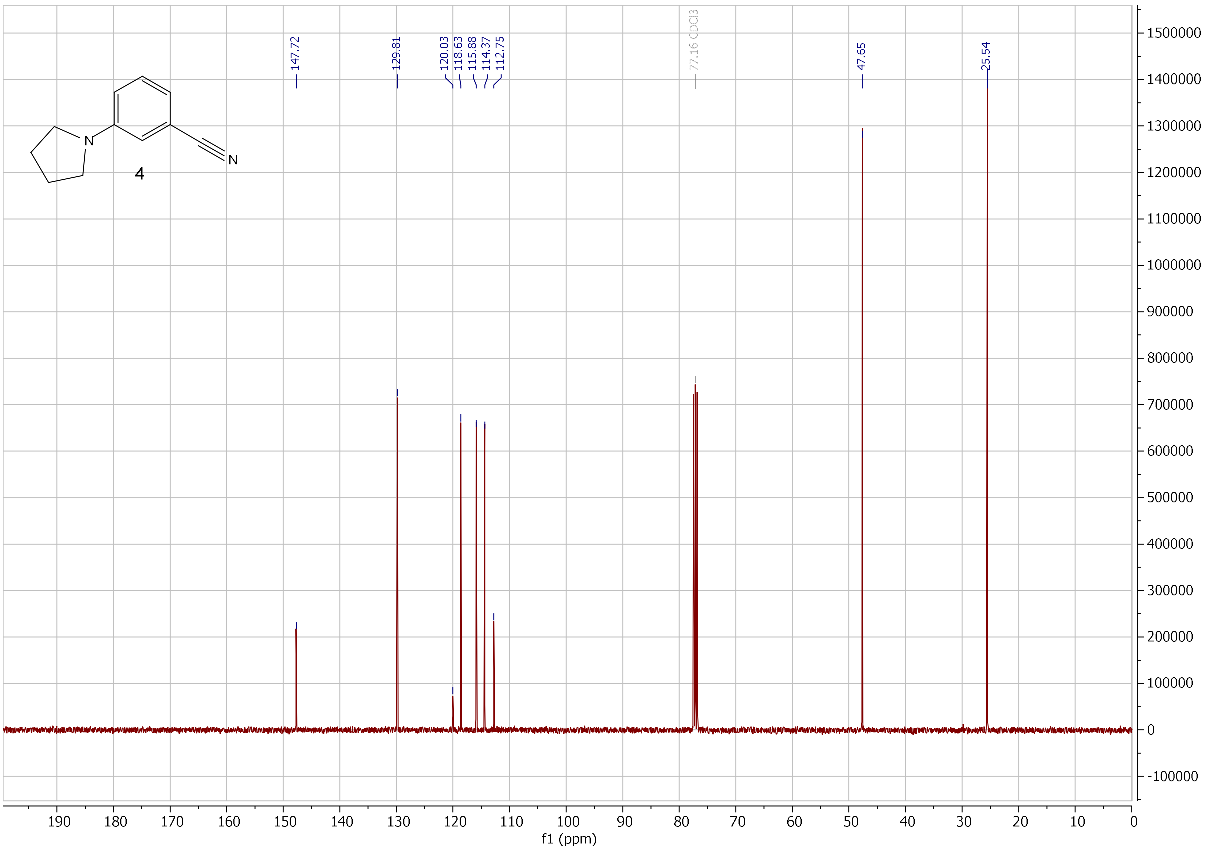

4
147.72
129.81
120.03
118.63
115.88
114.37
112.75
77.16 CDCl3
47.65
25.54
f1 (ppm)

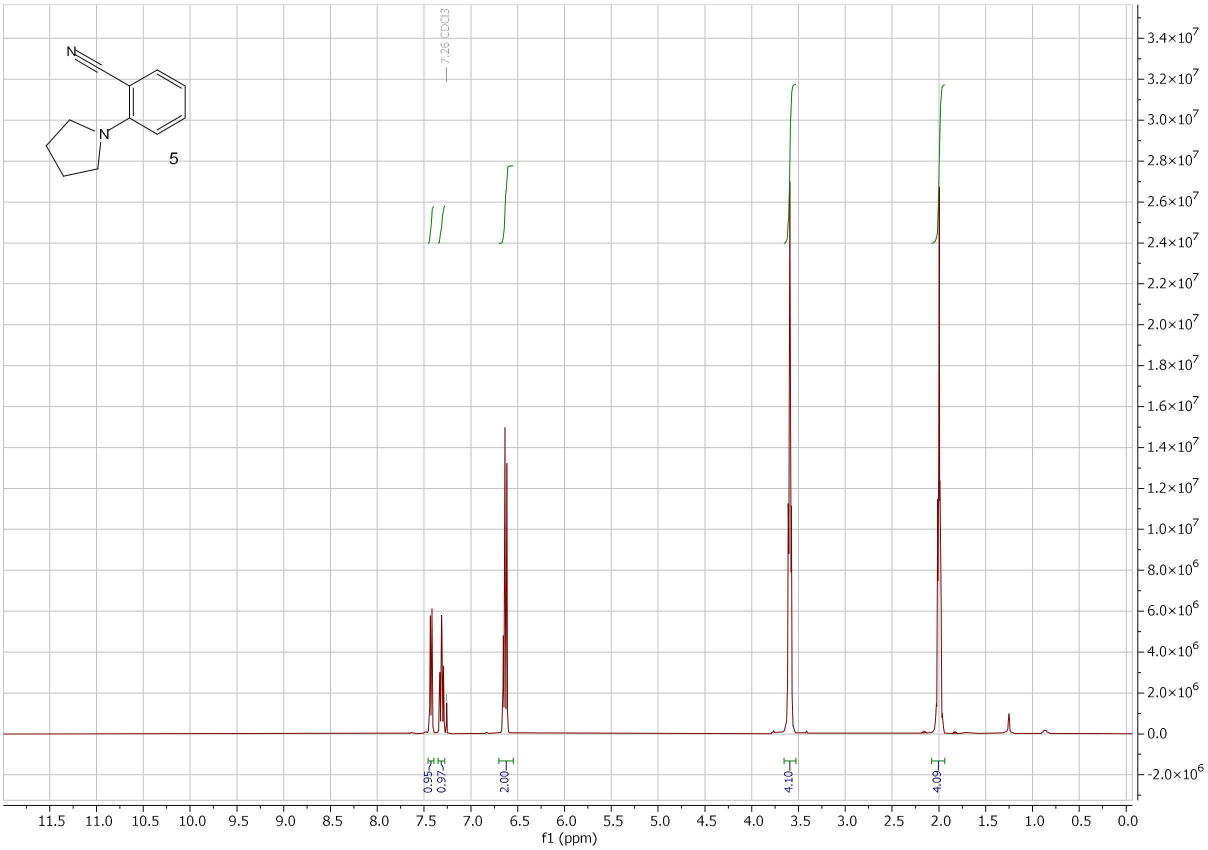
N
N
5
7.26 CDCl3
0.95
0.97
2.00
4.10
4.09
f1 (ppm)

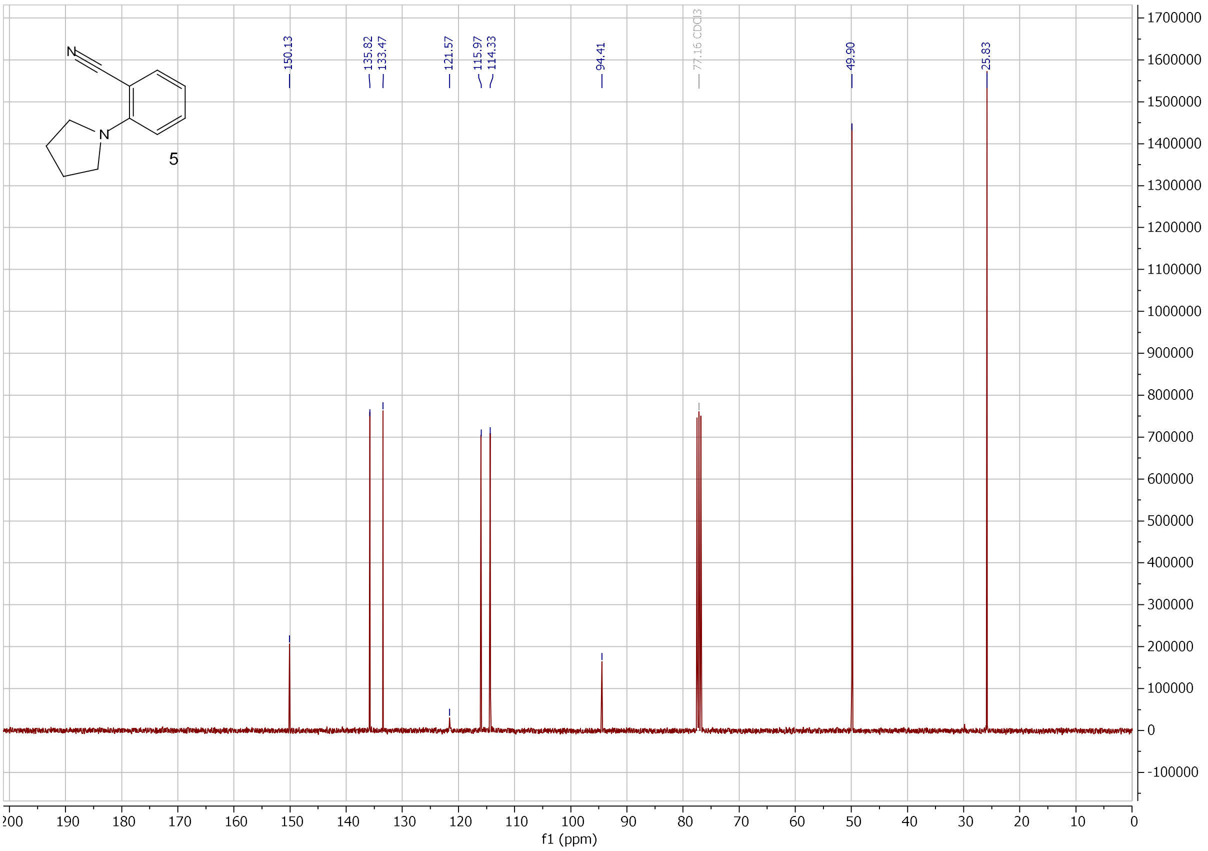
N
N
5
150.13
135.82
133.47
121.57
115.97
114.33
94.41
77.16 CDCl3
49.90
25.83
f1 (ppm)

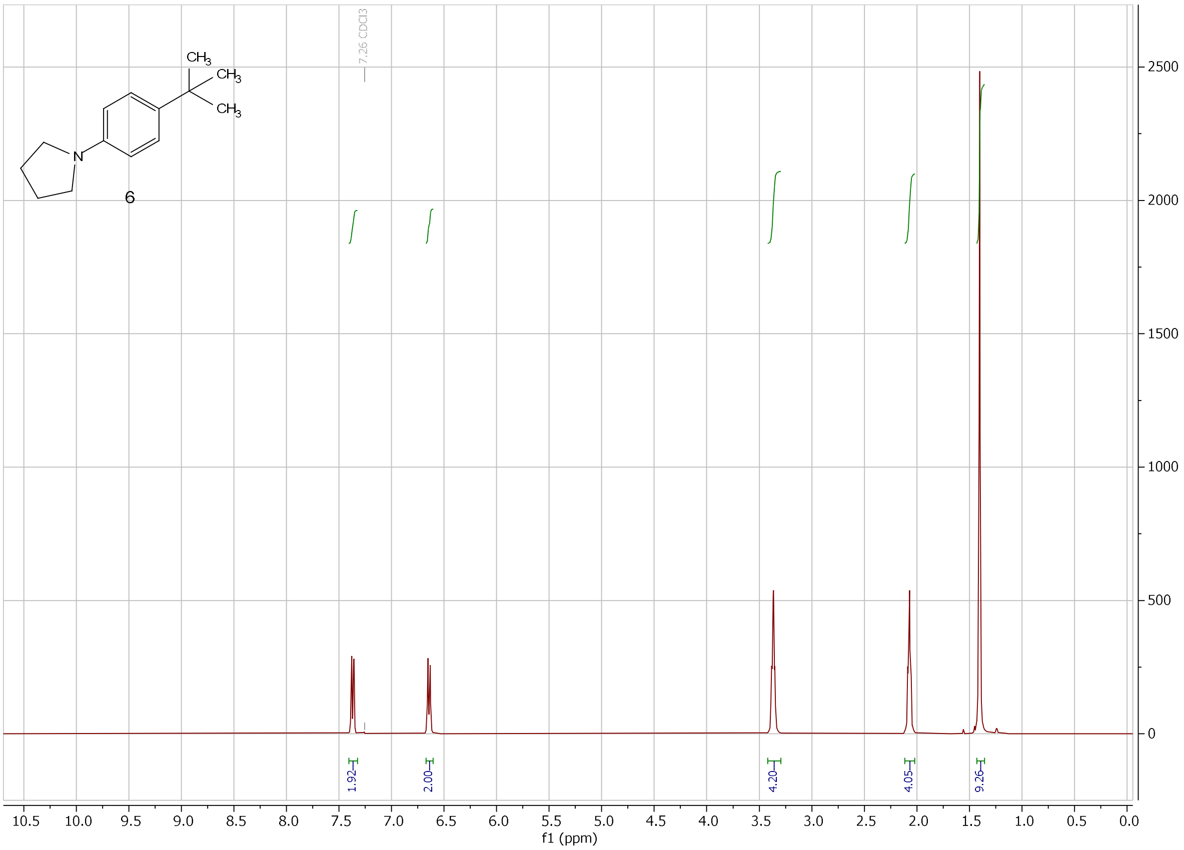
6
7.26 CDCl3
1.92
2.00
4.20
4.05
9.26
f1 (ppm)

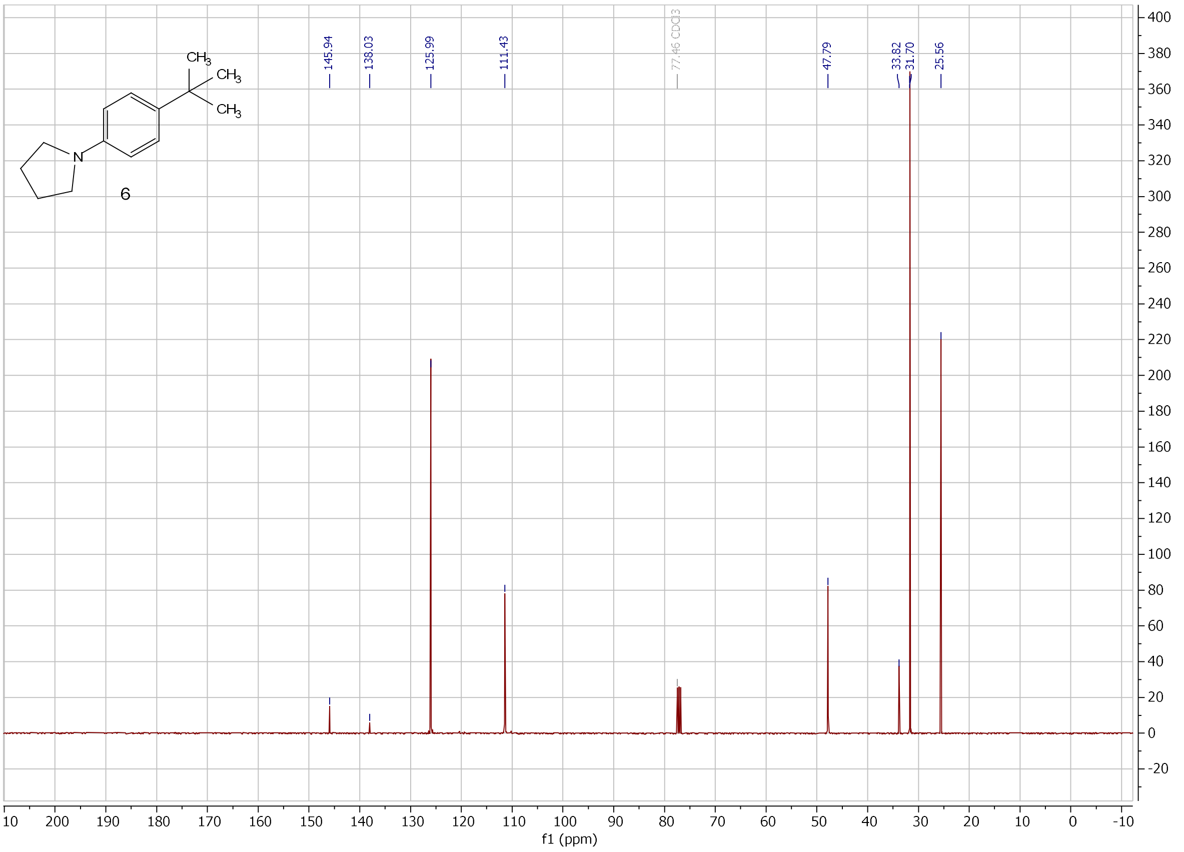
6
145.94
138.03
125.99
111.43
77.46 CDCl3
47.79
33.82
31.70
25.56
f1 (ppm)
